# Structure, Property, and Performance of Catalyst Layers in Proton Exchange Membrane Fuel Cells

**DOI:** 10.1007/s41918-022-00175-1

**Published:** 2023-03-28

**Authors:** Jian Zhao, Huiyuan Liu, Xianguo Li

**Affiliations:** Department of Mechanical and Mechatronics Engineering, University of Waterloo, 200 University Avenue West, Waterloo, ON N2L 3G1 Canada

**Keywords:** PEM fuel cell, Catalyst layer, Microstructure, Effective property, Performance, Durability

## Abstract

Catalyst layer (CL) is the core component of proton exchange membrane (PEM) fuel cells, which determines the performance, durability, and cost. However, difficulties remain for a thorough understanding of the CLs’ inhomogeneous structure, and its impact on the physicochemical and electrochemical properties, operating performance, and durability. The inhomogeneous structure of the CLs is formed during the manufacturing process, which is sensitive to the associated materials, composition, fabrication methods, procedures, and conditions. The state-of-the-art visualization and characterization techniques are crucial to examine the CL structure. The structure-dependent physicochemical and electrochemical properties are then thoroughly scrutinized in terms of fundamental concepts, theories, and recent progress in advanced experimental techniques. The relation between the CL structure and the associated effective properties is also examined based on experimental and theoretical findings. Recent studies indicated that the CL inhomogeneous structure also strongly affects the performance and degradation of the whole fuel cell, and thus, the interconnection between the fuel cell performance, failure modes, and CL structure is comprehensively reviewed. An analytical model is established to understand the effect of the CL structure on the effective properties, performance, and durability of the PEM fuel cells. Finally, the challenges and prospects of the CL structure-associated studies are highlighted for the development of high-performing PEM fuel cells.

**Graphical abstract:**

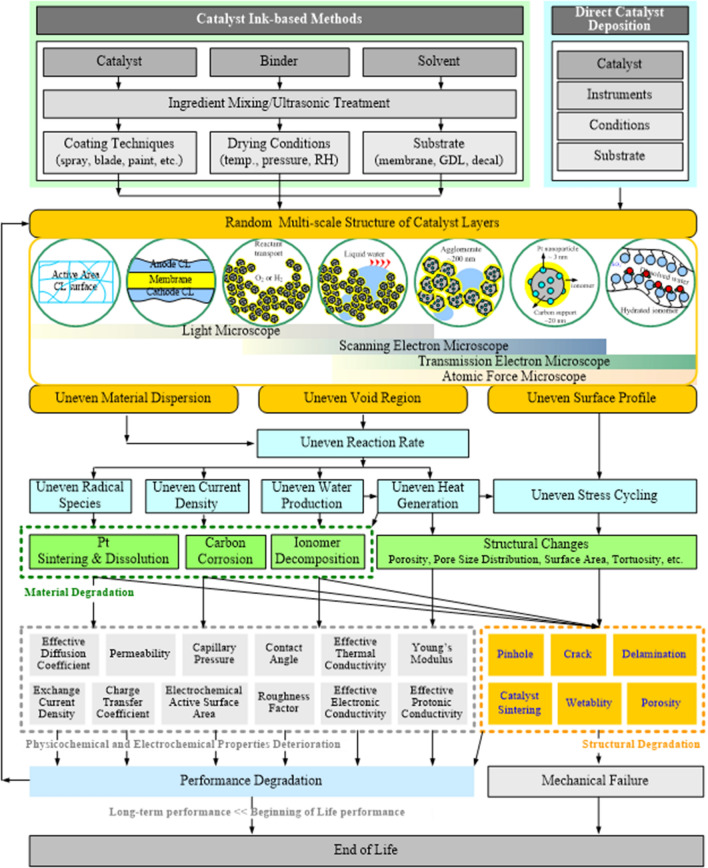

## Introduction

Cost, performance, and durability are the major barriers to the high-volume manufacturing of catalyst layers (CLs) for proton exchange membrane (PEM) fuel cells. It is estimated that the cost of CLs and their applications can be around 42% in a PEM fuel cell stack with a high-volume production of 500 000 systems per year [1]. The cost reduction of CLs can be achieved by two pathways: improving performance/durability and reducing noble catalyst loadings. However, performance, durability, and catalyst loading are usually in a trade-off relation, which requires rigorous optimizations with multiple design parameters, including the materials, formulation, and microstructure of CLs.

In a PEM fuel cell, hydrogen fuels and oxidants are supplied into the flow channels, gas diffusion layers (GDLs), and CLs. In anodic CLs, hydrogen molecules are firstly adsorbed on the catalyst surface, where the hydrogen–hydrogen bond (H–H) is broken and produces adsorbed atomic hydrogen (H*) [2]. Subsequently, each adsorbed hydrogen atom gives up an electron (e^−^) and a proton (H^+^). The generated electrons and protons will be transported by electron-conducting components (e.g., carbon supports) and ionomers, respectively, releasing the occupied catalyst surface, which is known as the hydrogen oxidation reaction (HOR). Protons are transported through membrane to cathodic CLs, while electrons are blocked by the membrane and have to move into the external circuit, where electricity is generated. In cathodic CLs, the oxygen reduction reaction (ORR) occurs via two major pathways under different conditions: dissociative and associative pathways [2, 3]. For the dissociative pathway (a.k.a. the four-electron pathway), oxygen is adsorbed by the catalyst surface, where the oxygen–oxygen bond (O=O) is broken and generates adsorbed atomic oxygen (O*). Each adsorbed oxygen atom is protonated by H^+^ and reduced by e^−^ to give the surface bonded hydroxyl (OH*) groups. The OH* can be further reduced and protonated to form water. When the water is removed from the catalyst surface, the reaction sites are released and will be ready for the next reactions. For the associative pathway (a.k.a. the peroxide or two-electron pathway), oxygen is firstly adsorbed by the catalyst surface while the O=O bond may remain unbroken. The adsorbed oxygen reacts with protons and electrons to finally form hydrogen peroxide (H_2_O_2_). Therefore, the ORR is more complicated, and generally more sluggish than the HOR [4]. It should be mentioned that water is formed at the triple phase boundary (TPB) in the cathodic CLs, where catalyst, ionomer, and reactants meet. The electrochemical reaction cannot be facilitated effectively unless most catalyst surface is concurrently accessible to the reactants, protons, and electrons, with excellent capabilities of liquid water release. Otherwise, excessive liquid water products can either occupy the reactive surface or block the reactant transport, which is known as water flooding in PEM fuel cells.

A poor selection of catalysts or the poor design of the CL structure may result in the generation of a large amount of H_2_O_2_ during the cell operation, which can attack and decompose ionomer, polytetrafluoroethylene (PTFE), or carbon supports. The most prevalent catalyst employed in PEM fuel cells is Pt based due to its excellent capability to facilitate the dissociative pathway reactions, to enhance reaction rates, and to reduce the Gibbs function of activation [5, 6]. Compared with other metal catalysts, pure Pt has a more suitable oxygen binding energy for ORR. In addition to pure Pt, substitute catalysts, including binary (e.g., PtCo), ternary (e.g., Pt–Cr–Ni), or even quaternary (e.g., Pt–Ru–Ir–Sn) Pt–transition metal alloys, are widely investigated in order to improve the ORR activity of catalysts and simultaneously reduce the cost resulted from expensive catalysts [7]. In the past decade, the mass activity of various types of Pt-based electrocatalysts has been enhanced significantly (e.g., in the range from 0.2 to 14 A mg_Pt_^−1^ [8]) by reducing particle size, controlling particle shapes, alloying Pt with transition metals, and optimizing CL formulation. However, the comprehensive performance with catalysts employed in an actual fuel cell is not improved as much as expected due to the limited reactant transport capability and low utilization of catalysts under practical operating conditions. As a result, carbon-supported Pt (Pt/C) remains the most commonly used catalyst for commercial PEM fuel cells. To further reduce the cost, many efforts have also been devoted to non-precious metal (NPM) catalysts [2, 9–15] for PEM fuel cells; however, their performance, reliability, and durability need to be further verified.

Therefore, a well-designed CL should be (1) chemically active to activate the oxygen, (2) easy to release the product water from the catalyst surface, (3) stable under corrosive operating conditions, (4) easy to transport reactants and products, and (5) easy to transport the electrons, protons, and transfer heat, which requires an optimized structure resulted from the manufacturing processes [5]. Unfortunately, the CL structure and its impact on the reaction pathways, rates, and component durability have not been fully understood, and there is still no agreement on what the best CL structure should be and how the CL structure affects the short- and long-term performance. Therefore, the understanding of the CL structure, properties, performance, and their relationships is urgently needed.

**Fig. 1:**
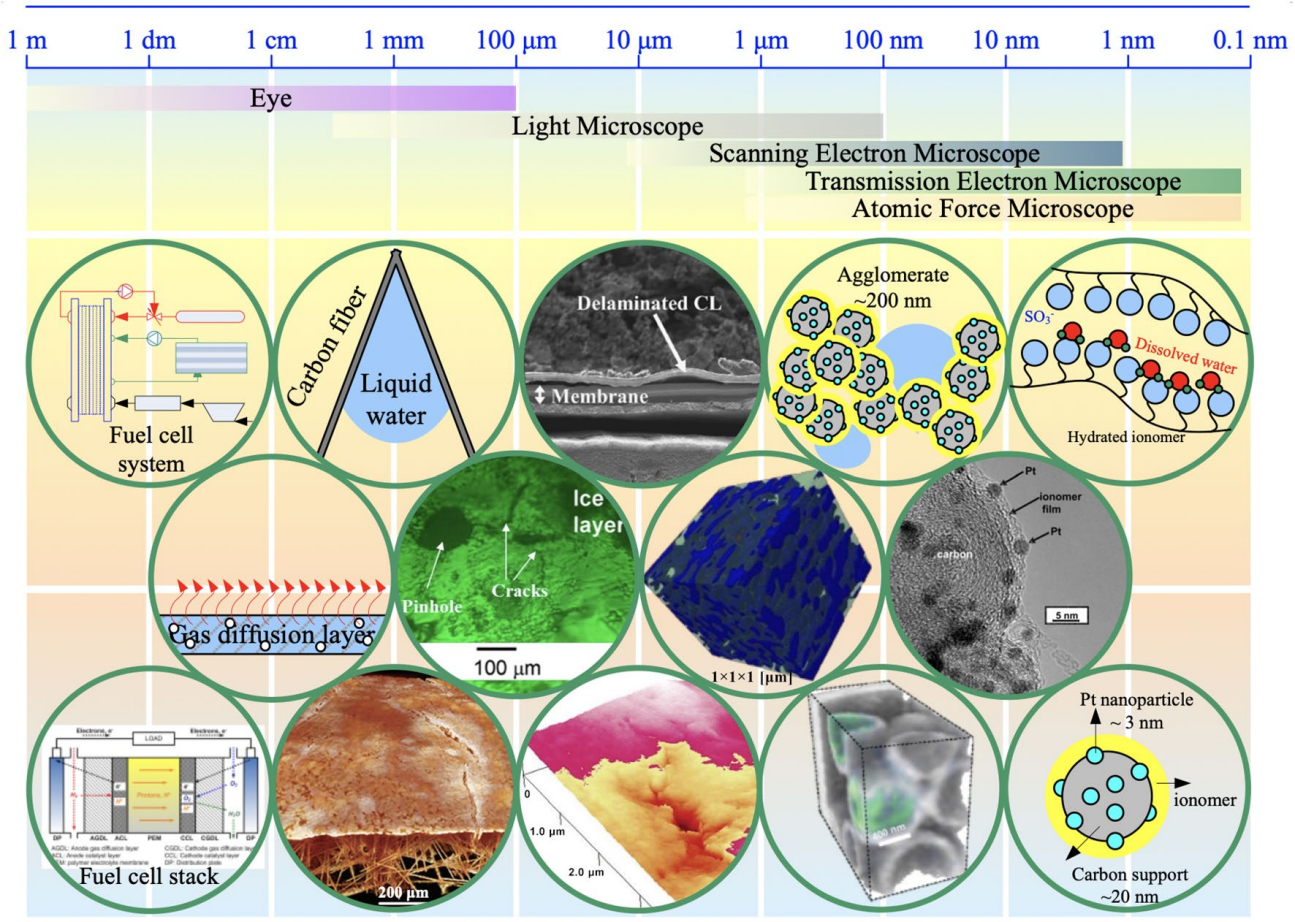
Multi-scale catalyst layer structure with representative phenomena. Adapted with permission from Ref. [16]. Copyright © 2012, Elsevier. Adapted with permission from Ref. [17]. Copyright © 2004, the Electrochemical Society. Reprinted with permission from Ref. [18]. Copyright © 2015, Elsevier. Adapted with permission from Ref. [19]. Copyright © 2013, John Wiley and Sons. Adapted with permission from Ref. [20]. Copyright © 2008, Springer Nature. Reprinted with permission from Ref. [21]. Copyright © 2007, Elsevier. Reprinted with permission from Ref. [22]. Copyright © 2006, the Electrochemical Society

The CL structure covers a wide range of length scales, involving the CL thickness from a few nanometers to tens of microns, the pore sizes at the levels of nanometers and microns, the agglomeration of the Pt/C with ionomer of a few microns, Pt particles of several nanometers, and the accompanied local reactant, water, and charged species transport within the multi-scale solid and porous structure. Examples of typical multi-scale structure-related features in CLs are shown in Fig. 1 based on the characteristic dimensions.

Many efforts have been devoted to developing low-cost, high-performance, and high-durability CLs for the PEM fuel cells; however, the CL still requires improvements to further enhance the mass transport capability and the utilization of catalyst to enhance the performance and reduce the cost of CLs for mass production. The CL performance is determined by its physicochemical and electrochemical properties, which are resulted from its structure at multi-scale levels; the multi-scale structure of the CLs will be deteriorated during the long-term cell operation, causing gradual and irreversible performance degradation. Therefore, the CL structure is of great significance for the development of electrochemical devices. In this review article, the CL structure formation, visualization, and characterization have been comprehensively reviewed, and the state-of-the-art experimental techniques and results have been scrutinized. The relation between the CL structure and its physicochemical and electrochemical properties has been reviewed along with the corresponding experimental methods for their characterization. Finally, the interconnection among the CL multi-scale structure, physicochemical and electrochemical properties, performance, and durability is examined and discussed.

## Formation, Visualization and Characterization of Catalyst Layer Structure

The practical structure of CLs, which is conventionally composed of carbon-based platinum (e.g., Pt/C), ionomer (e.g., Nafion polymer), and void regions (i.e., porous space) [3], can be determined by various manufacturing parameters, including material specification (e.g., nature of catalyst and ionomer materials, size and shape of particles, and composition), catalyst ink composition, preparation procedures, ink application techniques, and drying and hot-pressing conditions [23–25]. The development of novel catalyst and ionomer materials and the optimization of CL composition have gained significant attention, while little progress has been made to the understanding of CL structure formation and the effect on the PEM fuel cell performance. The highly random and delicate nature of CL structure typically ranging from a few nanometers to a few micrometers makes it challenging to capture all details of the CLs using the existing visualization and characterization techniques. Recent innovative fabrication methods have modified traditional CL materials and composition, e.g., plasma sputtering [26], ion-beam-assisted deposition [27], and atomic layer deposition [28], making the corresponding structure more complicated. Therefore, the major factors affecting the CL structure formation and the recent progress in advanced experimental techniques for CL structure visualization and characterization are reviewed in this section.

### Structure Formation

The CL cannot stand alone and is formed during the fabrication process, and the CL structure can be affected by many factors, including the CL ingredients, fabrication methods, procedures, and conditions, as well as the support substrates.

The CLs for PEM fuel cells made in the 1960s were composed of Pt black and PTFE with a high Pt loading of 17–45 mg cm^−2^ [29]. PTFE in the CLs not only is a binding material to stabilize the catalysts (to avoid being washed away by liquid water and reactant gas) but also improves the hydrophobicity of the CL to decrease the transport resistance of water and reactants [30]. However, the PTFE content should be optimized as excess PTFE material may cover the surface of catalyst particles, reducing protonic conductivity and active catalytic surface. To reduce the proton transport resistance, the PTFE-bounded CLs are routinely impregnated with proton-conductive Nafion polymer. However, the utilization of catalyst is still as low as ~ 20%, leading to a significant material cost, although excellent durability is observed [31].

To decrease the catalyst loading, Ticianelli et al. [32] adopted carbon-supported platinum (Pt/C) instead of the Pt black in the 1980s. The carbon supports are typically carbon black with high surface areas, such as Vulcan XC-72, Ketjen black, and Black pearls 2000. Recently, carbon supports with different morphology and sizes are actively investigated to support catalyst nanoparticles (e.g., Pt nanoparticles), including nanofiber [33], nanotube [34, 35], graphene [36], and composite supports [37]. The carbon supports can create an efficient network for electron transport between Pt surface and GDLs. The substitution of Pt/C for Pt black significantly reduces Pt loading to 0.35 mg cm^−2^ with fuel cell performance equivalent to that of CLs fabricated with Pt black [38]. Furthermore, Wilson et al. [39] employed hydrophilic Nafion polymer instead of hydrophobic PTFE material, which further enhanced the cell performance. By this means, the catalyst particles can maintain excellent contact with Nafion polymer, not only stabilizing the catalyst particles but also improving the transport of protons between the electrode and electrolyte. The binding materials with high dissolubility and diffusivity for reactant gases are favorable as the catalyst surface is often covered by a thin layer of binding materials. The gas dissolubility and diffusivity of the binding materials determine the reactant concentration on the catalyst surface, which affects the reaction rate [5, 40]. With Nafion polymer, the power density is doubled in comparison with that of the PTFE-bound CLs, and the electrochemical surface area (ECSA) is increased from 22% (PTFE-bounded CLs) to 45.4% (ionomer-bounded CLs). It should be noted that ionomer-bounded CLs are usually thinner than 50 µm with reduced overall mass transport resistance through CLs. The ionomer-bonded CL fabrication methods are often referred to as thin-film methods [41], which are widely employed in the industry.

**Fig. 2:**
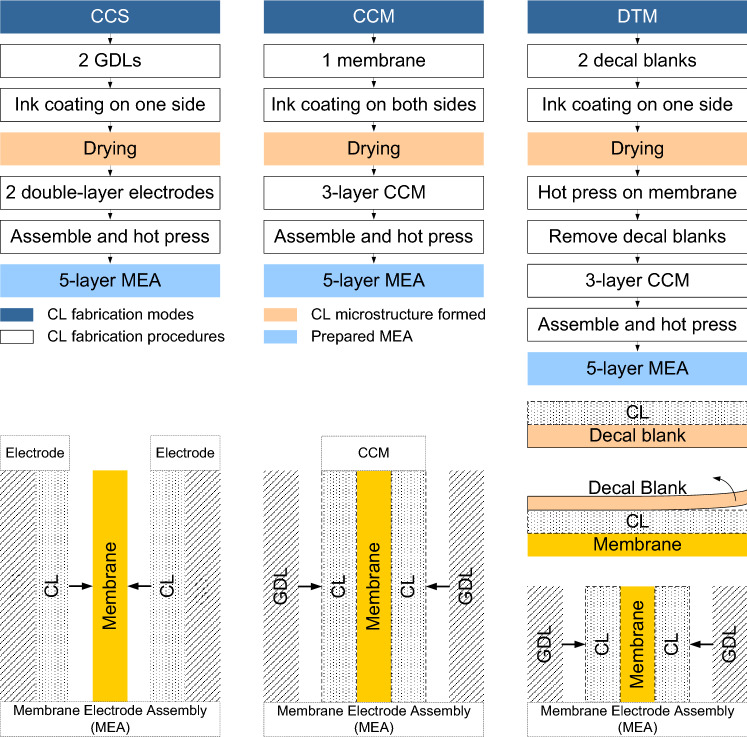
Three major approaches of the thin-film methods for the catalyst layer fabrication. Adapted with permission from Ref. [48]. Copyright © 2019, the author(s)

According to the types of coating substrates and experimental procedures, three types of thin-film methods are widely used for CL fabrications, i.e., catalyst coated on GDL substrate (CCS) [42, 43], catalyst coated on membrane (CCM) [42, 44], and decal transfer method (DTM) [41, 45], as shown in Fig. 2. For CCS methods, the catalyst ink (a mixture of Pt/C, ionomer, and solvent) is firstly coated on one side of GDL to form a gas diffusion electrode (GDE), and then, the prepared GDEs are assembled with a membrane in between to form the membrane electrode assembly (MEA) [43]. It should be mentioned that the GDL can have a two-layer structure, including a layer of PTFE-treated carbon fiber and a microporous layer (MPL) composed of a mixture of carbon particles and PTFE. The CCS method is easy for implementation; however, it remains challenging to minimize the penetration of catalyst ink into GDLs, which can cause catalyst isolation and pore blockage. Zhao et al. [42] sprayed the catalyst ink on the surface of MPLs and observed catalyst penetration into MPLs and GDLs, reduced porosity, and increased mass transport resistance. For CCM methods, catalyst ink is directly coated on both sides of the membrane, with two GDLs covering on the outer sides of CLs to form the MEA [44]. The CLs fabricated by the CCM methods demonstrate excellent interfacial properties between the CLs and membrane, resulting in superior cell performance. However, the swelling of membrane caused by the solvent has a detrimental influence on the CL microstructure; therefore, a vacuum table is often used during the fabrication process to hold the membrane in place, which increases the complexity of the manufacturing system [44, 46]. For DTM methods, the catalyst ink is coated onto a decal substrate, followed by a hot-pressing process to transfer the CLs onto the membrane to form the CCM. The DTM method often requires experienced operators or high-precision automation systems to avoid the non-uniform and incomplete transference of catalysts from substrate to membrane; thus, this method may be limited when the catalyst loading is further reduced to much less than 0.1 mg cm^−2^ [47].

**Fig. 3:**
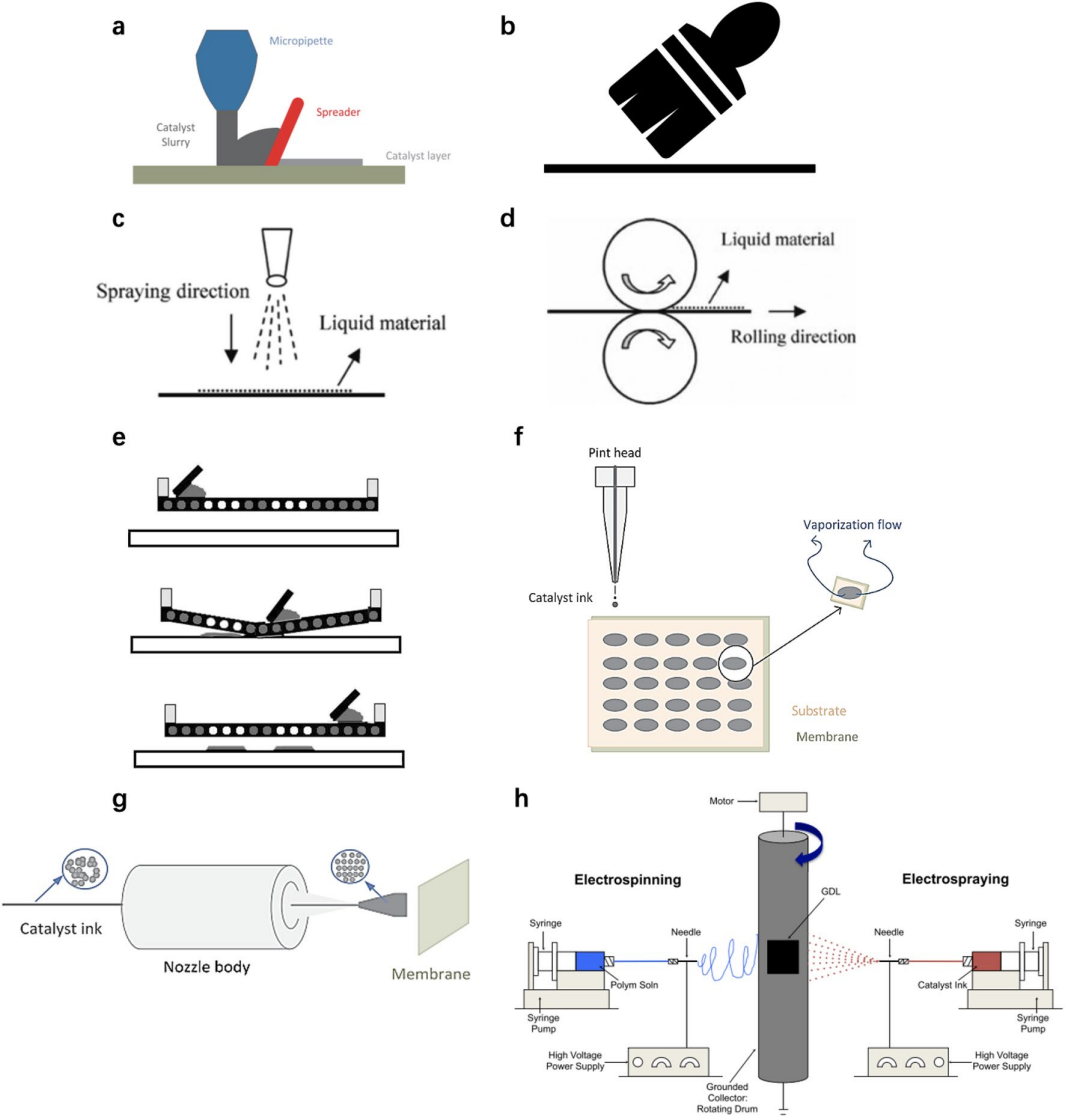
Schematic of various catalyst ink coating techniques. **a** Doctor blading. **b** Brushing. **c** Spraying. **d** Rolling. (**c**, **d**) Reprinted with permission from Ref. [54]. Copyright © 2009, Springer Nature. **e** Screen printing. Reprinted with permission from Ref. [25]. Copyright © 2011, Elsevier. **f** Inkjet printing. **g** Ultrasonic spraying. (**a**, **f**, **g**) Reprinted with permission from Ref. [65]. Copyright © 2018, Elsevier. **h** Electrospinning and electrospraying. Reprinted with permission from Ref. [63]. Copyright © 2014, Elsevier

As can be seen, the structure of the thin-film CLs is primarily formed during the ink coating process. The coating of catalyst ink can be implemented by various techniques, including blading [49–51], brushing [31], spraying [44, 52, 53], rolling [54], screen printing [55, 56], and inkjet printing [56, 57] as shown in Fig. 3a–f. Many methods have been recently developed and employed to achieve ultra-low-Pt-loading thin-film CLs, including ultrasonic spraying [58], electrospraying [59–62], and electrospinning [63], which are summarized and illustrated in Fig. 3g, h. Currently, the catalyst ink-based thin-film CLs with balanced performance, durability, and cost are the most commonly used in the industry as the catalyst loading and thickness have been significantly reduced [64].

**Fig. 4:**
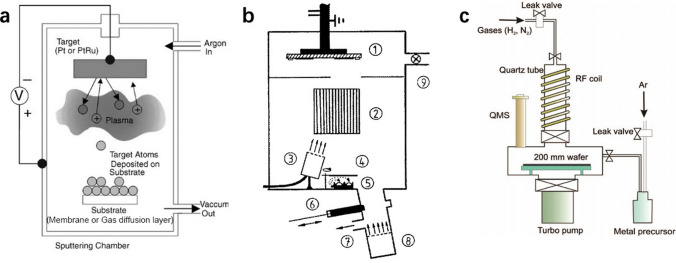
Schematic of various fabrication techniques for ultra-low-Pt-loading catalyst layers. **a** Plasma sputtering. Reprinted with permission from Ref. [26]. Copyright © 2004, Elsevier. **b** Ion-beam-assisted deposition. Reprinted with permission from Ref. [27]. Copyright © 1992, American Vacuum Society. **c** Atomic layer deposition. Reprinted with permission from Ref. [28]. Copyright © 2009, American Chemical Society

**Fig. 5:**
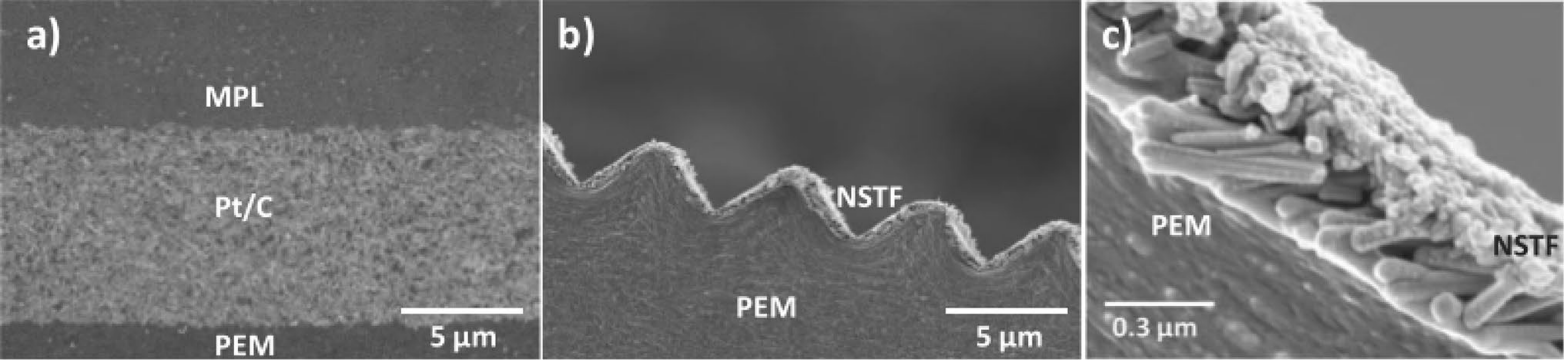
Cross-sectional scanning electron microscopy (SEM) images of **a** traditional Pt/C electrode, **b** NSTF electrode, and **c** enlarged view of NSTF electrode structure. Reprinted with permission from Ref. [78]. Copyright © 2014, the Electrochemical Society

To further reduce catalyst loading and increase catalyst utilization, direct deposition of Pt on GDLs or membrane without carbon supports and with Nafion polymer partially covered is widely investigated. Typical methods employed for direct deposition of Pt to form an ultra-thin CL (typically thinner than 1 μm with an ultra-low Pt loading of much lower than 0.1 mg cm^−2^) include sputtering deposition [66, 67], ion-beam [68–70], and atomic layer deposition (ALD) [71–74] as shown in Fig. 4. In the past decade, the order structural CLs have been actively investigated in the literature due to their excellent capabilities of minimizing Pt loadings and improving reactant transport. Yao et al. [75] developed porous Pt–Ni nanobelt arrays by following a procedure of hydrothermal processing, magnetron sputtering, decal transferring, and acid treatment. In comparison with traditional CCM methods, the new developed CLs with ultra-thin, ionomer-free, porous and oriented microstructure demonstrated better catalytic activity and mass transport capabilities. Ozkan et al. [76] developed titania nanotubes for cathode CLs. It was found that longer nanotubes (10 μm) demonstrated better performance than shorter ones (5 μm), and in comparison with photodeposition, ALD methods can create more uniform and better dispersed Pt distribution on nanotube surfaces. Murata et al. [77] developed vertically aligned carbon nanotubes for cathode electrodes, and the MEA produced superior performance of 2.6 A cm^−2^ at 0.6 V with ultra-low cathode Pt loading of 0.1 mg cm^−2^ due to enhanced transport capabilities of oxygen, protons, electrons, and water. Recently, the ionomer-free ultra-thin CLs, e.g., 3 M nanostructured thin-film (NSTF) CLs prepared by sputtering, have gained significant attention to reduce the Pt cost for PEM fuel cells with plausible stability [78]. A comparison of the NSTF electrodes and traditional Pt/C electrodes is shown in Fig. 5, demonstrating that the NSTF electrodes are much thinner and have smaller pore volume and no ionomer coverage in comparison with traditional Pt/C electrodes. However, due to the nature of hydrophilic NSTF surface, liquid water tends to accumulate in cathode CLs during the actual fuel cell testing. In addition, as no ionomer is applied in the ultra-thin layer of NSTF catalysts, the proton conductivity is relatively low. To overcome these drawbacks, a thin “interlayer” of dispersed catalysts and ionomers were applied between the NSTF layer and the MPL by Kongkanand et al. [79]. However, as the Pt loading is very low, the durability of the ultra-thin CLs may be a problem although the material cost can be reduced. Therefore, efforts have been continuously made to further improve these techniques for enhanced manufacturing efficiency and durability for industrial applications [80].

### Structure Visualization

The CL structure is complicated at different length scales from atomic to macroscale levels [81]. It is vital to visualize the multi-scale multi-dimensional structure of CLs to identify any morphology defects, to recognize the catalyst crystallinity, shape, and size, to inspect carbon agglomeration and connectivity, to check the ionomer coverage, and to understand pore structure. The typical CL thickness ranges from several nanometers to tens of microns, which often requires a combination of two or more microscopy techniques to visualize the exterior and interior structure of CLs at different scales. The commonly used microscopy techniques for CLs are reviewed in this section based on different dimensions: 2D, 3D, and 4D techniques. 2D techniques are the most commonly used for CL structure visualization from the exterior sample surface, including optical microscopy, scanning electron microscopy (SEM), transmission electron microscopy (TEM), and atomic force microscopy (AFM). The interior structure of CLs can be visualized by 3D techniques, such as focused ion-beam/scanning electron microscopy (FIB/SEM) and 3D X-ray computer tomography (3D X-ray CT). Recently, 4D techniques have been developed to obtain more detailed information about the CLs, and the fourth dimension can be chemical composition, time, temperature, or other physical parameters in addition to 3D spatial structure.

#### 2D Microscopy Techniques

The exterior structure of the CLs is commonly investigated by a variety of 2D microscopy techniques including optical microscope, SEM, TEM, AFM, and other techniques to obtain information about catalyst dispersion, carbon support connectivity, ionomer coverage, and pore structures from the surface. Table 1 summarizes the commonly used 2D microscopy techniques for CL visualization.

**Table 1:** Comparison of 2D microscopy techniques for CL solid structure characterization. (Adapted with permission from Ref. [16]. Copyright © 2012, Elsevier. Reprinted with permission from Ref. [82]. Copyright © 2012, the Electrochemical Society. Adapted with permission from Ref. [83]. Copyright © 2005, Elsevier. Reprinted with permission from Ref. [22]. Copyright © 2006, the Electrochemical Society. Adapted with permission from Ref. [17]. Copyright © 2004, the Electrochemical Society)

Method	Principle	Typical resolution	Remark	Example of CL image	Ref.
Optical microscopy	Visible lights	~0.2 μm	Suitable for observing ice coverage, pinholes with a diameter of ~ 100 μm, cracks with a few microns, dispersion of catalysts, and agglomerates of about 10 μm		[16]
SEM	Focused electron beams	~10 nm	Suitable for imaging specimens at atomic levels with a maximum resolution of 0.5 nm by focusing a beam of high-energy electrons onto the specimens (often in a vacuum condition)		[82]
Environmental SEM	Focused electron beams	~10 nm	Suitable for imaging samples in wet and gaseous environments		[83]
TEM	High-energy electrons	~0.5 nm	Suitable for catalyst particle size, shape, and dispersion of Pt nanoparticles with a tiny size of 0.33 nm, ionomer coverage, three-phase microstructure of the Pt, ionomer, and carbon		[22]
AFM	Cantilever with a probing tip	~0.5 nm	Suitable for the detection of sample surfaces at atomic levels, e.g., roughness, cracks, and holes		[17]

Optical microscopy (a.k.a. light microscopy) utilizes a system of lenses to magnify images of small objects based on visible lights with a typical resolution of ~0.2 μm. Optical microscopy is commonly used in characterizing the morphology of the CL surface, e.g., the dispersion of the catalyst, the catalyst agglomerates, pinholes, cracks, and even ice crystals, which are in the size of a few microns [16, 84]. SEM is frequently used to generate magnified images of CLs with higher resolution (around 10 nm) than optical microscopy by using focused electron beams instead of light waves as probing species [85]. SEM is very helpful and widely used for the characterization of the CL structure at the nano- and microscale levels [86, 87], e.g., micro-cracks and agglomerates of Pt/C particles in the CLs in a few nanometers [82]. Traditional SEM performs imaging in a vacuum environment for better resolution, and environmental SEM (a.k.a. ESEM) allows visualizing the samples in their natural state in wet and gaseous environments, which can be used to visualize the tiny water drops on the surface of CLs [83]. TEM is suitable for imaging specimens at the atomic level with a maximum resolution of 0.5 nm by focusing a beam of high-energy electrons onto the specimen [41]. TEM is broadly used to visualize the nano- and microstructure of the electrocatalysts and ionomer in CLs, e.g., the catalyst particle size, shape, and dispersion of Pt nanoparticles with tiny size of 0.33 nm [37], ionomer coverage [88], three-phase microstructure of the Pt, ionomer, and carbon [22]. AFM utilizes a cantilever with a probing tip to detect the surface of the specimen with a maximum resolution of 0.5 nm [89, 90]. When the probing tip scans over the specimen surface, the cantilever will be deflected in response to the forces between the tip and specimen. This technique is suitable for the detection of the sample surface at atomic levels, e.g., roughness, cracks, holes, although it is limited to recognize the interior structure of a specimen [17].

#### 3D Microscopy Techniques

Due to the complex manufacturing process, the near-surface and interior structure of the CLs may be significantly different. To investigate the interior structure of the CLs, 3D microscopy technologies have been applied to CLs to investigate their morphology and topology. The most commonly used techniques for CLs in PEM fuel cells have been reviewed in this section, such as FIB/SEM and 3D X-Ray CT.

**Fig. 6:**
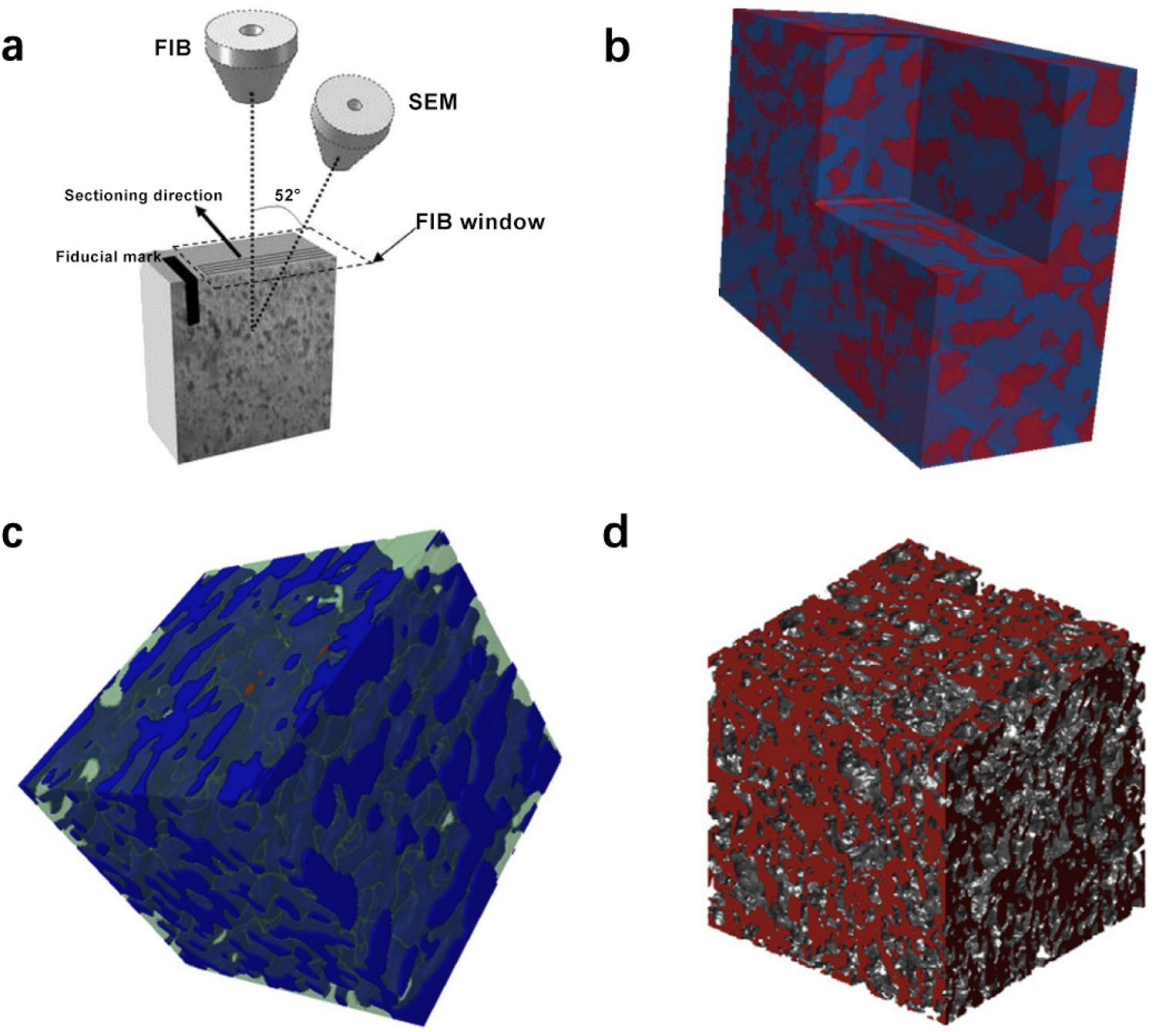
CL structure visualization by FIB/SEM. **a** Schematic of a FIB/SEM nanotomography. Reprinted with permission from Ref. [91]. Copyright © 2014, Elsevier. **b** 3D CL structure (red: pore region; blue: solid network). Reprinted with permission from Ref. [92]. Copyright © 2016, John Wiley & Sons. **c** CL structure at the resolution of 15 nm (dark blue: void; light blue: solid; red voxels: platinum). Reprinted with permission from Ref. [18]. Copyright © 2015, Elsevier. **d** CL pore structure by Inoue et al. [93]. Reprinted with permission from Ref. [93]. Copyright © 2016, Elsevier

**Fig. 7:**
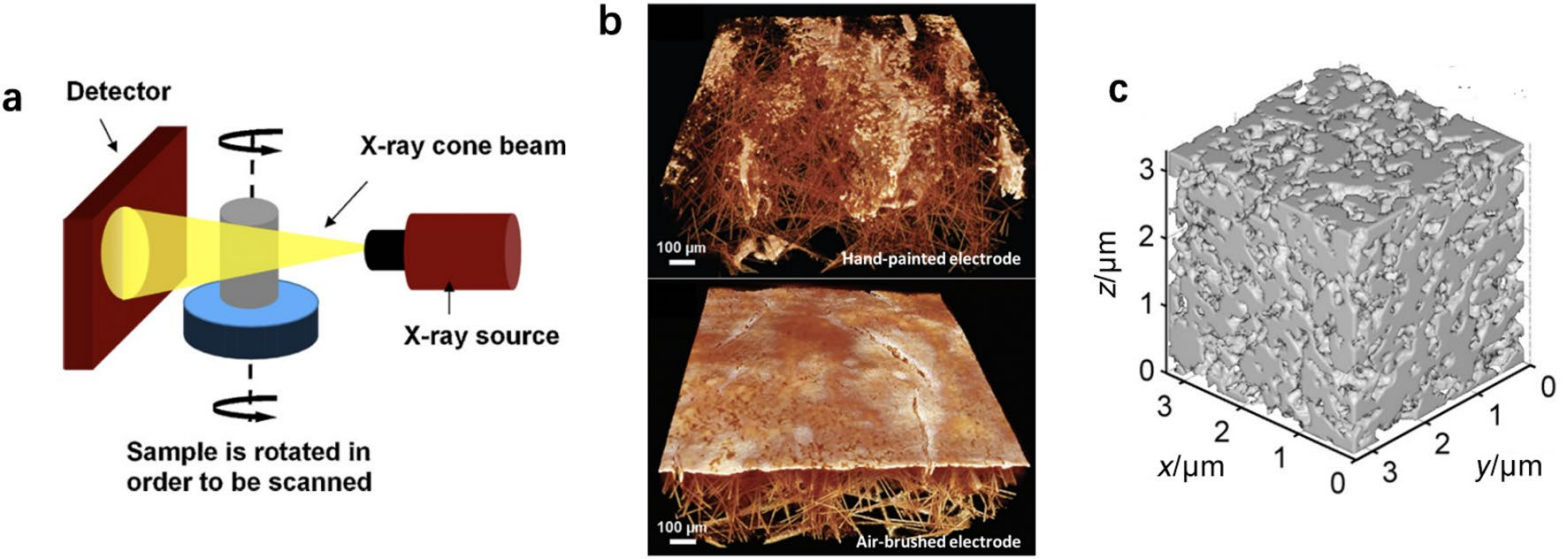
CL structure visualization by X-ray micro-tomography. **a** Schematic of an X-ray micro-tomography device. Reprinted with permission from Ref. [95]. Copyright © 2010, Elsevier. **b** Structure of hand-painted and air-brushed electrodes by X-ray CT. Adapted with permission from Ref. [19]. Copyright © 2013, John Wiley and Sons. **c** CL structure using X-ray CT by Epting et al. [97] (gray: solid; transparent: pores). Reprinted with permission from Ref. [97]. Copyright © 2012, John Wiley and Sons

1. *Focused ion-beam/scanning electron microscopy*
  FIB/SEM utilizes a focused ion beam to etch the sample and SEM to visualize the exposed interior surface, as shown in Fig. 6a. During the practical visualization process, a cubic fiducial mark is first milled on the sample. The specimen is then milled in a particular tiny thickness (e.g., 10 nm), after which SEM is used to take an image for the exposed surface. The cycling of the milling and imaging processes is repeated until a sufficient number of SEM pictures are achieved. The milling direction is often perpendicular to the ion beam, and the SEM images are aligned with the fiducial mark, which will be used to reconstruct the 3D images. The FIB window is demonstrated in the dash-line region, which protects the small fiducial mark from the FIB bombardment [91].
  Sabharwal et al. [92] reconstructed the 3D pore-solid network of the CLs prepared by inkjet methods utilizing the FIB/SEM technique, as shown in Fig. 6b. The red and blue voxels represent the void and solid regions, respectively. Based on the 3D structure, the pore size distribution (PSD) is computed and compared with experimental results, which yield good agreements. Gao et al. [18] reconstructed the CLs using FIB/SEM techniques at the resolution of 15 nm in a region of 1 μm × 1 μm × 1 μm, as shown in Fig. 6c. The dark blue, light blue, and red voxels represent the void, solid, and platinum, respectively. Inoue et al. [93] combined continuous 2D cross-sectional SEM images to form the 3D structure of the CLs using FIB/SEM, and the interior solid and pore structure of CLs can be visualized as shown in Fig. 6d. It should be noted that FIB/SEM is a destructive method to visualize the interior structure of a specimen by etching the solid materials, meaning that the samples will be damaged after imaging using this method. Other disadvantages include the lack of visible areas, curtaining artifacts resulted from different milling speeds at the material and pore phases, as well as the heat generated during the imaging process [94].
2. *X-ray computer tomography*
  X-ray CT is a nondestructive and noninvasive visualization method to detect the interior characteristics of a solid or porous material. X-ray tomography devices are typically composed of an X-ray source and a detector, as shown in Fig. 7a. The photons generated by the X-ray source pass through the specimen and a portion of photons that are not absorbed by the specimen will be collected by a photon detector, where the X-rays are converted to visible lights. The visible lights are further converted to an electric current that can be used to generate digital images. The specimen is often rotated to obtain multiple 2D projected images, which can be used to reconstruct a 3D image [95].

Hack et al. [96] utilized the X-ray CT technique to visualize the 3D structure of the cathode electrode, Nafion membrane, and anode electrode, which are prepared by two different methods: hot pressed and not hot pressed before and after accelerated stress tests. According to the top cathode CL surface of the end-of-test (EOT) images, it is observed that the CL is delaminated from the membrane for the non-hot-pressed CLs, which increases the interfacial resistance to proton flow. Jhong et al. [19] studied the structure of CLs coated onto GDLs with hand-painting and air-brushing, as shown in Fig. 7b. It is found that the structure of the CLs in the electrodes is quite different: in the hand-printed electrodes, the catalysts penetrated through the cracks of supporting GDLs, while in the air-brushed electrodes, the CL is uniformly coated on the GDL surface. The different structures of the CL fabricated by different coating methods result from the rapid evaporation of the solvent in catalyst ink during the atomization at the air-brush nozzle and GDL surface. Epting et al. [97] visualized the structure of CLs with a volume of 3 μm × 3 μm × 3 μm using X-ray CT techniques, as shown in Fig. 7c. However, unlike the FIB/SEM technique, the X-ray CT is difficult to distinguish the ionomer film covered on the catalyst particles. Moreover, it should be noted that the porosity obtained by analyzing images from TEM, FIB/SEM, or X-ray CT is lower than the calculated porosity based on the composition and thickness. The discrepancy is likely resulted from the micropores that cannot be detected by these imaging techniques. It should be pointed out that the quality and accuracy of the 3D images rely on not only the experimental methods but also the microstructure reconstruction methods, even though the quantitative analysis of the effect of the algorithm on the reconstruction accuracy is limited. Further, the spatial resolution of X-ray CT is still not sufficient to study single agglomerates.

#### 4D Microscopy Techniques

In addition to three spatial dimensions, the information about the chemical composition [98], temperature [99], and time-dependent structure changes [100, 101] of the CLs in PEM fuel cells has become more and more important to fundamentally scrutinize the local transport, electrochemical, and degradation phenomena. The combination of the additional one dimension with the 3D geometrical data is often referred to as 4D imaging [102, 103]. For instance, Wu et al. [98] utilized a multi-energy X-ray spectro-tomography technique to investigate the 3D distribution of chemical species of the cathode CL for PEM fuel cells. The chemical map of each component in the specimen is taken at multiple angles and quantitatively converted to carbon support or ionomer, and the images of the chemical map are then aligned to form 3D images for each component. By combining the 3D datasets of ionomer and carbon particles, 4D (or chemically sensitive 3D) images are generated. It should be noted that the exposure time of the CLs in the imaging instrument should be well controlled to avoid potential ionomer damages, which may distort the actual CL structure [98].

**Fig. 8:**
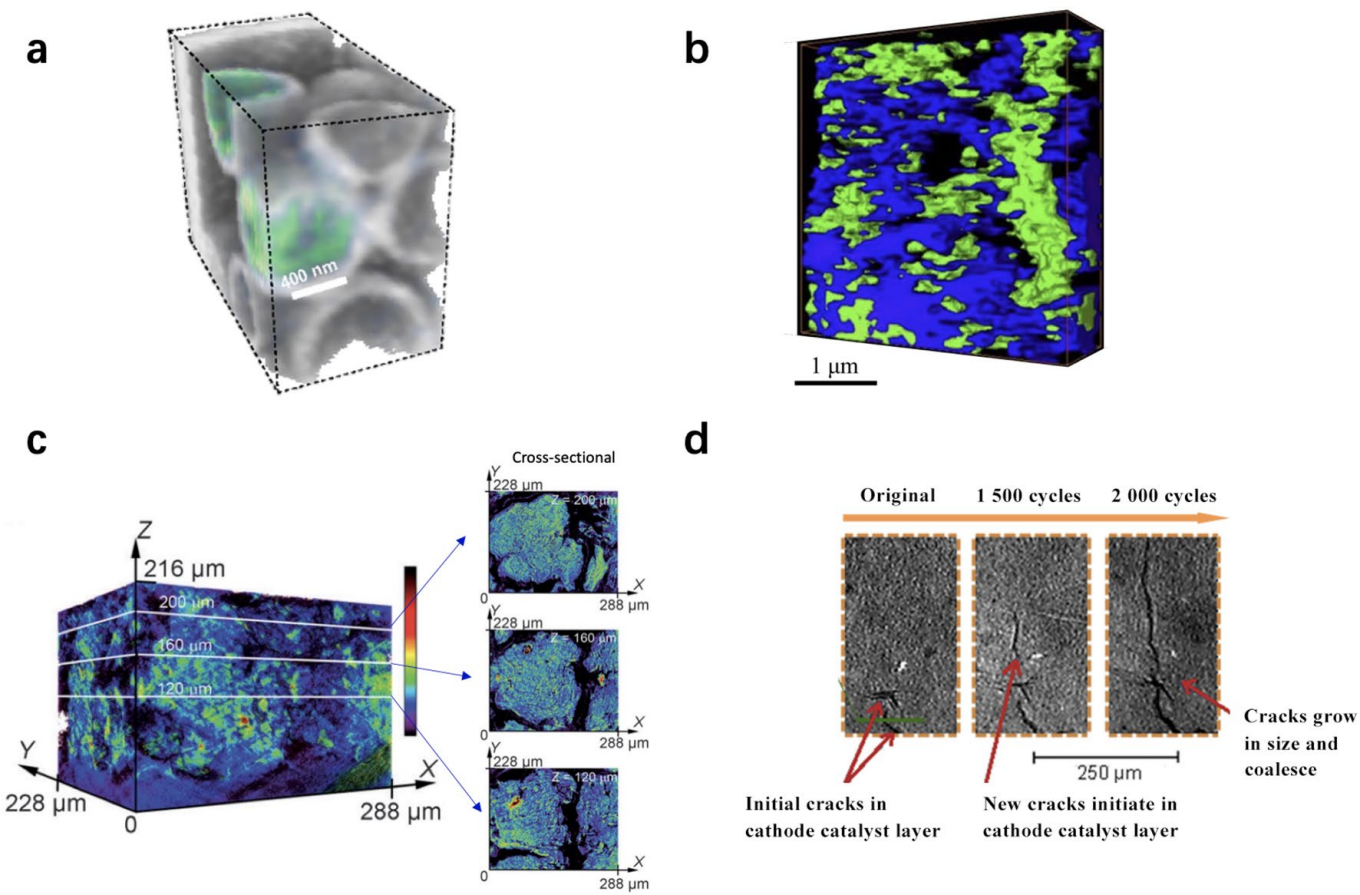
4D visualization of the catalyst layer structure. **a** Chemical composition (gray: polystyrene and glass components; blue/green: polyacrylate polyelectrolyte ionomer). Adapted with permission from Ref. [20]. Copyright © 2008, Springer Nature. **b** Chemical composition distribution of CLs [green: perfluorosulfonic acid (PFSA); blue: carbon support] visualized with a STXM by Wu et al. [98]. Adapted with permission from Ref. [98]. Copyright © 2018, Elsevier. **c** Pt distribution in cathode CLs visualized with XCL and XANES spectroscopy by Saida et al. [104] (the intensity represents the quantity of Pt catalysts). Adapted with permission from Ref. [104]. Copyright © 2012, Wiley. **d** Time-dependent degradation at the same location of cathode CLs visualized using X-ray CT by Singh et al. [105]. Adapted with permission from Ref. [105]. Copyright © 2019, Elsevier

Table 2 summarizes the typical 4D microscopy techniques that are particularly used in CL studies. Chemical composition-based 4D microscopy has been applied to CL structure to investigate the distribution and dispersion of various material components, e.g., carbon support and ionomer, using scanning transmission X-ray microscope (STXM) [20, 98]. The dispersion and distribution of chemical elements can be observed by this technique, as shown in Fig. 8a, b. Saida et al. [104] developed a 4D technique by combining the X-ray computed laminography (XCL) and X-ray absorption near-edge structure (XANES) spectroscopy to visualize the 3D structure and Pt distribution of the cathode CLs in PEM fuel cells (see Fig. 8c). This nondestructive technique can be used to analyze the chemical states of the Pt in electrodes under both fresh and degraded conditions.

**Table 2:** Summary of 4D microscopy techniques

Specimen	Technique applied	Fourth dimension	Ref.
Carbon support + ionomer	STXM	Chemical composition	[98]
Polystyrene microspheres + polyacrylate polyelectrolyte ionomer	STXM	Chemical composition	[20]
Pt/C	XCL + XANES spectroscopy	Chemical composition	[104]
Electrode + membrane	X-ray CT	Time	[105]
Pt/C + ionomer	X-ray CT	Time	[106]
Gel phantoms	Backscattered ultrasound	Temperature	[99]

The time-dependent structural degradation of the CLs under actual cell operation is of significant interest in fuel cell studies, and X-ray CT provided a promising technical pathway to monitor the interior of the fuel cell without damaging its original structure. Singh et al. [105] used X-ray CT to visualize the growth of in situ cracks in cathode CLs at the same location after a few thousand cycles of accelerated stress tests, as shown in Fig. 8d. Similarly, White et al. [106] used a micro-X-ray CT to investigate the CL thinning and crack growth under accelerated stress tests, which provided unique insights on the compactness of pore structure and electrode failure mechanism during fuel cell operation. The temperature distribution in gel phantoms was studied in [99] by using thermocouples and ultrasound imaging techniques, and 2 °C isosurfaces in gel phantoms at 25, 50, and 75 s after heating commenced can be determined by backscattered ultrasound. This technique may be potentially used for CLs as a noninvasive tool for real-time temperature variation, which requires careful design and validation for thin CLs.

### Structure Characterization

**Fig. 9:**
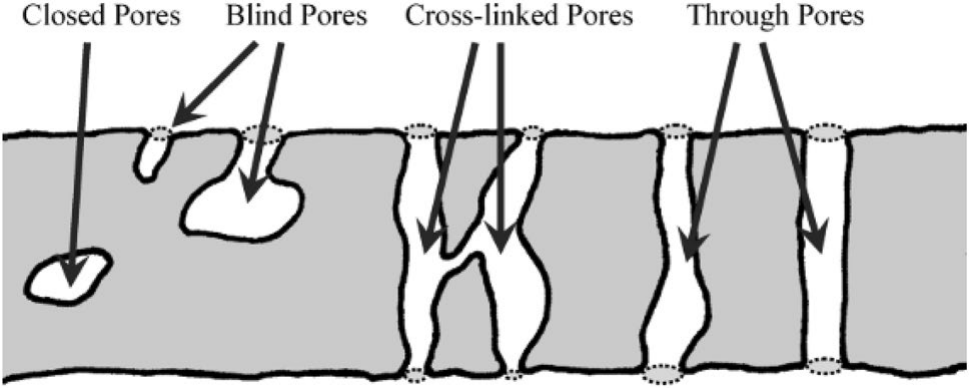
Schematic of different types of pores. Reprinted with permission from Ref. [107]. Copyright © 2006, John Wiley and Sons

The multi-scale structure of the CLs can be qualitatively visualized by various microscopy techniques; however, the quantitative analysis of the CL structure is essential to analyzing the transport, electrochemical, and degradation phenomena in PEM fuel cells. Many transport and electrochemical coefficients, e.g., effective diffusion coefficient, permeability, thermal and electrical conductivity, and capillary pressure, are a strong function of structural properties, such as porosity, tortuosity, and PSD. However, since the structure of the CLs is essentially random, irregular, and inhomogeneous, which contains closed, blind, cross-linked, and through pores (see Fig. 9), it is important to understand the key structural parameters of CLs, which are usually determined by various experimental techniques.

In this section, the most commonly used experimental methods for pore structure and solid structure of the CLs are reviewed. The experimental methods for characterizing pore structure include the method of standard porosimetry (MSP), the method of mercury porosimetry (MMP), Brunauer–Emmett–Teller (BET), densometer (based on Archimedes principles), and other methods, and the experimental methods for solid structure characterization include X-ray diffraction (XRD), electron diffraction (ED), Raman spectroscopy, thermogravimetric analysis (TGA), X-ray photoelectron spectroscopy (XPS), energy-dispersive X-ray spectroscopy (EDX), and other techniques.

#### Experimental Methods for Pore Structure Characterization

The pore structure of CLs is highly inhomogeneous and irregular, even though many studies ideally treat the pores in the shapes of cylinders, spheres, slits, and cavities [108]. The critical pore structure-related parameters, including PSD, porosity, mean pore size, and surface area, which can be experimentally determined, are all vital to understanding the transport and electrochemical phenomena in CLs.

The pores in the CLs are conventionally assumed as cylinders of different sizes when PSD is studied according to the International Union of Pure and Applied Chemistry (IUPAC) [108]. The PSD represents the distribution of pore sizes in a porous specimen [109], while the porosity is the volumetric ratio of the pores to the bulk specimen. The void volume of the specimen can be determined by the MSP [42], MMP [110], BET [108], or densometer [111], while the total volume of the specimen depends on the exterior geometry and the thickness of the CL specimen. Traditional CL thickness is determined by a micrometer, which is suitable for the thickness of more than 10 μm. To improve measurement uncertainty, a few layers of CL samples are often stacked together with slight compression [4, 112]. With the current trend to fabricate ultra-thin CLs, the micrometer may not be capable to detect such a layer thinner than 1 μm, and stacking too many thin layers may bring errors from imperfect contact between layers or excessive compression when a micrometer is applied. Therefore, SEM microscope is also used to measure the CL thickness [112]. However, the thickness of the CLs may not be uniform, especially for those with ultra-low catalyst loading, making it challenging to determine the nominal thickness. The uniformity of the CL thickness should be carefully taken into account when calculating the porosity and other effective properties of CLs. In addition, pore surface area is also an important structural parameter for a porous medium. The value of the surface area is dependent on not only the nature of the porous media but also the measurement methods employed. For instance, the measured value of the surface area can be significantly varied due to the different sizes of “ruler” (i.e., different probing liquid or gas molecules) [113]. Therefore, the comparison of surface area for various specimens should be conducted by using identical methods under the same assumptions. Typical experimental methods used for surface area measurements include BET [113], MSP [42], and MMP [114]. The mean pore size is an artificial indicator representing the mean size of channels in CLs for reactant and water transport, which depends on the pore surface area and volume, while it has different expressions with different assumptions of equivalent pore shapes (e.g., cylindrical or spherical) [115, 116].

The CLs are traditionally composed of hydrophobic and hydrophilic materials (e.g., ionomer and Pt/C, respectively), and it is important to understand the hydrophobic and hydrophilic pore structure, which is important for the CLs’ capability to repelling excess liquid water. Li et al. [117] added hydrophobic dimethyl silicone oil to the traditional cathode to enhance the hydrophobicity of the CLs, and their results demonstrated that this addition can significantly prevent the water flooding at high current densities. However, the mechanism of the improvement is still under investigation due to the lack of direct experimental evidence. The direct measurement of hydrophobicity of pores is challenging as the wetting angles are difficult to measure if the CLs cannot be prepared to form a smooth surface with uniform local materials, composition and pore size distribution. Volfkovich and Bagotzky [118] analyzed the hydrophobicity of various pores in fuel cell electrodes using MSP and found that the hydrophobicity of the pore structure can be affected by the local materials, composition, as well as local pore sizes. However, it is difficult to further verify their statistical analysis due to the lack of other experimental techniques. Yu et al. [119] applied ESEM techniques to study the time-dependent microscale hydrophobicity and hydrophilicity of the CL structure; however, the wettability is mostly measured on and near the CL surfaces but not in the interior pores. Therefore, the hydrophobicity of the CLs is not discussed further in this section, and the measurement of wettability is detailed in Sect. 3.4.

In this section, commonly employed experimental methods for pore structure characterization are reviewed, including the MSP, MMP, BET, densometer, and many other techniques.

1. *Method of standard porosimetry*
  The MSP, established based on capillary equilibrium, is one of the most commonly employed methods to measure the PSD of CLs due to its nondestructive characteristics and capability of measuring PSD under room conditions [53, 118, 120–123]. The principles of MSP are shown in Fig. 10. Based on the capillary equilibrium, the standard and test specimens, closely contacted with each other in liquid (e.g., octane and water) for a sufficiently long time, have the identical capillary pressure.

**Fig. 10:**
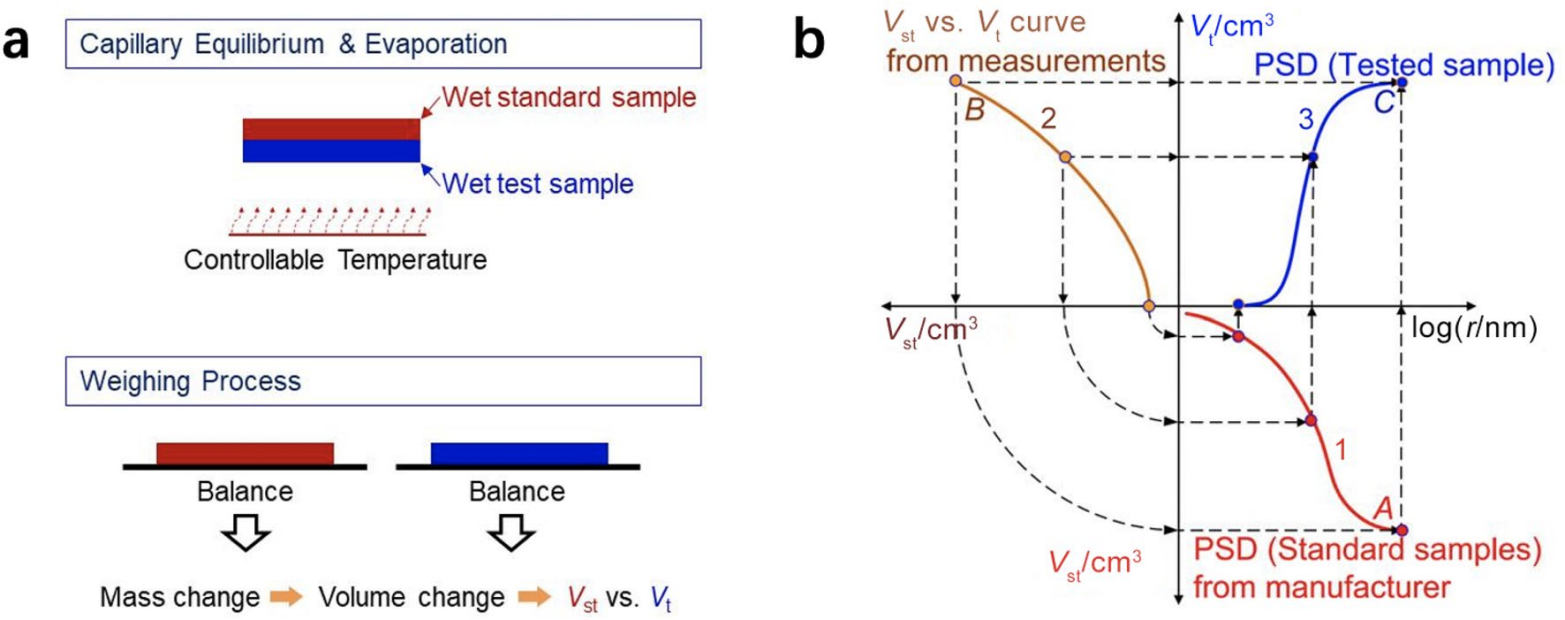
**a** Experimental procedures and **b** principles of the method of standard porosimetry. Curve 1 denotes the PSD of the standard sample from the manufacturer. Curve 2 is the measured pore volume of the standard (*V*_st_) versus test (*V*_t_) samples. Curve 3 is the measured PSD of the test sample. Adapted with permission from Ref. [118]. Copyright © 1994, Elsevier
  The total pore volume (*V*_p_) of the test specimen in [m^3^] can be calculated from the mass change between its saturated and dry states:
  1 $$ V_{{\text{p}}} = \frac{{m_{{{\text{sat}}}} {-} m_{{{\text{dry}}}} }}{\rho } $$
  where *m*_sat_ is the weight of liquid saturated specimens in [kg], *m*_dry_ is the weight of dry specimens in [kg], and *ρ* is the density of probing liquid in [kg m^−3^].
  The bulk volume (*V*_b_) of the test specimen in [m^3^] can be calculated from its geometric dimensions:
  2 $$ V_{{\text{b}}} = A\delta $$
  where *A* is the cross-sectional area in [m^2^] from the top view of the CL specimen, and *δ* is the thickness of the CLs in [m]. Therefore, the porosity (*ε*) can be calculated as follows.
  3 $$ \varepsilon = \frac{{V_{{\text{p}}} }}{{V_{{\text{b}}} }} $$
  The pore surface area can be derived from the cumulative PSD curve assuming cylinder-shaped pores via the following equation [53, 123, 124]:
  4 $$ S_{{\text{p}}} = 2\int_{{r_{{{\text{min}}}} }}^{{r_{{{\text{max}}}} }} {\frac{1}{r}\frac{{{\text{d}}V_{{\text{t}}} }}{{{\text{d}}r}}{\text{d}}r} $$
  where *S*_p_ is the pore surface area in [m^2^], and *r* is the radius of cylindrical pores in [m].
  The mean pore size (*r*_mean_) of cylindrical pores in [m] can be estimated as follows [115].
  5 $$ r_{{{\text{mean}}}} = \frac{{4V_{{\text{p}}} }}{{S_{{\text{p}}} }} $$
  Zhao et al. [42] tested the pore structure of catalyzed electrodes fabricated using CCM and CCS techniques with different Pt loadings of 0.1–0.4 mg cm^−2^ using MSP. The electrode specimens are disk-shaped with a diameter of 2.3 cm. The MSP measurements are conducted by (1) removing air and moisture from test samples, (2) weighing samples before and after immersing samples in octane, (3) clamping test samples between two standards, (4) recording the mass change after the new equilibrium is achieved, and (5) plotting the PSD curve by comparing test samples with the standards. The experimental results indicate that the electrodes prepared by CCS methods are thinner with higher porosity, less surface area, lower permeation and diffusion resistance, and worse performance, in comparison with that prepared by CCM methods. The significant performance drop is caused by the loss of catalyst particles, deposited in the interior GDL structures. The penetration of catalyst particles is visualized by Jhong et al. [19].
2. *Method of mercury porosimetry*
  MMP, a.k.a., mercury intrusion porosimetry (MIP), is developed based on a modified Young–Laplace equation (or Washburn equation) with the assumption of cylinder-shaped pores, and the capillary pressure can be calculated based on surface tension, pore radius, and contact angle:
  6 $$ \Delta p = \sigma \left( {\frac{1}{{r_{1} }} + \frac{1}{{r_{2} }}} \right) = \frac{{2\sigma {\text{cos }}\theta }}{{r_{{\text{p}}} }} $$
  where ∆*p* is the pressure drop in [Pa] across the liquid–gas interface, *r*_1_ and *r*_2_ are the interfacial curvatures in [m], and *r*_p_ is the radius (or half pore size) of the associated pores in [m].
  To obtain the pore–size–volume relation of the porous specimen, the size and volume of pores should be measured simultaneously. The size of pores can be estimated from the pressure difference according to Eq. (6) with known surface tension and contact angle. The pressure drop is one of the most important variables that determine the measurement uncertainties, which may cover five orders of magnitudes [107]. Due to the multi-scale nature of the pore sizes in CLs, a wide range of pressure is needed to be applied during the measurement. The wide range of pressure may require more than one single pressure transducer (see Fig. 11) to ensure the measurement accuracy and sufficient resolution over the entire measurement range. However, particular attention should be paid to measurement errors at the switchover points between different transducers. The surface tension of mercury can be experimentally determined on different surfaces, and in practice, a constant value of 0.485 N m^−1^ at 25 °C is widely employed to determine PSD. The effects of temperature and pressure on the value of mercury surface tension on solid surfaces can affect the results to some extent; however, corrections are generally not applied to the data interpretation, where the uncertainty from the contact angle is deemed as minimal [107]. The contact angle can be measured from a drop of mercury on the specimen surface by either fitting the shape or measuring the height of mercury drops. It should be pointed out that the MMP is performed in the air or oil environment, where the values of the contact angle on the specimen and surface tension of mercury should be adjusted accordingly.
  The volume of pores with a particular size can be determined by measuring the capacitance between a mercury column in a glass capillary and a metal shield covering the capillary. The measurement uncertainty may result from bad electrical contacts, contaminations, or glass chips [107]. The pore volume can also be estimated from a syringe which is used to pressurize the mercury into the pores under given pressures (see Fig. 11). By increasing the applied pressure incrementally, a particular volume of mercury is continued to be injected into CL pores, which can help establish the pore–size–volume relation. It should be noted that it is necessary to regularly calibrate the MMP instruments against “standard” samples, which contain a variety of well-defined pores [23, 125, 126].
  Rootare and Prenzlow [114] established an equation to calculate the surface area based on MMP:
  7 $$ S_{{\text{p}}} = - \frac{1}{{\sigma_{{{\text{Hg{-}air}}}} {\text{cos }}\theta }}\int_{0}^{V} {p{\text{d}}V} { } $$
  where *p* is the external pressure in [Pa].
3. *Method of Brunauer–Emmett–Teller*
  **Fig. 11:**
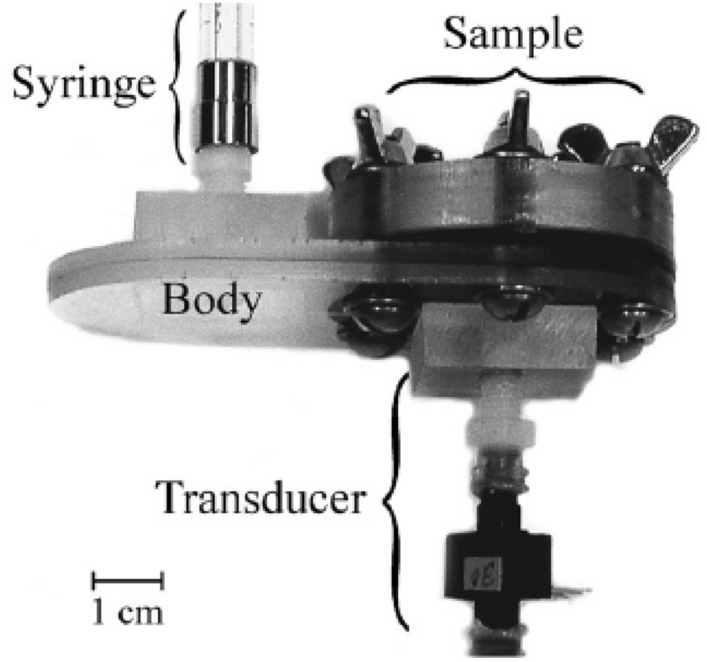
Schematic of the method of mercury porosimetry. Reprinted with permission from Ref. [127]. Copyright © 2007, Elsevier

The interior surface area of the porous media is broadly measured by BET method, established based on the physical adsorption of gas molecules on pore surface. Nitrogen is the most frequently employed probing substance for BET measurement, although argon, carbon dioxide, and oxygen can also be employed [128]. For the nitrogen-based BET method, the surface area of a porous medium can be calculated by analyzing the nitrogen adsorption at the temperature of 77 K under various relative pressure. The number of molecules adsorbed on pore surface can be calculated from the physisorption isotherm based on the BET theory [108] as follows:
  8 $$ \frac{p}{{n\left( {p_{0} - p} \right)}} = \frac{1}{{n_{{\text{m}}} C}} + \frac{C - 1}{{n_{{\text{m}}} C}}\frac{p}{{p_{0} }} $$
  where *n* denotes the quantity of adsorbed substances in [mol] under the relative pressure of *p*/*p*_0_, *n*_m_ is the monolayer capacity in [mol], and *C* is a coefficient calculated from the shape of the isotherm curve. According to Eq. (8), a linear relation between *p*/[*n*(*p*_0_ *− p*)] and *p*/*p*_0_ can be established from a BET plot (see Fig. 12 for example). The slope of the BET plot is equal to (*C* − 1)/(*n*_m_*C*), and the intercept value can be expressed as 1/(*n*_m_*C*), and thus the monolayer capacity, *n*_m_, can be calculated. The BET surface area (*S*_BET_) in [nm^2^] can be calculated as follows:
  9 $$ S_{{{\text{BET}}}} = n_{{\text{m}}} N_{{\text{A}}} A_{{{\text{N}}_{2} }} $$
  where *N*_A_ is the Avogadro constant (6.022 × 10^23^ mol^−1^), and $A_{{{\text{N}}_{2} }}$ is the equivalent cross-sectional area of a single probing molecule ($A_{{{\text{N}}_{2} }}$ = 0.162 nm^2^ for close-packed nitrogen at 77 K) [108, 128, 129].

**Fig. 12:**
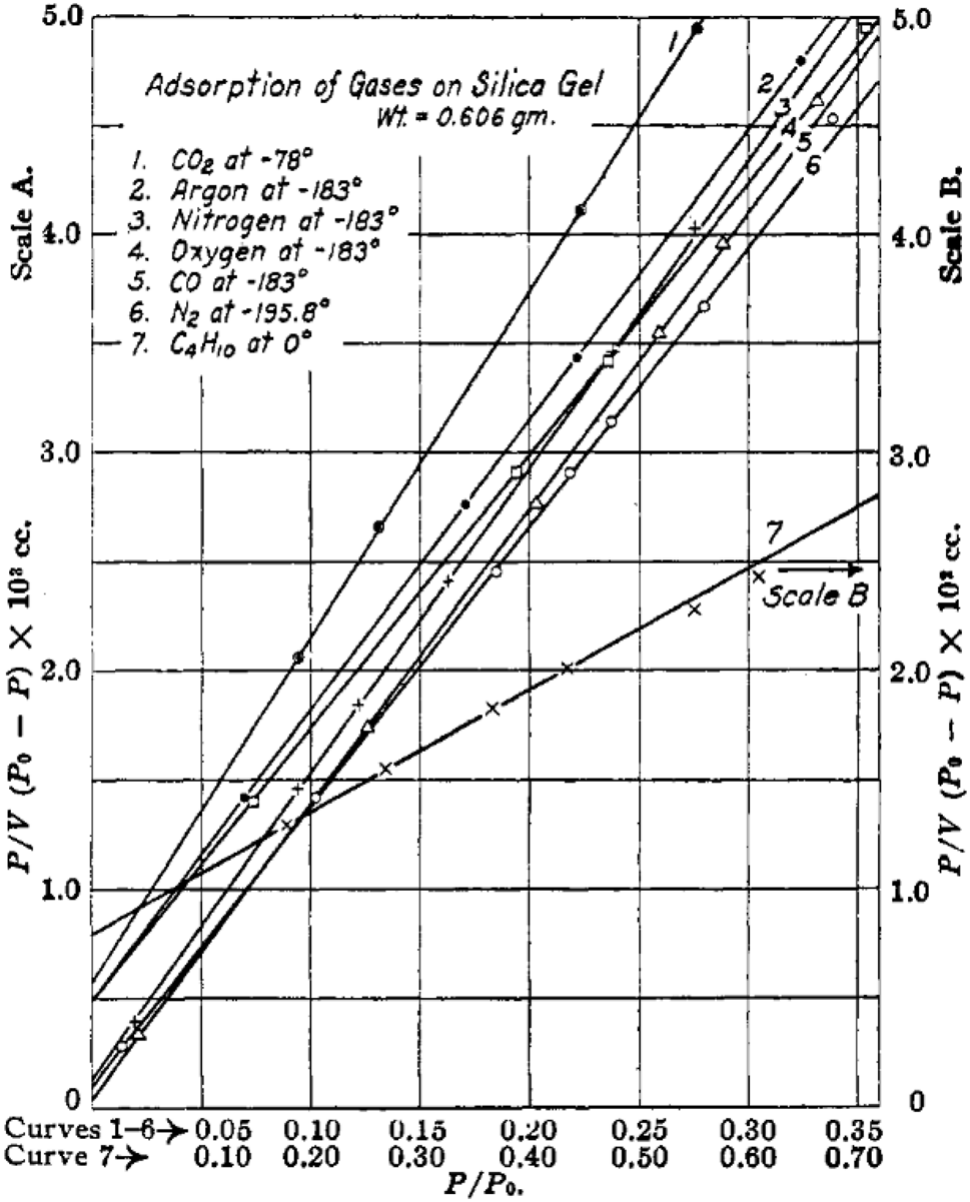
BET isotherm curves based on different substances. Reprinted with permission from Ref. [128]. Copyright © 1938, American Chemical Society
  Many studies suggested good correlations between surface areas measured by different experimental methods [114, 130]. Zhao et al. [113] measured the surface area of the fuel cell electrode (including a CL and a GDL) using MSP and BET methods, respectively. The experimental results identified a significant difference in pore surface area determined by MSP and BET methods, and the fractal dimension theory suggests that the difference results from the different sizes of “rulers”, i.e., the different probing molecules (nitrogen for BET, and octane for MSP) of various molecular sizes, employed in the respective method. The experimental data suggest that the pore surface area is very sensitive to the minimum pore sizes under investigation, and the pores with small sizes dominate surface area of a specific porous medium. It should also be noted that the actual shape and dimension of pores can be very different from the ideal scenarios; therefore, the interpretation of experimental data collected by various porosimetry methods should be carefully performed [107].
4. *Method of densometer (Archimedes principle)*
  The method of densometer based on the Archimedes principle (or buoyancy-based porosity measurement) is investigated in various studies [111, 131], which enables a direct measurement of a single thin layer, as shown in Fig. 13.

**Fig. 13:**
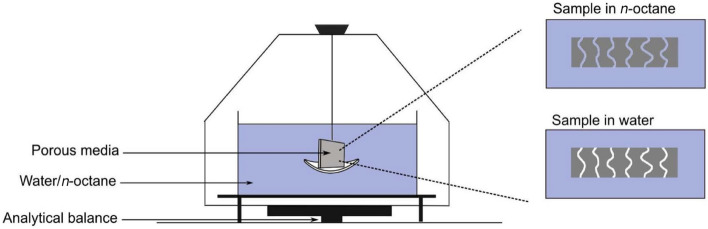
Schematic of the experimental setup for porosity measurement based on the Archimedes principle by Shukla et al. Reprinted with permission from Ref. [111]. Copyright © 2019, the Electrochemical Society
  The typical experimental setup includes a high-precision balance, working liquid, and a wireframe. The specimen is prepared in a specific shape such that the bulk volume can be calculated from the exterior geometry. The dry specimen is first weighed in the air using the balance, subsequently submerged in the working liquid (e.g., octane, water, or silicon oil) in a vacuum chamber to remove any existing air bubbles from the pores, then carefully placed in the liquid with the help of the wireframe, and finally measured the weight change after the sample is submerged in the liquid. Based on the Archimedes principle, the volume (*V*_s_) of the solid components in [m^3^] can be calculated as follows:
  10 $$ V_{{\text{s}}} = \frac{{m_{{{\text{s}},{\text{air}}}} - m_{{{\text{s}},{\text{l}}}} }}{{\rho_{{\text{l}}} - \rho_{{{\text{air}}}} }} $$
  where *ρ*_l_ is the density of the liquid (can be experimentally determined or obtained from the manufacturer) in [kg m^−3^], *ρ*_air_ is the air density in [kg m^−3^], and *m*_s,air_ and *m*_s,l_ are the weights of solids measured in air and liquid in [kg], respectively.
  The porosity of the specimen can be determined as follows.
  11 $$ \varepsilon = \frac{{V_{{\text{p}}} }}{{V_{{\text{b}}} }} = 1 - \frac{{V_{{\text{s}}} }}{{V_{{\text{b}}} }} $$
  The Archimedes method is advantageous for the direct measurement of a thin layer specimen, which is of potential to minimize the measurement errors with good repeatability [131]. However, the uncertainties from the high-precision balance, the size and hydrophobicity of the specimens, the uniformity and errors of the thickness, and the potential air bubbles existing in the specimen placed in the liquid should be carefully controlled.
5. *Comparison of different pore structure characterization techniques*
  Many other methods can be employed to investigate the pore structure of porous media, especially the PSD, and these methods can be categorized into fluid- and radiation-based methods, as shown in Fig. 14. The fluid-based methods include MSP [53, 118, 120–123], MMP [132, 133], gas adsorption [134], capillary condensation [135], and displacement method [136], while the radiation-based methods include small-angle X-ray scattering [137], optical microscopy [138], SEM [138, 139], TEM [138, 140], and AFM [138]. However, particular attention should be paid to the certain limitations of each technique for measuring CLs in PEM fuel cells. For example, the accuracy of the MSP relies on the PSD of the standard samples, which are given by the manufacturer. The accuracy of standard PSD and its effect on the experimental results of the test sample remains unclear, although the MSP enables the nondestructive measurements of CL structure under room conditions over a broad range of pore sizes (typically from 0.3 nm to 300 μm). However, the MMP may be detrimental to the delicate CL microstructure as a high external pressure is required to inject mercury into the pores of CLs, which can distort the intrinsic CL structure [118, 122]. The gas adsorption, capillary condensation, and small-angle X-ray scattering are suitable for only micro- and meso-pores (< 50 nm), while the displacement method is commonly used for macro pores (> 10 μm) [122, 134–137, 141], as shown in Fig. 14. The microscopic images are also widely used to qualitatively analyze the shape and size of pores (mostly near the specimen surface), and quantitative analysis of the PSD depends on image-processing algorithms [113].

**Fig. 14:**
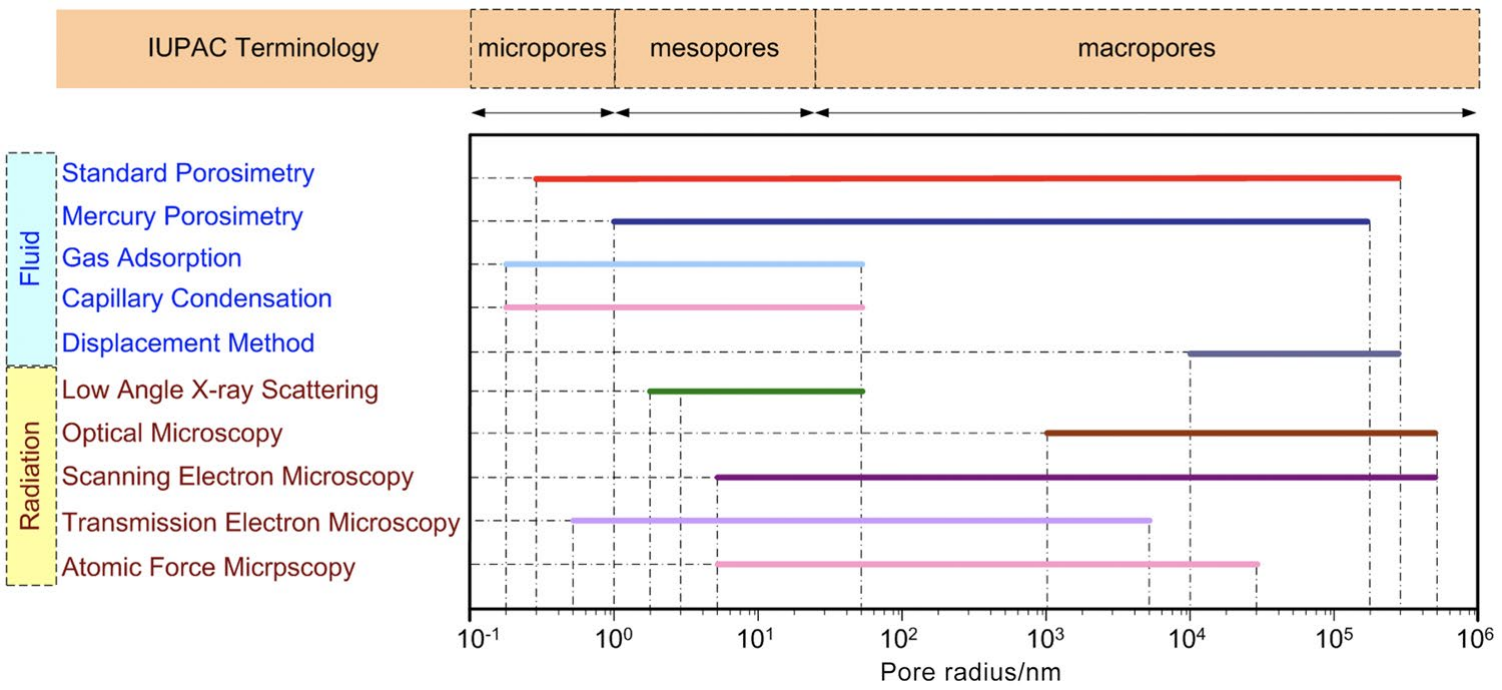
Comparison of the pore size ranges that different methods can be used to determine the pore structure of porous media. Adapted with permission from Ref. [113]. Copyright © 2019, John Wiley and Sons

#### Experimental Methods for Solid Structure Characterization

When a CL is prepared, advanced composition and phase analysis techniques are often performed to ensure the manufacturing consistency, to check fabrication procedures, and to inspect impurity species. The frequently used composition and phase analysis techniques include XRD, ED, Raman spectroscopy, TGA, XPS, EDX, and many other techniques. The principles and applications of each technique are summarized in Table 3.

XRD is a nondestructive technique to investigate the solid structure of CLs by analyzing the resultant diffraction pattern of X-ray photons after interacting with and being scattered by electrons surrounding the atoms [142]. XRD has been applied to analyze the atomic composition [9], oxidation states of catalysts [143], size and shape of catalyst nanoparticles [126, 144, 145], crystal structure of carbon supports, non-platinum catalysts [13], and PFSA ionomer [146, 147], and pore sizes in well-ordered materials [109, 148]. Electron diffraction is established based on the analysis of elastically scattered electrons, which can be used to analyze the crystal structure of catalyst or carbon support, e.g., single-walled carbon nanotubes [149]. Raman spectroscopy is a nondestructive technique based on the inelastic scattering of monochromatic light, which is widely used to analyze the structural changes in carbon materials during accelerated stress test, including carbon supports or nonmetal catalysts [150]. TGA is a destructive method that analyzes the mass changes as temperature rises [151]. TGA is widely used in CL analysis, including the thermal stability of the catalyst [152] and membrane [153] materials, and the measurement of Pt content in Pt/C [154]. XPS is a common technique employed for material analysis based on X-ray electrons, which is widely used to characterize the surface elemental composition [3] for various materials, including inorganic compounds, metal alloys [155], Nafion membrane [155], and Pt and oxidized Pt species [156]. EDX is another technique widely used for material analysis by detecting X-rays emitted from a material surface after interacting with an electron beam. EDX is widely used to identify and quantify the elements [157], to analyze the distributions of elements coupled with SEM or TEM [158], and to characterize nanostructure, e.g., core–shell and alloy nature [159].

**Table 3:** Comparison of typical experimental methods for catalyst layer solid structure characterization

Method	Probing species/principle	Remark	Application
XRD	Diffraction of X-ray photons	Nondestructive analysis technique	Atomic composition [9] Oxidation states of catalysts [143] Size and shape of Pt [126, 144, 145] Crystal structure of carbon supports, non-platinum catalysts [13], and PFSA ionomer [146, 147] Pore sizes in well-ordered materials [109, 148]
ED	Elastically scattered electrons	Usually coupled with SEM or TEM Stronger reflection due to shorter wavelength than X-rays	Crystal structure Single-walled carbon nanotubes [149]
Raman spectroscopy	Inelastic scattering of monochromatic light in visible, near-infrared, or near ultraviolet range	Nondestructive analysis technique	Structure of carbon [150]
TGA	Mass change over time as temperature changes	Destructive analysis technique	Decomposition patterns Adsorbed moisture content Relative organic composition Thermal stability of catalysts [152] and membranes [153] Identify Pt content in Pt/C [154]
XPS	X-ray electrons	Ultra-high vacuum needed to minimize errors	Surface elemental composition, empirical formula, chemical and electron states of the elements existed in a material [3] Suitable for inorganic compounds, metal alloys, semiconductors, polymers, and other materials [155] Decomposition of Nafion materials [155] Pt and oxidized Pt species [156]
EDX	Emitted X-rays	Commonly integrated with SEM and TEM	Identify and quantify elements [157] Maps of distributions of elements with SEM or TEM [158] Nanostructures like core–shell and alloy nature [159]

### Summary

The microstructure of CLs, formed during the fabrication process, can be affected by many factors, including materials, composition, fabrication methods, conditions and procedures. The PTFE-bonded CLs are durable due to the extremely high Pt loading applied; however, the high cost resulted from the large amount of noble Pt catalyst unfavored this method in industrial application. Vice versa, the ultra-low-Pt-loading CLs prepared by the plasma sputtering method, ion-beam-assisted deposition, or atomic layer deposition can considerably decrease the material cost; however, these methods remain impractical for large-volume manufacturing due to technical challenges in complex fabrication apparatus and unconfirmed long-term performance [31]. The ionomer-bounded method (a.k.a. the thin-film method) demonstrates a good balance between durability and cost, which can be further optimized by improving the CL microstructure. The multi-scale structure of CLs can be visualized by different microscopy techniques, including optical microscopy, SEM, TEM, and AFM, which are suitable to identify the morphology and topology of the CL surface with different spatial resolution. The interior structure can be visualized by FIB/SEM and 3D X-ray CT methods. Advanced 4D microscopy techniques have been also adopted for fuel cell studies to investigate the fourth “dimension”, e.g., chemical composition, temperature, time, and other information. Quantitative characterization of the multi-scale CL pore structure includes porosity, PSD, surface area, mean pore size, tortuosity, and other parameters. The pore structure can be characterized by the MSP, MMP, BET, and densometer, and other techniques. The solid structure can be studied by XRD, electron diffraction, Raman spectroscopy, TGA, XPS, EDS, and other methods.

## Physicochemical Properties of Catalyst Layers

The physicochemical properties, which significantly affect the transport of reactants, water, and heat in the CLs, are determined by the compositional ingredients and multi-scale structure. The performance and durability of CLs can also be affected by various transport and mechanical properties, such as the effective diffusion coefficient, permeability, capillary pressure, contact angle, effective thermal conductivity, and Young’s modulus [123, 160, 161]. Unfortunately, the experimental data of these effective properties are very limited for the CLs, due to the difficulties in measuring a thin layer of porous media. Therefore, various experimental techniques specifically designed and potentially applied for the CLs have been comprehensively reviewed in this section. The physicochemical properties are strongly structure-dependent, and the relation between these properties and structural parameters is scrutinized in this section.

### Effective Diffusion Coefficient

#### Fick’s Law of Diffusion

Diffusion, one of the key mass transfer mechanisms in fuel cells, is defined as the net movement of molecules as a result of random molecular motion, which can be caused by a gradient of concentration, temperature, pressure, or external force [160, 162, 163]. The rate of diffusion is governed by Fick’s law of diffusion [164].

12 $$ J_{{\text{m}}} = - D\frac{\partial c}{{\partial x}} $$

where *J*_m_ represents the mass flux caused by diffusion in [kg m^−2^ s^−1^], *c* denotes the concentration in [kg m^−3^], *x* is the diffusion distance in [m], and *D* denotes the diffusion coefficient in [m^2^ s^−1^].

In the open spaces, the diffusion is driven by the collisions between molecules without the interference by any object. The diffusion coefficient is known as the bulk diffusion coefficient, which is governed by not only the gradients of temperature, pressure, and concentration but also the nature of the diffusion substances. In porous media, e.g., the CLs of PEM fuel cells, the reactant gas molecules can collide with a solid CL surface, which slows down the diffusion rates. Therefore, the Fick’s law needs to be modified for the diffusion in porous media, where an effective diffusion coefficient is used to replace the bulk diffusion coefficient.

13 $$ J_{{{\text{m}},i}} = - D_{{{\text{eff}}}} \frac{{\partial c_{{{i}}} }}{\partial x} $$

where the subscripts, *i* and eff, denote species *i* and effective properties, respectively. The diffusion coefficient in porous media is lower than that in the bulk region as the collision with solid surfaces makes the transport of gas species more difficult.

It should be noted that with the current trends to fabricate CLs with ultra-low loadings much less than 0.1 mg cm^−2^, the thickness of the CLs can be only a few nanometers. Therefore, the reactant transport resistance, especially for oxygen at the cathodes, through pores can be reduced, while that through the thin films of ionomer covered on the surface of catalyst particles becomes dominant. Based on the assumptions that the catalyst surface is covered by a thin ionomer layer in the interior structure of CLs, the concentration of the dissolved reactants at the ionomer–gas interfaces can be calculated by Henry’s law [81, 165].

14 $$ c_{i} = \frac{{p_{i} }}{{H_{i} }} $$

where *c* is the concentration of gas species in [kmol m^−3^] in the ionomer phase, *p* is the partial pressure of gas species *i* (i.e., O_2_ or H_2_) in [Pa] in the gas phase, and *H* is the Henry’s constant in [Pa m^3^ kmol^−1^]. The dissolved gas species is transported mainly via the diffusion through the ionomer–gas interface to the catalyst surface which is covered by a thin layer of ionomer. In many numerical studies, Henry’s law and Fick’s law of diffusion are combined to model the mass transport of the reactants [81, 165]. However, the experimental data on the Henry’s constant and the diffusion coefficients are rarely reported in the literature.

#### Experimental Methods for Effective Diffusion Coefficient

**Fig. 15:**
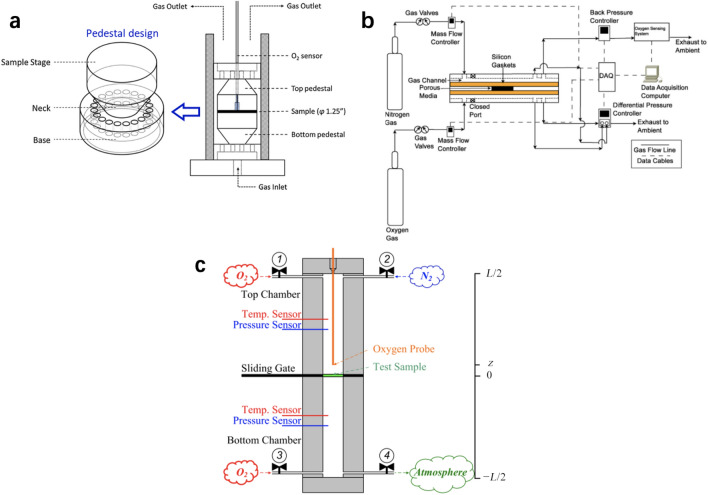
Schematic of different diffusivity apparatus. **a** Radial diffusivity apparatus by Kim and Gostick [166]. Adapted with permission from Ref. [166]. Copyright © 2019, Elsevier. **b** Diffusion bridge apparatus by Mangal et al. [167]. Reprinted with permission from Ref. [167]. Copyright © 2015, Elsevier. **c** Modified Loschmidt cell by Zhao et al. [123]. Reprinted with permission from Ref. [123]. Copyright © 2018, Elsevier

Many experimental methods have been developed to measure the effective diffusion coefficient of porous media in PEM fuel cells based on the modified Fick’s law of diffusion. Kim and Gostick [166] developed a radial diffusivity apparatus consisting of a pedestal, a cylinder chamber, and an oxygen sensor, as shown in Fig. 15a. The experimental apparatus is designed for the thin porous specimens based on the transient variation of oxygen concentration at the center of the specimens by fitting the analytical solution of Fick’s law in a cylindrical system filled by nitrogen–air mixture. The experimental results suggest that the broadly used Bruggeman correlation for estimating the effective diffusion coefficient of fuel cell components based on porosity is generally unsuitable for non-spherical porous materials.

Mangal et al. [167] developed a diffusion bridge apparatus to measure the through-plane diffusivity of porous media, as shown in Fig. 15b. The apparatus is operated with nitrogen and oxygen flowing across the bridge, and an oxygen sensor is used to record the oxygen concentration. Experimental data are fitted with a combined Fick’s and Darcy’s models to calculate the effective diffusion coefficient. By analyzing the oxygen flux in the advection–diffusion process, the permeability of different thin porous media can be measured.

For CLs, the major challenge to measure the through-plane effective diffusivity is that the CLs cannot stand alone, which requires a porous substrate with known effective diffusivity and thickness. By utilizing the resistance network theory, the effective diffusivity of the CLs can be derived by measuring the diffusion resistance of the substrate with and without CLs coated. Shen et al. [168] measured the effective diffusion coefficient of the CLs [30 wt% (wt% means the weight percentage) ionomer mixed with Pt/C, 0.2–0.8 mg_Pt_ cm^−2^, 6–29 μm] deposited on the surface of porous Al_2_O_3_ using a modified Loschmidt cell, and the results indicated that the effective diffusivity of the CL is (1.46 ± 0.05) × 10^−7^ m^2^ s^−1^ under room conditions [25 °C and 1 atm (1 atm = 101.325 kPa)]. Zhao et al. [123] also investigated the effective diffusivity by measuring the effective diffusivity of GDL substrate and catalyzed GDL (25 wt% ionomer mixed with Pt/C, 0.1–0.4 mg_Pt_ cm^−2^, 3–9.4 μm) with the modified Loschmidt cell as shown in Fig. 15c. The effective diffusivity is derived based on the resistance network theory [169] as follows:

15 $$ D_{{{\text{CL}}}}^{{{\text{eff}}}} = \left( {\delta_{{{\text{sub}}\_{\text{CL}}}} - \delta_{{{\text{sub}}}} } \right)\left( {\frac{{\delta_{{{\text{sub}}\_{\text{CL}}}} }}{{D_{{{\text{sub}}\_{\text{CL}}}} }} - \frac{{\delta_{{{\text{sub}}}} }}{{D_{{{\text{sub}}}} }}} \right)^{ - 1} $$

where *δ* is the thickness in [m], and the subscripts of sub, CL, and sub_CL, denote the properties of the substrate, CL, and catalyzed substrate, respectively. The experimental data suggested that the effective diffusivity of the CLs ranges within (2.8×10^−7^–4.9×10^−7^ m^2^ s^−1^ under room conditions and (3.9×10^−7^–5.1×10^−7^ m^2^ s^−1^ at 75 °C. More details about the experimental data on the effective diffusivity of CLs are presented in Table 4. It should be mentioned that the measured effective diffusivity of CLs in Ref. [123] is about 2–3 times larger than that in Ref. [168]. This discrepancy is likely due to the different composition and structures of the CL samples used for the measurement, e.g., resulted from the different catalyst types and ionomer ratios.

**Table 4:** Effective diffusivity of the catalyst layers from experimental results

Catalyst layer composition	Thickness/µm	Working fluid	Effective diffusivity/(10^−7^ m^2^ s^−1^)	Measurement condition	Ref.
25 wt% ionomer mixed with Pt/C(60%), 0.1 mg_Pt_ cm^−2^	3	N_2_–O_2_	4.9 ± 0.3	25 °C and 1 atm, substrate GDL	[123]
25 wt% ionomer mixed with Pt/C(60%), 0.2 mg_Pt_ cm^−2^	4.8	N_2_–O_2_	4.6 ± 0.1	25 °C and 1 atm, substrate GDL	[123]
25 wt% ionomer mixed with Pt/C(60%), 0.3 mg_Pt_ cm^−2^	7.6	N_2_–O_2_	4.4 ± 0.3	25 °C and 1 atm, substrate GDL	[123]
25 wt% ionomer mixed with Pt/C(60%), 0.4 mg_Pt_ cm^−2^	9.4	N_2_–O_2_	2.8 ± 0.1	25 °C and 1 atm, substrate GDL	[123]
25 wt% ionomer mixed with Pt/C(60%), 0.1 mg_Pt_ cm^−2^	3	N_2_–O_2_	5.1 ± 0.4	75 °C and 1 atm, substrate GDL	[123]
25 wt% ionomer mixed with Pt/C(60%), 0.2 mg_Pt_ cm^−2^	4.8	N_2_–O_2_	4.8 ± 0.4	75 °C and 1 atm, substrate GDL	[123]
25 wt% ionomer mixed with Pt/C(60%), 0.3 mg_Pt_ cm^−2^	7.6	N_2_–O_2_	4.5 ± 0.1	75 °C and 1 atm, substrate GDL	[123]
25 wt% ionomer mixed with Pt/C(60%), 0.4 mg_Pt_ cm^−2^	9.4	N_2_–O_2_	3.9 ± 0.1	75 °C and 1 atm, substrate GDL	[123]
30 wt% ionomer mixed with Pt/C(46%)	6	N_2_–O_2_	1.36	25 °C and 1 atm, substrate Al_2_O_3_	[168]
30 wt% ionomer mixed with Pt/C(46%)	9	N_2_–O_2_	1.67	25 °C and 1 atm, substrate Al_2_O_3_	[168]
30 wt% ionomer mixed with Pt/C(46%)	10	N_2_–O_2_	1.24	25 °C and 1 atm, substrate Al_2_O_3_	[168]
30 wt% ionomer mixed with Pt/C(46%)	12	N_2_–O_2_	1.50	25 °C and 1 atm, substrate Al_2_O_3_	[168]
30 wt% ionomer mixed with Pt/C(46%)	14	N_2_–O_2_	1.47	25 °C and 1 atm, substrate Al_2_O_3_	[168]
30 wt% ionomer mixed with Pt/C(46%)	20	N_2_–O_2_	1.62	25 °C and 1 atm, substrate Al_2_O_3_	[168]
30 wt% ionomer mixed with Pt/C(46%)	23	N_2_–O_2_	1.50	25 °C and 1 atm, substrate Al_2_O_3_	[168]
30 wt% ionomer mixed with Pt/C(46%)	29	N_2_–O_2_	1.43	25 °C and 1 atm, substrate Al_2_O_3_	[168]

**Fig. 16:**
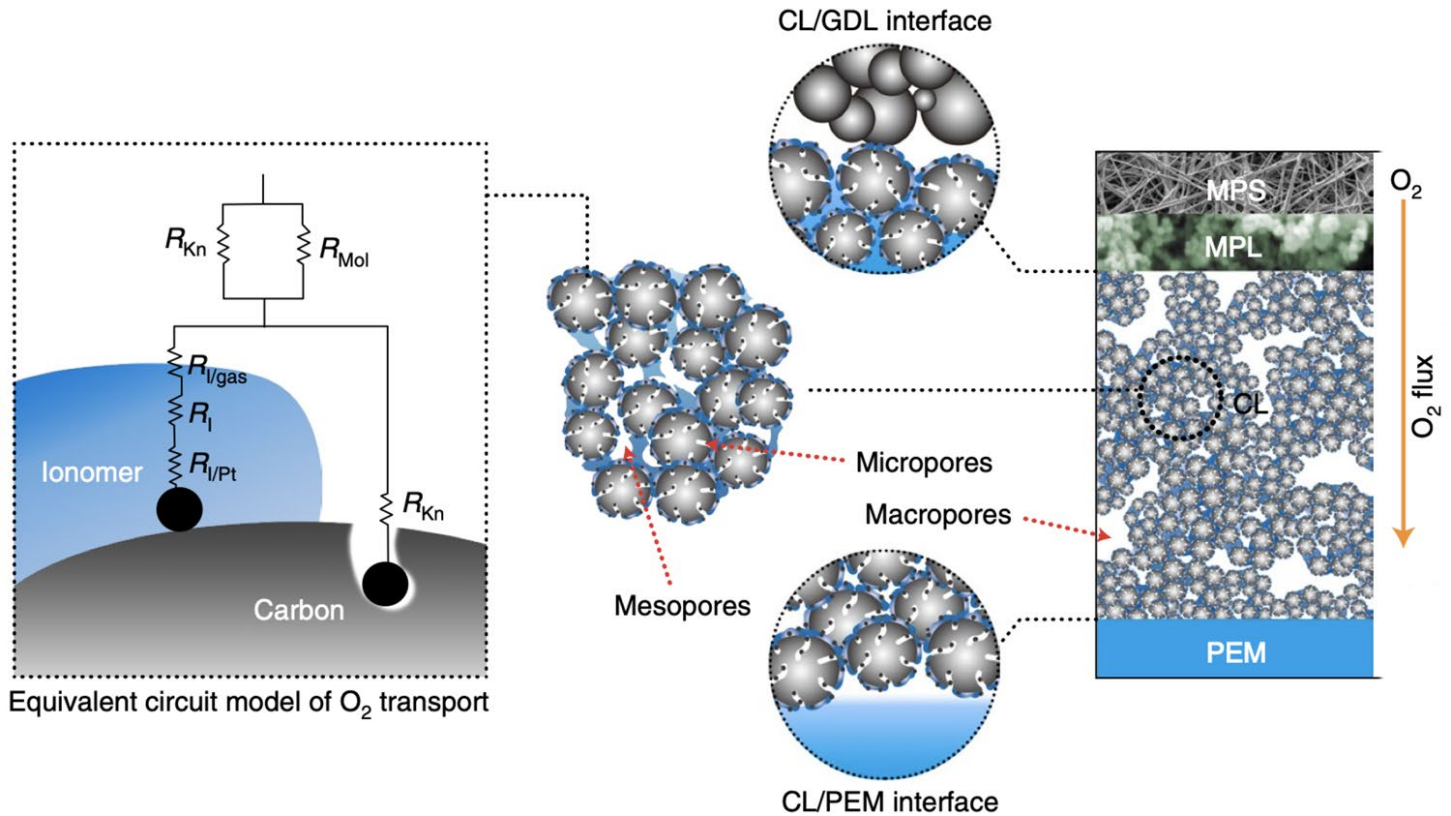
Mass transport resistance network in PEM fuel cell cathode electrodes (MPS: microporous substrate; *R*_Kn_: Knudsen diffusion resistance; *R*_Mol_: molecular diffusion resistance; *R*_I/gas_: the contact resistance between gas and ionomer; *R*_I_: the resistance through ionomer; *R*_I/Pt_: the contact resistance between ionomer and Pt catalyst). Adapted with permission from Ref. [8]. Copyright © 2021, the Author(s)

Recently, many efforts have been devoted to the understanding of the oxygen transport resistance in pores and ionomers and through the corresponding interfaces. As shown in Fig. 16, the CLs, composed of Pt/C particles, ionomer-covered agglomerates, and multi-scale pore networks, involve complicated oxygen transport pathways in the cathode structure [8]. The oxygen in pores can be Fickian or Knudsen diffusion depending on the pore sizes, and a portion of oxygen can be dissolved in ionomer, acrossing the ionomer–gas interface. The oxygen is then diffused in the ionomers from the ionomer–gas interface to the ionomer–catalyst interface, where oxygen will be adsorbed and react. Many efforts have been devoted to separating and quantifying the oxygen transport resistances in different cell components. Xue et al. [170] analyzed the EIS results performed at a high current density of 1.8 A cm^−2^ by fitting the EIS spectrums with a Warburg admittance function and found that the Nafion contents in CLs can significantly affect the effective diffusion coefficient of oxygen in CLs although oxygen transport resistances were not separated in pores, ionomers, and through interfaces. Choo et al. [171] utilized a limiting current technique to separate the contribution of GDLs and CLs to the overall oxygen transport resistances. Their experimental results suggested that the water update in the ionomer film can help reduce the oxygen transport resistance in the CLs. Nonoyama et al. [172] assumed the total oxygen transport resistance is composed of three components: pores in GDLs, pores in CLs, and ionomer film in CLs. The total resistance is quantified by measuring the limiting current density under controlled conditions ensuring no liquid water exists in CL pores, and the experimental results suggested that the ionomer film played a significant role in oxygen transport resistance at various Pt loadings under investigation. It should be mentioned that the oxygen transport resistance in the ionomer film was sometimes reported negligible, especially at high Pt loading and high temperature conditions [172]. Due to the nature of inhomogeneous coverage of ionomer, irregular shapes of catalyst surface, non-uniform oxygen distribution in pores, and uncertain local liquid water coverage in the interior CL structure, theoretical analysis and optimization of oxygen transport resistances through the CL structure still need more in-depth investigation and better understanding.

#### Empirical Models for Effective Diffusion Coefficient

Three major diffusion mechanisms exist in the porous media: surface diffusion, bulk (a.k.a. Fickian or ordinary) diffusion, and Knudsen diffusion [173]. Surface diffusion refers to the molecular movement on solid surfaces, bulk diffusion is molecular motion driven by the collisions between adjacent molecules, while Knudsen diffusion is mainly caused by the collisions between solid surface and molecules if the pore size is less than the mean free path length of the molecules [173, 174]. Taking both Fickian diffusion and Knudsen diffusion in pore networks with a broad range of pore sizes into account, the effective diffusion coefficient in a porous material can be affected by the porosity and tortuosity (defined as the ratio of the tortuous length to the straight length) [175]. The effective diffusion coefficient of a porous specimen can be empirically calculated as follows [175]:

16 $$ D_{{{\text{eff}}}} = \frac{{\varepsilon D_{{\text{b}}} }}{\tau } $$

where *ε* is the porosity, and *τ* is the tortuosity. The tortuosity of unconsolidated substances ranges from 1.5 to 2.0 [174]; however, for most materials, the values of tortuosity are unknown. Therefore, the effective diffusion coefficients of porous media have to be measured by experiments. In some studies, the ratio of the effective diffusion coefficient to the bulk diffusion coefficient is referred to as diffusibility.

In practical conditions, the diffusion process in an operating fuel cell is difficult to be experimentally studied. Therefore, the modeling approach has been broadly employed to study the mass transport in fuel cell porous components, in which the transport coefficient based on the structure of the porous media is important for the modeling accuracy. Many empirical models of effective diffusion coefficients in porous media, such as GDLs, MPLs, and CLs, have been developed based on the CL structure (e.g., porosity and CL composition). The most commonly used models for fuel cells are summarized in Table 5, including Bruggeman model [176, 177], Neale and Nader model [178], Tomadakis and Sotirchos model [179], Mezedur model [139], Zamel model [177], and Das model [180].

**Table 5:** Models to predict the effective diffusion coefficient of porous materials

Model	Effective diffusion coefficient	Note	Eq.	Ref.
Bruggeman	${D}_{\text{eff}}={D}_{\text{b}}{\varepsilon }^{1.5}$	Spherical particles	(17)	[176, 181]
Neale and Nader	${D}_{\text{eff}}={D}_{\text{b}}2\varepsilon /(3-\varepsilon )$	Spherical particles	(18)	[178]
Tomadakis and Sotirchos	${D}_{\text{eff}}={D}_{\text{b}}\varepsilon {[(\varepsilon -0.037)/0.963]}^{0.661}$	Fibers	(19)	[179]
Mezedur et al.	${D}_{\text{eff}}={D}_{\text{b}}{[1-(1-\varepsilon )}^{0.46}]\,(0\leqslant \varepsilon \leqslant 0.65)$	Tetragonal network	(20)	[139]
Zamel et al.	$D_{{{\text{eff}}}} = D_{{\text{b}}} \left\{ {1 - 2.76\varepsilon \,{\text{cos}}h\left( {3\varepsilon - 1.92} \right)\left[ {\frac{{3\left( {1 - \varepsilon } \right)}}{3 - \varepsilon }} \right]} \right\}\quad {\text{for }}(0.33 \leqslant \varepsilon \leqslant 1)$	Fibers	(21)	[177]
Das et al.	${D}_{\text{eff}}={D}_{\text{b}}\left[1-\frac{3(1-\varepsilon )}{\frac{3{D}_{\text{b}}}{{D}_{\text{b}}-\frac{2{\omega D}_{\text{m}}}{3-\omega }}-\varepsilon }\right]$	Catalyst layers	(22)	[180]

*D*_m_ is the diffusivity in ionomer, and *ω* is the volume fraction of ionomer in catalyst layers

### Permeability

#### Darcy’s Law

The permeability of the porous media in PEM fuel cells represents the capability of mass transfer via convection driven by pressure gradients. The relation between the superficial velocity of the fluids penetrating the porous specimens and pressure gradient is governed by Darcy’s law as follows:

23 $$ - \frac{{{\text{d}}p}}{{{\text{d}}x}} = \frac{\mu u}{{K_{0} }} $$

where *u* is the superficial velocity in [m s^−1^], *μ* is the dynamic viscosity in [Pa s], and *K*_0_ is the permeability in [m^2^].

It should be noted that Darcy’s law with a linear relation between the superficial velocity and pressure gradient is valid only when the flow rate is small. However, for high flow velocity, the velocity–pressure–gradient relation is often nonlinear as the inertial effect cannot be neglected, where Darcy’s law has to be modified and Forchheimer equation has to be applied [115, 160, 169, 182]:

24 $$ - \frac{{{\text{d}}p}}{{{\text{d}}x}} = \frac{\mu u}{K} + \beta \rho u^{2} $$

where *β* is the non-Darcy coefficient in [m^−1^], and *ρ* is the density in [kg m^−3^]. In some studies, *K* is called viscous permeability in [m^2^], and 1/*β* is called inertial permeability in [m] [169].

Under certain circumstances, liquid water exists and floods in the CL pores, which inhibits the fuel cell performance by blocking the reactant transport pathways and the reactive surfaces. When liquid water exists, the convective gas and liquid flow in the pores will interact with each other, and the permeability of the CL for the liquid and gas phases will be altered due to the two-phase flow. The actual permeability of the CL for both gas and liquid phases is called relative permeability, which is usually smaller than the intrinsic permeability. For a two-phase flow system, the velocity of each phase, governed by Darcy’s law, can be given by the following equation:

25 $$ u_{i} = - \frac{{K_{0} K_{{{\text{r}},i}} }}{{\mu_{i} }}\frac{{{\text{d}}p_{i} }}{{{\text{d}}x}} $$

where *u*_*i*_ is the superficial velocity of phase *i* in [m s^−1^], *K*_0_ is intrinsic permeability measured by a single-phase flow in [m^2^], *K*_r,*i*_ is the dimensionless relative permeability for phase *i*, *μ*_*i*_ is the dynamic viscosity in [Pa s], and *p*_*i*_ is the partial pressure of phase *i* in [Pa].

For the air–water system in fuel cells, the air velocity can be calculated as follows:

26 $$ u_{{{\text{air}}}} = - \frac{{K_{0} K_{{{\text{r}},{\text{air}}}} }}{{\mu_{{{\text{air}}}} }}\frac{{{\text{d}}p_{{{\text{air}}}} }}{{{\text{d}}x}} $$

where the subscript “air” denotes the properties of air.

The velocity of liquid water can be calculated via the following equation:

27 $$ u_{{\text{w}}} = - \frac{{K_{0} K_{{{\text{r}},{\text{w}}}} }}{{\mu_{{\text{w}}} }}\frac{{{\text{d}}p_{{\text{w}}} }}{{{\text{d}}x}} $$

where the subscript “w” denotes the properties of liquid water.

The relation between the gas- and liquid-phase pressure can be calculated as follows in terms of capillary pressure.

28 $${p}_{\mathrm{c}}={p}_{\mathrm{air}}-{p}_{\mathrm{w}}$$

#### Experimental Methods for Intrinsic Permeability

**Fig. 17:**
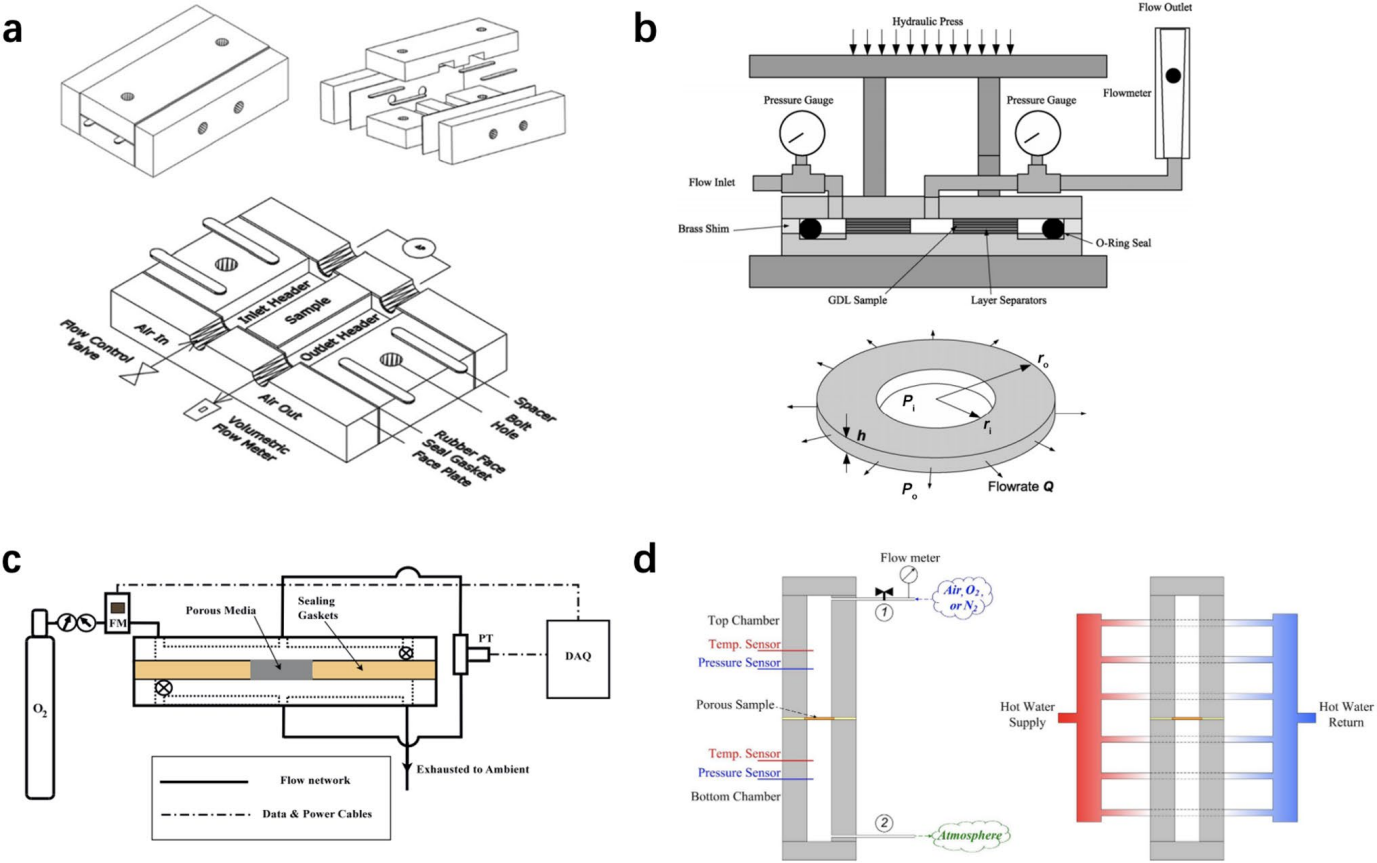
Different types of experimental setups for permeability measurement. **a** In-plane permeability by Gostick et al. [192]. Reprinted with permission from Ref. [192]. Copyright © 2006, Elsevier. **b** In-plane permeability by Feser et al. [193]. Adapted with permission from Ref. [193]. Copyright © 2006, Elsevier. **c** Through-plane permeability by Pant et al. [169]. Reprinted with permission from Ref. [169]. Copyright © 2012, Elsevier. **d** Through-plane permeability by Zhao et al. [161]. Reprinted with permission from Ref. [161]. Copyright © 2018, Elsevier

The permeability of the porous material is usually determined by measuring the pressure difference across the specimen with known thickness at given flow rates via Darcy’s law [182–191]. Many experimental apparatuses have been developed for measuring the intrinsic permeability of fuel cell electrodes in different directions. Gostick et al. [192] developed a test instrument to measure the in-plane permeability, as shown in Fig. 17a. During the experiment, the porous specimen is compressed by two plates with adjustable thickness via feeler gauges. The air flow rate is monitored by a flow meter at the outlet, and the inlet pressure is measured by a pressure transducer assuming atmospheric pressure at the outlet. For low-velocity flow, the permeability is calculated by solving Darcy’s law by the following equation:

29 $$\frac{\left({p}_{\mathrm{in}}^{2}-{p}_{\mathrm{out}}^{2}\right){M}_{\mathrm{air}}}{2l{R}_{\mathrm{u}}T}=\frac{\mu {J}_{\mathrm{m}}}{K}$$

where *l* is the length of the specimen in [m], and *J*_m_ is the mass flux in [kg m^−2^ s^−1^].

For high velocities, the inertial pressure loss is not negligible, and the permeability *K* and the inertial coefficient *β* are determined by fitting the experimental data by the following equation (the integral form of Forchheimer equation).

30 $$\frac{\left({p}_{\mathrm{in}}^{2}-{p}_{\mathrm{out}}^{2}\right){M}_{\mathrm{air}}}{2l{R}_{\mathrm{u}}T}=\frac{\mu {J}_{\mathrm{m}}}{K}+\beta {{J}_{\mathrm{m}}}^{2}$$

Feser et al. [193] designed a radial flow apparatus for the in-plane permeability measurement, as shown in Fig. 17b. The impregnating fluid can be either liquid or gas, and the porous sample can be compressed at various levels. For gas permeability, air pressure is measured at both inlet and outlet, while for liquid permeability, only inlet pressure is measured. By integrating Darcy’s law for a radial configuration, the permeability can be calculated by the following equation:

31 $${Q}_{\mathrm{out}}=\frac{\uppi K\delta }{\mu \mathrm{ln}\left({r}_{\mathrm{out}}/{r}_\text{in}\right)}\frac{\left({p}_{\mathrm{in}}^{2}-{p}_{\mathrm{out}}^{2}\right)}{{p}_{\mathrm{out}}}$$

where *Q* is the outlet flow rate in [m^3^ s^−1^], *δ* is the thickness of specimens, and *r* is the radius. By measuring the permeability of the same glass fabric sample using a single-phase liquid and gas, it is found that the difference in the permeability is very small, with the liquid permeability of 6.02 × 10^−13^ m^2^ and the gas permeability of 5.89 × 10^−13^ m^2^.

Pant et al. [169] modified a diffusion bridge setup to measure the pressure drop across the porous media under given mass flow rates, as shown in Fig. 17c. With this apparatus, the viscous and inertial through-plane permeability can be derived for GDLs and MPLs. Zhao et al. [161] modified a Loschmidt cell to measure the through-plane permeability, as shown in Fig. 17d. By measuring the inlet and outlet pressure under the controllable flow rate of different gases (e.g., N_2_, O_2_, and air) under different temperatures, the permeability coefficient can be determined. By analyzing the difference between uncatalyzed GDL and catalyzed GDLs using a resistance network theory based on the following equation, the permeability of CLs alone is indirectly measured in [161] because the CLs cannot stand alone without supports. The measurement uncertainties depend on the thickness of the CLs and the nature of the porous supports.

32 $${K}_{\mathrm{CL}}^{\text{eff}}=\left({\delta }_{\mathrm{sub}\_\mathrm{CL}}-{\delta }_{\mathrm{sub}}\right){\left(\frac{{\delta }_{\mathrm{sub}\_\mathrm{CL}}}{{K}_{\mathrm{sub}\_\mathrm{CL}}}-\frac{{\delta }_{\mathrm{sub}}}{{K}_{\mathrm{sub}}}\right)}^{-1}$$

where the subscripts “sub”, “CL”, and “sub_CL” denote the properties of the substrate, CL, and catalyzed substrate, respectively.

Table 6 summarizes the key data on the intrinsic permeability of the CLs from both experimental and modeling input parameters. It should be noted that the existing experimental studies are mainly focused on the GDLs, and the intrinsic permeability of the carbon paper is around 6×10^−12^–70×10^−12^  m^2^, and that of the GDLs (i.e., a carbon paper + a MPL made of carbon particles and hydrophobic agents) is about 0.3×10^−12^–1.1×10^−12^ m^2^ [161]. The experimental results in [161] suggest that the intrinsic permeability of the CLs is much smaller than that of GDLs. The intrinsic permeability of the CLs with the Pt loadings of 0.1–0.4 mg_Pt_ cm^−2^ prepared by mixing 25 wt% ionomer with different types of Pt/C catalysts (i.e., 30% and 60% Pt in Pt/C) is within 1.5×10^−15^–3.7×10^−15^ m^2^ (see Table 6 for more details). This minor discrepancy is due to the structural difference in the CLs using different types of catalyst particles.

**Table 6:** Through-plane intrinsic permeability of the catalyst layers from experimental results or modeling input parameters

Catalyst layer composition	Thickness/µm	Working fluid	Through-plane permeability/(10^−12^ m^2^)	Experimental or for modeling	Ref.
30% Pt/C, 25 wt% ionomer, 0.1 mg_Pt_ cm^−2^	4.4	Dry air	0.001 5	Experimental	[161]
30% Pt/C, 25 wt% ionomer, 0.2 mg_Pt_ cm^−2^	11.5	Dry air	0.002 6	Experimental	[161]
30% Pt/C, 25 wt% ionomer, 0.3 mg_Pt_ cm^−2^	17.4	Dry air	0.003 6	Experimental	[161]
30% Pt/C, 25 wt% ionomer, 0.4 mg_Pt_ cm^−2^	21.4	Dry air	0.003 7	Experimental	[161]
60% Pt/C, 25 wt% ionomer, 0.1 mg_Pt_ cm^−2^	3.0	Dry air	0.001 6	Experimental	[161]
60% Pt/C, 25 wt% ionomer, 0.2 mg_Pt_ cm^−2^	4.8	Dry air	0.001 5	Experimental	[161]
60% Pt/C, 25 wt% ionomer, 0.3 mg_Pt_ cm^−2^	7.6	Dry air	0.002 2	Experimental	[161]
60% Pt/C, 25 wt% ionomer, 0.4 mg_Pt_ cm^−2^	9.4	Dry air	0.002 2	Experimental	[161]
Composition not given; porosity: 0.6	35	H_2_ + H_2_O Air + H_2_O	0.1	For modeling	[194]
Composition not given; porosity: 0.15	15	H_2_ + H_2_O Dry O_2_	0.2	For modeling	[195]
Anode: Pt/Ru/C = 2:1:2, 15 wt% ionomer, 1.5 mg cm^−2^ Cathode: Pt/C = 1:4, 30 wt% ionomer, 1.0 mg cm^−2^	30 (anode) 20 (cathode)	Methanol/water Air	1.0 1.0	For modeling	[196]
Ionomer volume fraction: 0.23; porosity: 0.3	5 (anode) 10 (cathode)	H_2_ + H_2_O Air or O_2_ + H_2_O	0.1	For modeling	[197]
Composition not given; porosity: 0.4	10	H_2_ + H_2_O, O_2_ + H_2_O	0.000 1	For modeling	[198]

It should be mentioned that the intrinsic permeability of the CLs used for modeling significantly varies by several orders of magnitude from 10^−16^ to 10^−12^ m^2^ (data sources were not provided in these studies) [194–198]. This can lead to inaccurate simulation results, especially when the transport mechanisms of gas reactants through convection is considered. The discrepancy in the permeability values between experimental studies and modeling input parameters suggested that the accurate experimental data on the permeability of CLs are urgently needed to improve model development. These values should be carefully implemented when convection mass transfer in porous media is important.

#### Empirical Models for Intrinsic Permeability

The intrinsic permeability mainly depends on the porous structure under dehydrated conditions. To calculate the intrinsic permeability of different porous components in PEM fuel cells, many models have been established (see Table 7). Tomadakis et al. [179, 186, 199, 200] established several models based on porous media made of random overlapping or non-overlapping fibers in in-plane or through-plane directions, which are a strong function of fiber diameter, porosity, and fiber orientation. Models are also developed for porous materials made of spherical particles [115, 201, 202], which are dependent on particle size and porosity. For the CLs, these models may not be suitable as the CL structure usually consists of Pt nanoparticles, carbon support, and ionomer with different ratios, as well as various agglomerates of the Pt/C particles. The complicated CL structure makes it challenging to build a universal model for CLs unless sufficient experimental data are available.

**Table 7:** Models to predict the intrinsic permeability of the porous media in PEM fuel cells

Model	Material	Note	Eq.	Ref.
$K=\frac{\varepsilon }{{K}_{\mathrm{c}}}{\left(\frac{{V}_{\text{p}}}{{S}_{\text{p}}}\right)}^{2}$	General porous media	*ε*—porosity *K*_c_—Kozeny constant *V*_pore_—pore volume *S*_pore_—pore surface	(33)	[186]
$K=\frac{{r}^{2}\varepsilon }{4{K}_{\mathrm{c}}(\text{ln }\varepsilon {)}^{2}}$	Random overlapping fibers	*r*—fiber radius *ε*—porosity *K*_c_—Kozeny constant	(34)	[186, 199]
$K=\frac{{r}^{2}{\varepsilon }^{3}}{4{K}_{\mathrm{c}}(1-\varepsilon {)}^{2}}$	Random non-overlapping fibers	*r*—fiber radius *ε*—porosity *K*_c_—Kozeny constant	(35)	[186, 200]
$K={r}^{2}\frac{\varepsilon {(\varepsilon -0.11)}^{\alpha +2}}{8(ln \,\varepsilon {)}^{2} (1-\varepsilon {)}^{\alpha }[(\alpha +1)\varepsilon -0.11{]}^{2}}$	Fibers	*r*—fiber radius *ε*—porosity *α*—0.785 through-plane 0.521 in-plane	(36)	[179, 186]
$K=\frac{{d}^{2}{\varepsilon }^{3}}{150(1-\varepsilon {)}^{2}}$	Spherical particles	*d*—particle diameter *ε*—porosity	(37)	[115, 201]
$K=\frac{{d}^{2}{\varepsilon }^{5.5}}{5.88} (0.35<\varepsilon <0.7)$	Spherical particles	*d*—particle diameter *ε*—porosity	(38)	[115, 202]

Kozeny constant is an unknown parameter for most porous materials

Klinkenberg [189] indicated that the intrinsic permeability may also be affected by the types of fluids, and the experimental data suggested that the intrinsic permeability of a glass filter for air is 28% lower than that for hydrogen under given conditions. Zamora et al. [203] demonstrated that the hydrogen permeability of the MPLs is around 20% higher than that of air and oxygen, suggesting that the gas with a smaller molecular size can penetrate the same porous media with less resistance.

#### Experimental Methods for Relative Permeability

In the practical operation of PEM fuel cells, water often exists in the form of both liquid and vapor in the CLs. Many numerical models apply Darcy’s law to study the convection of both the liquid and gas phases in the porous fuel cell components. For the gas phase flow, the permeability value is affected by the presence of liquid due to the gas–liquid interaction. The practical permeability of gas and liquid flows in the two-phase flow system is lower than the intrinsic permeability, and the ratio of the actual permeability to the intrinsic permeability is called the relative permeability.

**Fig. 18:**
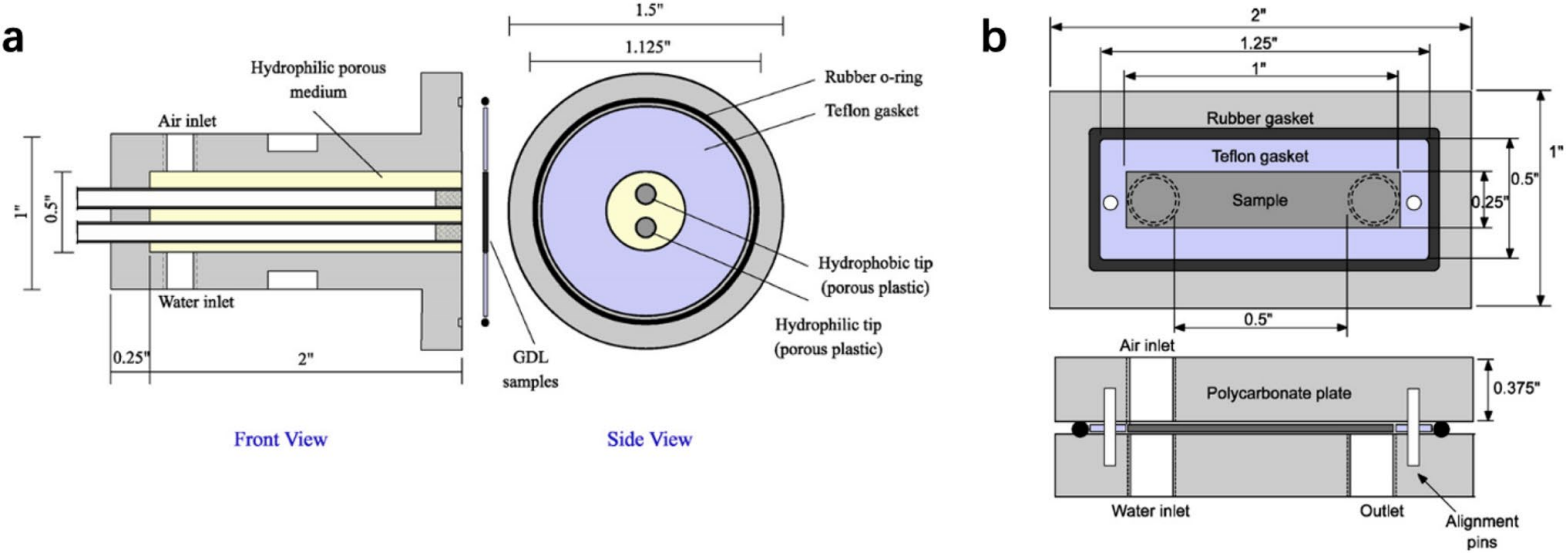
Schematic of the apparatus for measuring the relative permeability: **a** through-plane and **b** in-plane. Reprinted with permission from Ref. [183]. Copyright © 2010, Elsevier

The measurements of the relative permeability of a porous material for a multi-phase system can be accomplished by steady-state, unsteady-state, capillary pressure, centrifuge, and other methods [183, 204]. These methods are widely used for rock materials; however, experimental studies on the relative permeability for fuel cell components are very rare. Hussaini and Wang [183] developed a through-plane and an in-plane permeability apparatus to measure the relative permeability for a liquid–water–air system, as shown in Fig. 18. The relative permeability is measured based on steady-state methods, in which the fluids are forced to pass through the porous material at a given ratio until the saturation and pressure become stable. By changing the liquid–gas ratio, the flow rates of each phase at various saturation levels can be obtained. Specifically for the through-plane permeability measurement (see Fig. 18a), liquid water and air are premixed through a hydrophilic porous plastic material in upstream. The homogeneous mixture then flows through the test specimens, across which the pressure drop is estimated by the difference of system pressure drop with and without test specimens. The pressure drop of the liquid and gas phases across the specimen is found to be identical. To ensure the measurement accuracy, a few layers of samples are often stacked in the test to generate a sufficiently large pressure drop. For the in-plane permeability measurement (see Fig. 18b), the test specimens are slightly compressed to ensure no leakage during the test. Water and air enter the specimens from one side, pass through the specimens in the in-plane direction, and flow out from the other side with mixed water and air. In both test rigs, the test specimens can be quickly removed from the testing apparatus for measuring the saturation by an ex situ gravimetric method. The saturation is measured by the weight change of a wet specimen in comparison with its dry state. The average saturation ${\varPhi}_{\text{l}}$ can be determined by the following equation.

39 $${\varPhi}_{\text{l}}=\frac{\Delta m}{{\rho}_{\text{w}}\varepsilon{V}_{\text{b}}}$$

where ∆*m* is the mass change of the wet specimens in comparison with its dry state in [kg] and *ρ*_w_ is the density of liquid water in [kg m^−3^].

The relation between the relative permeability and saturation can be thus determined, and empirical correlations are given for various carbon paper or cloth in the in-plane direction (see Table 8). Correlation models for the in-plane relative permeability of the porous carbon paper or cloth are developed based on experimental data, as shown in Table 8. Unfortunately, the accuracy of through-plane relative permeability measurement is not sound, and it is suggested to further improve the measurement accuracy [183].

**Table 8:** Relative permeability of porous media in PEM fuel cells

Model	Fuel cell component	Source	Direction	Eq.	Ref.
$$\left\{ {\begin{array}{*{20}l} {K_{{\text{r,g}}} = \left( {1 - {\varPhi}_{{\text{l}}} } \right)^{3.0} } \hfill \\ {K_{{\text{r,l}}} = {\varPhi}_{{\text{l}}}^{3.0} } \hfill \\ \end{array} } \right.$$	CL	Not specified	Not specified	(40)	[205]
$$\left\{ {\begin{array}{*{20}l} {K_{{\text{r,g}}} = \left( {1 - {\varPhi}_{{\text{l}}} } \right)^{4.5} } \hfill \\ {K_{{\text{r,l}}} = {\varPhi}_{{\text{l}}}^{4.5} } \hfill \\ \end{array} } \right.$$	Toray 060 GDL with 10% PTFE	Not specified	Not specified	(41)	[205]
$$\left\{ {\begin{array}{*{20}l} {K_{{\text{r,g}}} = \left( {1 - {\varPhi}_{{\text{l}}}^{2} } \right)^{4} } \hfill \\ {K_{{\text{r,l}}} = {\varPhi}_{{\text{l}}}^{4} } \hfill \\ \end{array} } \right.$$	Carbon paper	Experimental	In-plane	(42)	[183]
$$\left\{ {\begin{array}{*{20}l} {K_{{\text{r,g}}} = \left( {1 - {\varPhi}_{{\text{l}}} } \right)^{3} } \hfill \\ {K_{{\text{r,l}}} = {\varPhi}_{{\text{l}}}^{4} } \hfill \\ \end{array} } \right.$$	Carbon cloth	Experimental	In-plane	(43)	[183]

#### Empirical Models for Relative Permeability

Due to the lack of experimental techniques, the relative permeability of the gas and liquid phases is often calculated by using empirical models. The relative permeability is a function of liquid volume fraction in the porous media, and a summary of the commonly employed correlation in fuel cell modeling is presented in Table 8. It should be noted that the accuracy of these models should be carefully justified as the experimental data are very limited in literature, especially for the thin CLs.

### Capillary Pressure

#### Young–Laplace Equation

**Fig. 19:**
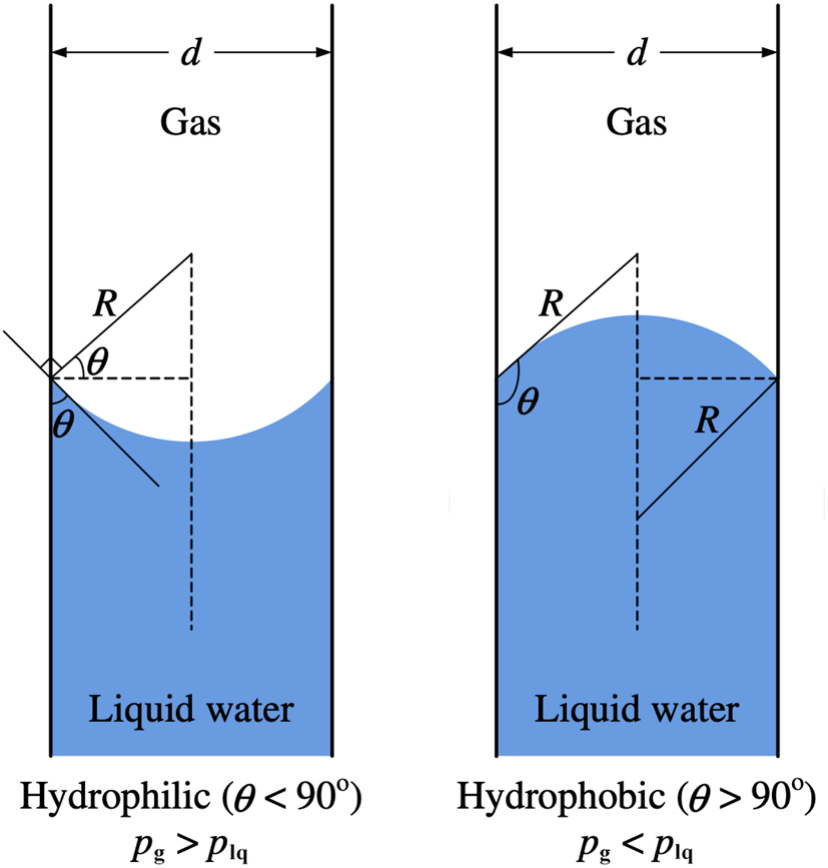
Schematic of liquid in a cylindrical pore. Reprinted with permission from Ref. [81]. Copyright © 2011, Elsevier

The capillary pressure refers to the pressure drop across a static interface between two immiscible fluids [160, 182], as shown in Fig. 19. A modified Young–Laplace equation (or Washburn equation) can be used to describe the relation among the capillary pressure, surface tension, pore radius, and contact angle.

44 $$\Delta p=\sigma \left(\frac{1}{{r}_{1}}+\frac{1}{{r}_{2}}\right)=\frac{2\sigma \mathrm{cos }\,\theta }{{r}_{\text{p}}}$$

where ∆*p* is the pressure difference in [Pa] across the liquid–gas interface, *r*_1_ and *r*_2_ are the interfacial curvatures in [m], and *r*_p_ is the radius (or half pore size) of the associated pores in [m].

#### Experimental Methods for Capillary Pressure

The capillary pressure of the liquid water in the porous CL can be affected by various factors, including materials, composition, pore structure, surface tension, and most importantly liquid saturation. For a given porous GDL or CL, the capillary pressure is mainly affected by the saturation levels, which determines the mass transport resistance and overall performance of PEM fuel cells when operated at high current density regions. Therefore, many experimental methods have been applied for the porous media in PEM fuel cells to determine the capillary pressure–saturation relation.

Gostick et al. [206] experimentally studied the capillary pressure against saturation using the method of mercury intrusion and MSP. In the mercury intrusion method, the mercury’s saturation (nonwetting) in an initially dry specimen is measured in terms of capillary pressure [127], and the mercury’s capillary pressure is corrected by that measured by water via the following equation at different given saturation levels.

45 $$ r_{{{\text{eq}}}} = \frac{{2\sigma_{{{\text{Hg}}{ - }{\text{air}}}} {\text{cos}} \,\theta_{{{\text{Hg}}{ - }{\text{air}}}} }}{{p_{{{\text{c,Hg}}{ - }{\text{air}}}} }} = \frac{{2\sigma_{{{\text{water}}{ - }{\text{air}}}} {\text{cos}} \,\theta_{{{\text{water}}{ - }{\text{air}}}} }}{{p_{{{\text{c,water}}{ - }{\text{air}}}} }} $$

where *r*_eq_ is the maximum radius of pores occupied by the liquid when mercury and water are under equivalent saturation levels in [m]. It should be noted that the effect of contact angle in the individual pore with a particular size or a certain PTFE content is not taken into account, and constant contact angles are assumed for all surfaces [206].

The MSP can directly measure the relation between the capillary pressure and saturation without considering the contact angles in individual pores. Similar to the PSD measurement by MSP, the capillary pressure can be measured based on the phenomena of capillary equilibrium by analyzing the mass change of the liquid in the test specimen and comparing it to the standard specimens having a known capillary–pressure–saturation relation. During the tests, the test and standard porous specimens are fully saturated in liquid, closely contacted, and slowly dehydrated at different saturation levels. The mass change is measured periodically, transferred to volume change, and related to saturation levels. The capillary pressure at different saturation levels is determined from the standard capillary–pressure–saturation curve.

#### Empirical Models for Capillary Pressure

The capillary pressure of the liquid water in the porous CLs is dependent on various parameters, including composition, pore structure, surface tension, liquid saturation, and even compression conditions. Many empirical models have been developed for the porous media to correlate the capillary pressure with the saturation in PEM fuel cells, as summarized in Table 9. The Leverett J-function [see Eq. (46) in Table 9] [81, 207, 208] is one of the most commonly used capillary pressure–saturation correlation models in porous media of PEM fuel cells, in which the capillary pressure is a function of intrinsic permeability, porosity, surface tension, water saturation, and contact angle. Kumbur et al. [209] further introduced the compression and temperature effects into the calculation of capillary pressure in GDLs with varied PTFE contents [see Eq. (47) for example]. Ye and Nguyen [205] used two correlation models as a function of saturation levels by curve fitting with experimental data for GDLs [Eq. (48)] and CLs [Eq. (49)], respectively. As can be seen, the correlation models of the capillary pressure–saturation relation for CLs are very rare in literature, and these models are not suitable for all CLs as the composition and materials applied are varied from case to case.

**Table 9:** Correlations between capillary pressure and saturation of the porous media in PEM fuel cells

Model	Porous media	Eq.	Ref.
$$p_{{{\text{ca}}}} = \left\{ {\begin{array}{*{20}l} {\sigma \cos \theta \sqrt {\frac{\varepsilon }{{K_{0} }}} \left[ {1.42\left( {1 - {\varPhi}_\text{l} } \right) - 2.12\left( {1 - {\varPhi}_\text{l} } \right)^{2} + 1.26\left( {1 - {\varPhi}_\text{l} } \right)^{3} } \right],} \hfill & {\theta < 90^\circ } \hfill \\ {\sigma \cos \theta \sqrt {\frac{\varepsilon }{{K_{0} }}} \left( {1.42{\varPhi}_\text{l} - 2.12{\varPhi}_{\rm l}^{2} + 1.26{\varPhi}_\text{l}^{3} } \right),} \hfill & {\theta > 90^\circ } \hfill \\ \end{array} } \right.$$	GDL	(46)	[81, 207, 208]
$$p_{{{\text{ca}}}} = \left\{ {\begin{array}{*{20}l} {\sigma \left( \frac{293}{T} \right)^{6} 2^{0.4C} \sqrt {\frac{\varepsilon }{{K_{{0}} }}} x\left( {0.046\,9 - 0.001\,52x - 0.040\,6{\varPhi}_{{\text{l}}}^{2} + 0.143{\varPhi}_{{\text{l}}}^{3} } \right) + 0.056\,1\,{\text{ln }}{\varPhi}_{{\text{l}}} }, \hfill & {0 < {\varPhi}_{{\text{l}}} < 0.5} \hfill \\ {\sigma \left( \frac{293}{T} \right)^{6} 2^{0.4C} \sqrt {\frac{\varepsilon }{{K_{{0}} }}} x\left( {1.534 - 0.029\,3x - 12.68{\varPhi}_{{\text{l}}}^{2} + 18.824{\varPhi}_{{\text{l}}}^{3} } \right) + 3.416\,{\text{ln }}{\varPhi}_{{\text{l}}} }, \hfill & {0.5 \leqslant {\varPhi}_{{\text{l}}} \leqslant 0.65} \hfill \\ {\sigma \left( \frac{293}{T} \right)^{6} 2^{0.4C} \sqrt {\frac{\varepsilon }{{K_{{0}} }}} x\left( {1.7 - 0.032\,4x - 14.1{\varPhi}_{{\text{l}}}^{2} + 20.9{\varPhi}_{{\text{l}}}^{3} } \right) + 3.79\,{\text{ln }}{\varPhi}_{{\text{l}}} }, \hfill & {0.65 < {\varPhi}_{{\text{l}}} < 1 } \hfill \\ \end{array} } \right.$$ Valid 20 °C < *T* < 80 °C and 0 < *C* < 1.4 MPa	SGL 24 series GDL coated with MPL	(47)	[209]
${p}_{\text{ca}}=-2.09[{{\text{e}}^{44.9\left({\varPhi }_{\text{l}}-0.321\right)}-\text{e}^{-22.2\left({\varPhi }_{\text{l}}-0.321\right)}}]+35.6$	Toray-060 GDL with 10% PTFE	(48)	[205]
${p}_{\text{ca}}=-2\,431\left[{\text{e}}^{92.36\left({\varPhi }_{\text{l}}-0.567\right)}{-\text{e}}^{-0.008\,8\left({\varPhi }_{\text{l}}-0.567\right)}\right]-2\,395$	CL	(49)	[205]

*C* is compression pressure in MPa; *x* is the weight ratio of PTFE in GDL

### Contact Angle

#### Young’s Equation

**Fig. 20:**
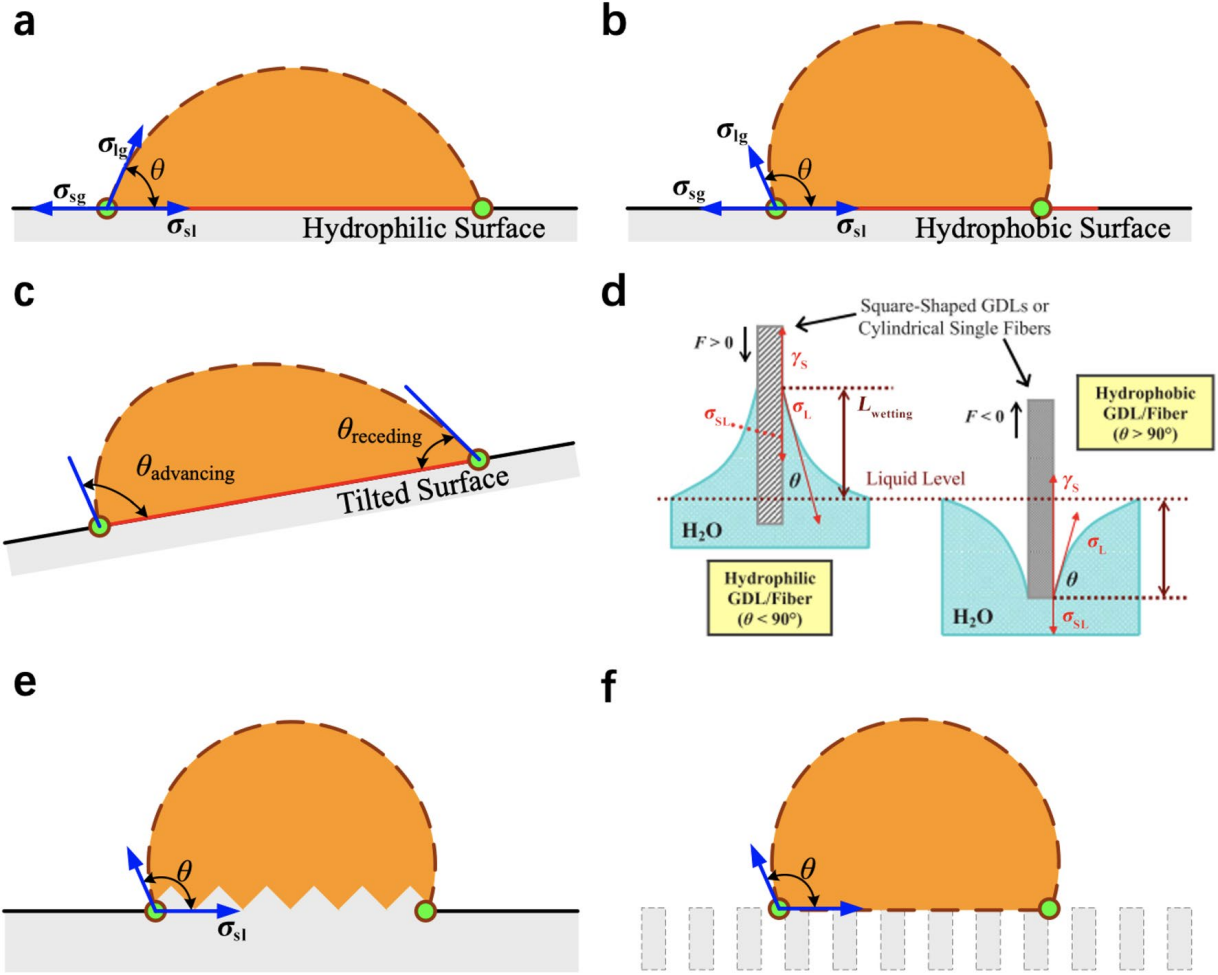
Schematic of contact angle on different types of surfaces. **a** Hydrophilic surface. **b** Hydrophobic surface. **c** Tilted surface. **d** Wihelmy method. Adapted with permission from Ref. [212]. Copyright © 2010, the Electrochemical Society. **e** Rough surface. **f** Porous surface

The contact angle of liquid water on the surface of CLs is governed by Young’s equation [210].

50 $$\text{cos}\,{\theta }_{\text{app}}=\frac{{\sigma }_{\text{sg}}-{\sigma }_{\text{sl}}}{{\sigma }_{\text{lg}}}$$

where *θ*_app_ is the apparent contact angle in [rad], *σ* denotes the surface tension between solid–gas (sg), solid–liquid (sl), and liquid–gas (lg) interfaces. The value of contact angle depends on the nature of materials, composition, surface roughness factor, and pore structure, and the contact angle is important for the water management and structure change in CLs [211]. Based on the contact angle, the surfaces are often categorized into hydrophilic (< 90°) or hydrophobic (> 90°), as shown in Fig. 20a, b.

#### Experimental Methods for Contact Angle

Various experimental methods have been established to study the static contact angle of water on the surface of porous media in fuel cells. The static contact angle is often measured by placing a sessile droplet on a flat surface and analyzing the geometry of the drop shape (see Fig. 20a, b for example). The size of the drop should be properly selected to minimize the gravity effect on the shape of drops and the value should be recorded prior to substantial evaporation [213]. Generally, the contact angle can be directly read from the side view images, and some studies obtained the contact angle by measuring the maximum height of the drop. Giesche [107] indicated that the contact angle can be calculated based on a sessile mercury drop on a flat surface in air as follows:

51 $$ {\text{cos }}\theta = 1 - \frac{{\rho_{{{\text{Hg}}}} gh_{{{\text{max}}}}^{2} }}{{2\sigma_{{{\text{Hg}}{ - }{\text{air}}}} }} $$

where *h*_max_ is the maximum height of the droplet in [m], and *g* is gravity acceleration in [m s^−2^].

The static contact angle is an important parameter to describe the wettability of liquid water on CLs; however, the contact angle should be carefully used for the calculation in an operating PEM fuel cell where liquid water is dynamic. The liquid water will be driven by a capillary pressure gradient or the interaction with gas flow [4]. During the transport of liquid water, the dynamic contact angle in different scenarios can be measured by various methods, including the sliding angle method and the Wihelmy method [212, 213]. In the sliding angle method, by measuring the contact angle before the drop slides on a gradually tilted surface or a flat surface with blowing gas, the advancing and receding angles can be determined (see Fig. 20c) [213]. In the Wihelmy method, Wood et al. [212] measured the contact angle of water on the singe fiber surface, using an apparatus as shown in Fig. 20d. Square-shaped samples or a single fiber extracted from GDL can be first submerged in liquid and then removed slowly. By analyzing the images from the side view, the dynamic contact angle can be derived. Table 10 summarizes the measured contact angle of water on CLs with different materials and compositions. The contact angle of liquid water on CLs ranges from 110° to 149°, suggesting that the CLs are mostly hydrophobic.

Recently, techniques have been developed to visualize the transport of liquid water in porous media of PEM fuel cells in operating modes. These methods include nuclear magnetic resonance, neutron imaging, synchrotron X-ray, and micro-tomography, which can visualize the liquid water under the lands of the bipolar plate or in the porous components that are opaque to optical access [214]. These techniques can be potentially employed to investigate the dynamic contact angle of water transport in porous CLs.

**Table 10:** Contact angle of water on CL surfaces from experimental results or modeling input parameters

CL composition	Thickness/μm	Substrate	Measurement method	Working fluid	Contact angle/(°)	Ref.
Ionomer-to-carbon ratio of 0.8, no Pt, hot pressed on ETFE	~8	Removable liner	Capillary penetration	Water	143	[215]
Ionomer-to-carbon ratio of 0.8, no Pt, hot pressed on ETFE, dried in a vacuum	~8	Removable liner	Capillary penetration	Water	149	[215]
Ionomer-to-carbon ratio of 0.7, Pt/C, conventional CL	1.25	GDL	Sessile drop	Water	111.2	[216]
Ionomer-to-carbon ratio of 0.7, Pt/C, C-doped CL	2.25–8.51	GDL	Sessile drop	Water	110.9	[216]
Ionomer-to-carbon ratio of 0.7, Pt/C, C@PTFE-doped CL	3.92	GDL	Sessile drop	Water	118.4	[216]
Ionomer-to-Pt black ratio of 0.11, no water additive in catalyst ink	–	Membrane	Sessile drop	Water	110.7	[217]
Ionomer-to-Pt black ratio of 0.11, water additive in catalyst ink	–	Membrane	Sessile drop	Water	128.8	[217]
Pt/C CL	–	–	Sessile drop	Water	134	[218]
Pt/C CL with commercial hydrophilic ZnO particle	–	–	Sessile drop	Water	122	[218]
Pt/C CL with homemade hydrophilic ZnO calcined @ 300 °C	–	–	Sessile drop	Water	116	[218]
Pt/C CL with homemade hydrophilic ZnO calcined @ 700 °C	–	–	Sessile drop	Water	124	[218]

ETFE denotes ethylene tetrafluoroethylene

#### Empirical Models for Contact Angle

When a liquid drop is placed on the surface of porous media, the value of the apparent contact angle can be affected by many factors, including the nature and composition of solid materials, roughness, and pore structure of the surface. Many theories have been developed to investigate the effects of these factors on the contact angle.

The relation between the roughness and contact angle is first given by Wenzel [219] as follows:

52 $$\mathrm{cos}\,{\theta }_{\mathrm{app}}={r}_{\mathrm{f}}\mathrm{cos}\,{\theta }_{\mathrm{s}}$$

where *θ*_app_ is the apparent contact angle on a rough surface (see Fig. 20e), *θ*_s_ is the contact angle on a smooth surface, and *r*_f_ is the roughness factor, which is the ratio of the actual surface area to the geometric area.

Cassie and Baxter [220] further explored the contact angle on a porous surface (see Fig. 20f):

53 $$\mathrm{cos}\,{\theta }_{\mathrm{app}}=\sum {f}_{i}\mathrm{cos}\,{\theta }_{i}$$

where *f*_*i*_ is the area fraction of water drop interacting with phase *i*, and *θ*_*i*_ is the reference contact angle on the smooth surface of each phase. For the liquid in the air, the contact angle is often assumed to be 180°.

### Effective Thermal Conductivity

#### Fourier’s Law

The heat transfer in solid fuel cell components is governed by Fourier’s law of conduction [160, 182]:

54 $$q=-{k}_{\mathrm{th}}^{\mathrm{eff}}\nabla T$$

where *q* is the heat flux in [W m^−2^], *k*_th_^eff^ is the effective thermal conductivity in [W m^−1^ K^−1^], and ∇*T* is the temperature gradient in [K m^−1^]. The effective thermal conductivity is determined by the materials, composition, and structure of the CLs and is often experimentally measured.

#### Experimental Methods for Effective Thermal Conductivity

Many experimental methods have been established to measure the effective thermal conductivity of the porous media in PEM fuel cells. Most of them are designed for the GDLs, and only the methods applied for CLs are reviewed in this section.

**Fig. 21:**
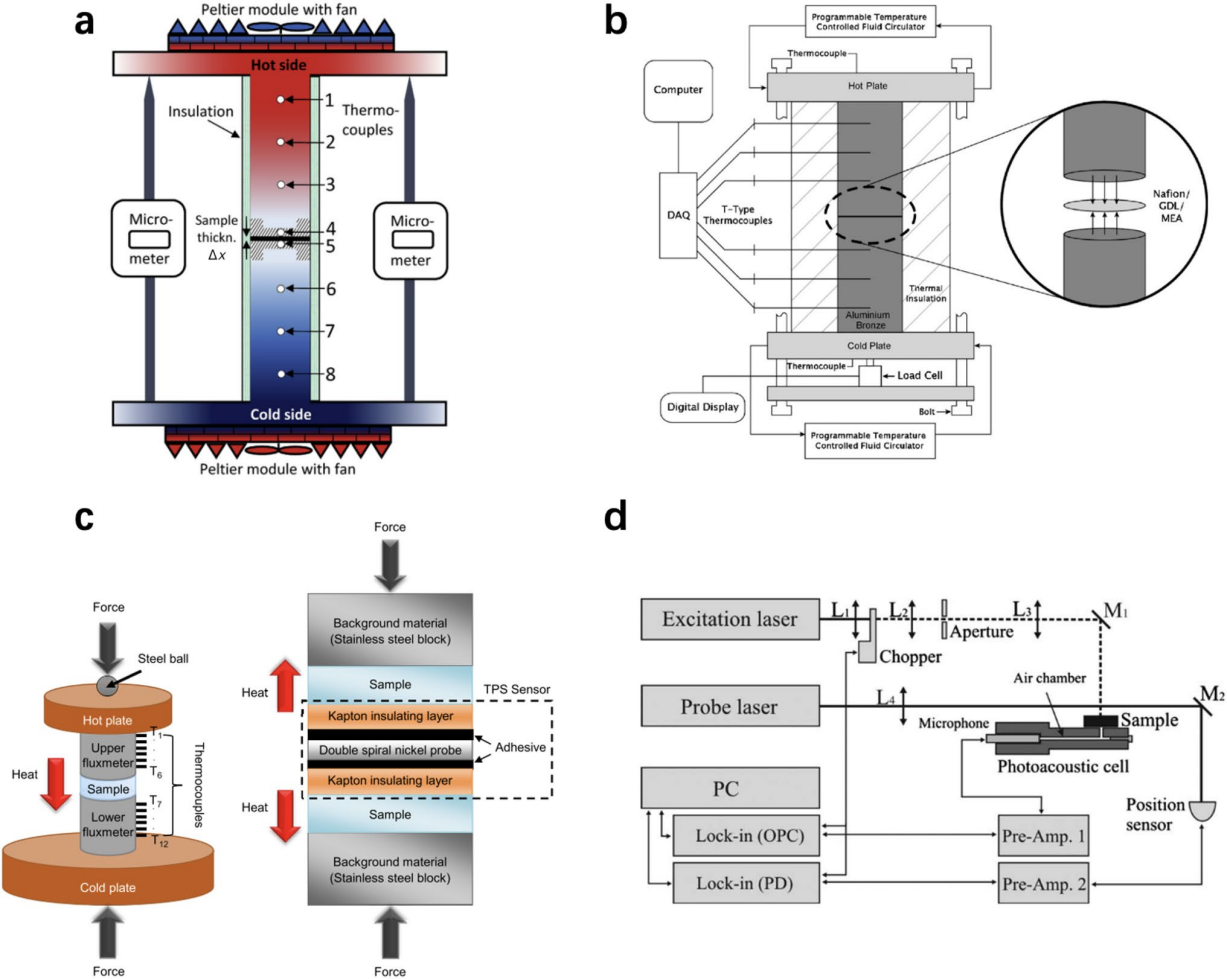
Schematic of various experimental setups for effective thermal conductivity of catalyst layers in different studies. **a** Bock et al. [221]. Reprinted with permission from Ref. [221]. Copyright © 2020, Elsevier. **b** Khandelwal and Mench [222]. Reprinted with permission from Ref. [222]. Copyright © 2006, Elsevier. **c** Ahadi et al. [223]. Reprinted with permission from Ref. [223]. Copyright © 2017, Elsevier. **d** Astrath et al. [225]. Reprinted with permission from Ref. [225]. Copyright © 2010, AIP Publishing

Bock et al. [221] measured the effective thermal conductivity of CLs using a custom-built apparatus as shown in Fig. 21a. Two CLs with the thickness of 10 μm are prepared by coating catalyst ink onto a copper foil via inkjet painting. The weight ratio of Nafion ionomer is 30% for both CLs, while the catalyst employed is different: one is graphitized-carbon-based Pt (46 wt% Pt/C Ketjen black) and the other one is non-graphitized-carbon-based Pt (40 wt% Pt/C Vulcan XC). Constant heat flux is imposed on the cylindrical steel by using the Peltier module based on thermoelectric theory. Six thermocouples are installed in the steel cylinders (1–3, 6–8) to monitor the heat flux passing through the cylinder and the sample, and two thermocouples (4 and 5) are installed inside an aluminum cap on each side of the test specimen, which helps determine the temperature gradient across the sample. The heat conductivity is thus calculated via Eq. (54). The experimental results suggest that the thermal conductivity of graphitized CL is (0.12 ± 0.05) W m^−1^ K^−1^, which is twice higher than that of non-graphitized CL [(0.061 ± 0.006) W m^−1^ K^−1^] at the compaction pressure of 10 bar (1 bar = 100 kPa). More details can be found in Table 11.

**Table 11:** Experimental data on through-plane effective thermal conductivity of catalyst layers

Catalyst layer composition	Thickness/µm	Compaction pressure/bar	Effective thermal conductivity/(W m^−1^ K^−1^)	Ref.
Graphitized CL (30 wt% Nafion, 46 wt% Pt/C Ketjen Black from TKK)	10	3	0.10 ± 0.03	[221]
Graphitized CL (30 wt% Nafion, 46 wt% Pt/C Ketjen Black from TKK)	10	5	0.11 ± 0.04	[221]
Graphitized CL (30 wt% Nafion, 46 wt% Pt/C Ketjen Black from TKK)	10	10	0.12 ± 0.05	[221]
Graphitized CL (30 wt% Nafion, 46 wt% Pt/C Ketjen Black from TKK)	10	15	0.14 ± 0.03	[221]
Graphitized CL (30 wt% Nafion, 46 wt% Pt/C Ketjen Black from TKK)	10	20	0.15 ± 0.05	[221]
Graphitized CL (30 wt% Nafion, 46 wt% Pt/C Ketjen Black from TKK)	10	23	0.19 ± 0.11	[221]
Non-graphitized CL (30 wt% Nafion, 40 wt% Pt/C Vulcan XC from Alfa Aesar)	10	3	0.038 ± 0.008	[221]
Non-graphitized CL (30 wt% Nafion, 40 wt% Pt/C Vulcan XC from Alfa Aesar)	10	5	0.048 ± 0.005	[221]
Non-graphitized CL (30 wt% Nafion, 40 wt% Pt/C Vulcan XC from Alfa Aesar)	10	10	0.061 ± 0.006	[221]
Non-graphitized CL (30 wt% Nafion, 40 wt% Pt/C Vulcan XC from Alfa Aesar)	10	15	0.070 ± 0.018	[221]
Non-graphitized CL (30 wt% Nafion, 40 wt% Pt/C Vulcan XC from Alfa Aesar)	10	20	0.10 ± 0.04	[221]
Non-graphitized CL (30 wt% Nafion, 40 wt% Pt/C Vulcan XC from Alfa Aesar)	10	23	0.114 ± 0.014	[221]
CL (0.5 mg cm^−2^ Pt/C)	25	~20	0.27 ± 0.05^a^	[222]
Non-hot-pressed CL by GHF method	8.74/23.86	4–14	0.214 ± 0.005	[223]
Hot-pressed CL by modified TPS method	14.98/28.72	2–14.4	0.218 ± 0.005	[223]
Dry CL (0 wt% Pt/C, carbon/Nafion = 1:1)	30/60	4.6–16.1	0.074–0.098	[224]
Dry CL (20 wt% Pt/C, carbon/Nafion = 1:1)	30/60	4.6–16.1	0.063–0.078	[224]
Dry CL (20 wt% Pt/C, carbon/Nafion = 1:2)	30/60	4.6–16.1	0.064–0.083	[224]
Wet CL (0 wt% Pt/C, carbon/Nafion = 1:1, water content = 70 ± 30)	30/60	4.6–16.1	0.10–0.15^b^	[224]
Wet CL (20 wt% Pt/C, carbon/Nafion = 1:1, water content = 40 ± 40)	30/60	4.6–16.1	0.11–0.13	[224]
Wet CL (20 wt% Pt/C, carbon/Nafion = 1:2, water content = 70 ± 30)	30/60	4.6–16.1	0.2–0.5^b^	[224]
Graphitized CL (30 wt% Nafion, 46 wt% Pt/C from TKK)	13–53	N/A	0.75 ± 0.07	[225]

^a^Effective thermal conductivity includes thermal contact resistance between CLs and diffusion media;

^b^Measurement error can be larger than 100%

Khandelwal and Mench [222] experimentally investigated the through-plane thermal conductivity of membrane, GDLs, and CLs under various temperature and pressure using an apparatus as shown in Fig. 21b. The tested samples are sandwiched by two aluminum bronze cylinders with known conductivity. Two backing plates are placed outside of cylinders, acting as the heat source and sinks, respectively. Heat flux is thus generated during the measurements, which can be estimated by the temperature gradient measured from six thermocouples installed in the standard cylinders. It should be noticed that the temperature drop across the test samples is not directly measured but estimated by linearly extrapolating the temperature in the standard materials to the edge of the test specimen. A commercial MEA (consisting of a membrane and two CLs) is tested, and the effective thermal conductivity taken contact resistance with GDL is measured to be 0.27 W m^−1^ K^−1^.

Ahadi et al. [223] measured the effective thermal conductivity of CLs by two approaches: guarded heat flow (GHF) and modified transient plane source (TPS) methods, as shown in Fig. 21c. In the GHF method, the test specimen is sandwiched between two flux meters and compressed by two plates with different temperatures, while in the TPS method, a circular double nickel spiral, serving as a heating device and a thermometer, is placed between two Kaption layers bounded by two test specimens. The effective thermal conductivity of the test specimens can be calculated by the increasing rate of temperature. The experimental results suggested that the CL deposited on an aluminum plate without hot pressing has the effective thermal conductivity of 0.214 W m^−1^ K^−1^ measured by the GHF method, while the conductivity of the CL coated on the ethylene tetrafluoroethylene (ETFE) substrate with hot pressing is found be 0.218 W m^−1^ K^−1^ measured by the TPS method.

Burheim et al. [224] tested the effective thermal conductivity of the dry and wet CLs using an apparatus similar to that in Fig. 21a. Three types of CLs composed of Pt/C particles with varied carbon-ionomer ratios are investigated at different water contents and pressure. The experimental results suggest that within the compaction pressure of 4.6–16.1 bar, the effective thermal conductivities of the CLs under investigation are in the range of 0.063–0.009 8 W m^−1^ K^−1^. For the wet CLs, the thermal conductivity is about twice higher than that of dry CLs at the given compaction pressure. It should be pointed out that the wet CLs are subject to high measurement errors, which are higher than 100% in some cases.

Astrath et al. [225] measured the effective thermal conductivity of CLs using a test rig combining an open photoacoustic cell (OPC) with photothermal detection (PD), as shown in Fig. 21d. A laser is used to generate an excitation beam to produce a top-hat intensity profile on the surfaces of uncoated specimen for OPC measurement and coated CLs for PD measurement. For the OPC measurement, the signals are detected by a microphone and a lock-in amplifier. For the PD measurement, a probe laser intercepts the mirage region, and the PD signals are detected by a position sensor connected to a lock-in PD amplifier. The CLs are deposited with various thicknesses (13–53 μm) on an aluminum foil, and the measured effective diffusivity is found to be around 0.75 W m^−1^ K^−1^ by analyzing the signal intensities with different modulation frequencies.

#### Empirical Models for Effective Thermal Conductivity

The temperature distribution and heat transfer in PEM fuel cell components are determined by the thermal conductivity of materials, and in the CLs, the effective thermal conductivity is often used to solve the energy balance equation assuming the computational domain is a mixture of catalyst, carbon, membrane, liquid, and gas [182]. The effective thermal conductivity is often calculated as an average property of different phases as follows:

55 $${k}_{\text{th}}^{\text{eff}}= f({k}_{\text{s}},{k}_{\text{m}},{k}_{\text{w}},{k}_{\text{g}}{{,\varPhi }_{\text{s}},\varPhi}_{\text{m}},{\varPhi}_{\text{w}},{\varPhi}_{\text{g}})$$

where *Φ* is the volume fraction; *k* is the thermal conductivity in [W m^−1^ K^−1^]; and the subscripts “s”, “m”, “w”, and “g” denote the intrinsic properties of the membrane, solid (e.g., Pt/C particles), water, and gas, respectively.

Rowe and Li [226] calculated the effective thermal conductivity of the CLs based on the membrane, solid catalyst, and liquid water phases by assuming that heat conduction occurs in parallel in each phase. The effective thermal conductivity of the CLs is estimated to be 1.6 W m^−1^ K^−1^.

56 $${k}_{\text{th}}^{\text{eff}}= {{{\varPhi} }_{\text{m}}{k}_{\text{m}}+{\varPhi} }_{\text{s}}{k}_{\text{s}}+{{\varPhi} }_{\text{w}}{k}_{\text{w}}$$

Wu et al. [227] further introduced the gas phase to calculate the effective thermal conductivity in the CL region as follows.

57 $${k}_{\mathrm{th}}^{\mathrm{eff}}= {{\varPhi} }_{\mathrm{m}}{k}_{\text{m}}+{{\varPhi} }_{\mathrm{s}}{k}_{\text{s}}+{{\varPhi} }_{\mathrm{w}}{k}_{\mathrm{w}}+{{\varPhi} }_{\mathrm{g}}{k}_{\mathrm{g}}$$

Weber and Newman [228] introduced the Bruggeman correction to the parallel phases to calculate the effective thermal conductivity of a mixture:

58 $${k}_{\mathrm{th}}^{\mathrm{eff}}= \sum_{i}{{\varPhi} }_{i}^{1.5}{k}_{{i}}$$

where the subscript “*i*” denotes the *i*th phase.

Pant et al. [229] estimated the hydration effect on the thermal conductivity of the membrane phase by assuming parallel heat transfer with Bruggeman corrections for membrane water and bulk membrane as follows:

59 $${k}_{\mathrm{m},\mathrm{wet}}= {{\varPhi} }_{\mathrm{mw}}^{1.5}{k}_{\text{w}}+{\left({1-{\varPhi} }_{\mathrm{mw}}\right)}^{1.5}{k}_{\mathrm{m},\mathrm{dry}}$$

where the subscript “mw” denotes dissolved water (or membrane water) in the wet ionomers; and *k*_m,wet_ and *k*_m,dry_ denote the thermal conductivities of wet and dry membranes, respectively.

Although rarely used in the literature, the effective thermal conductivity of CLs can be calculated as follows, with the assumption that all phases are in series.

60 $$ k_{{{\text{th}}}}^{{{\text{eff}}}} = \frac{1}{{\mathop \sum \nolimits_{i} \frac{{{\varPhi}_{{{i}}} }}{{k_{{{i}}} }}}} $$

However, the actual CL structure is more complex, and Gurau et al. [230] proposed a model based on fluid-filled spherical inclusions as follows:

61 $$ k_{{{\text{th}}}}^{{{\text{eff}}}} = - 2k_{{\text{s}}} + \frac{1}{{\frac{\varepsilon }{{2k_{{\text{s}}} + k_{{\text{g}}} }} + \frac{1 - \varepsilon }{{3k_{{\text{s}}} }}}} $$

where *ε* is the porosity.

As can be seen, many models have been developed to estimate the effective thermal conductivity of the CLs in PEM fuel cells. However, it is worth further investigating the suitability of each model based on a sufficient large experimental database for CLs.

### Summary of Structure-Dependent Physicochemical Properties

Many advanced experimental methods are developed to investigate the physicochemical properties of CLs, including the effective diffusion coefficient, permeability, capillary pressure, contact angle, and effective thermal conductivity. It should be noted that these physicochemical properties are the key parameters to study the transport phenomena and mechanical behavior of the CLs, and some other parameters, more related to the single phase of ionomer or catalyst materials, are not included in this article, including the tensile strength, thermal expansion and swelling coefficient, water hydraulic permeability, and diffusion coefficient of water in the ionomer. The connection between the effective physicochemical properties and structural parameters has also been investigated in this section. With the experimental data, many structure-based models have been developed and validated (or partially validated) to estimate these properties based on the porosity, PSD, surface area, or other structural parameters, which lay a foundation for the theory and model development of PEM fuel cells.

## Electrochemical Properties of Catalyst Layers

The PEM fuel cell performance can be determined by various electrochemical properties of CLs, such as the exchange current density, ECSA, electrode roughness factor, charge transfer coefficient, effective electronic conductivity, and effective protonic conductivity. These electrochemical properties rely heavily on not only the nature of the used materials (catalysts and ionomer) but also the structure of the CLs. In this section, the fundamentals and basic concepts of the key electrochemical properties are reviewed, and advanced experimental techniques for each parameter are comprehensively examined. Besides, the effects of the CL structure on these parameters are also scrutinized based on both theoretical and experimental analyses in this section.

### Exchange Current Density

#### Definition

At the small over-potential region, the electrochemical reaction rate is governed by the activation energy barrier that should be overcome. The speed of electrochemical reactions is reflected by how fast the electrons are liberated or consumed. This enables the direct measurement of electrochemical reaction rate from the current density according to the Faraday’s law [231]:

62 $$j=nF{J}_{\mathrm{n}}$$

where *j* is current density in [A m^−2^], *n* is the number of electrons generated or consumed per molecule of reactants, *F* is Faraday’s constant in [C kmol^−1^], and *J*_n_ is the molar flux of reactants consumed by the reaction in [kmol m^−2^ s^−1^].

The exchange current density is defined as the reaction rate at equilibrium states, where the net current in a PEM fuel cell is zero as the reversible electrochemical reaction occurs in both forward and backward directions at identical rates [231, 232]. A higher exchange current density means a more electrochemically active catalyst surface with a lower activation energy barrier, leading to a larger current at a constant over-potential. Generally, at the anode of PEM fuel cells with hydrogen as fuels, the exchange current density is much larger than that on the cathode with oxygen as oxidants.

The most commonly employed electrode kinetics in fuel cell modeling is the so-called Butler–Volmer equation in a form as shown below:

63 $$j={j}_{0}\left[{\text{exp}}\left(\frac{{\alpha }_{\text{a}}nF}{{R}_{\mathrm{u}}T}{\eta }_{\text{act}}\right)-{\text{exp}}\left(-\frac{{\alpha }_{\text{c}}nF}{{R}_{\mathrm{u}}T}{\eta }_{\text{act}}\right)\right]$$

where *j*_0_ is the exchange current density in [A m^−2^], *α* is the charge transfer coefficient, *F* is Faraday’s constant in [C kmol^−1^], and *η*_act_ is the activation over-potential in [V]. It should be noted that the definitions of the transfer coefficient significantly vary in different studies, e.g., the product of *αn* can be also used as transfer coefficients [233]. In addition, there exist various electrode kinetics models derived from the Butler–Volmer equation including Springer et al.’s [234] and Um et al.’s [235] models.

Despite the discrepancy in the different electrode kinetics models, the exchange current density is dependent on the reactant and product concentrations, temperature, catalyst loading, surface area, types of catalysts, and microstructure of the catalyst surface. In fuel cell modeling, a reference exchange current density at a specific temperature and pressure is often given, and the effective exchange current density can be estimated by the following equation at different conditions [227]:

64 $${j}_{0}={j}_{0}^{\mathrm{ref}}{r}_{\mathrm{f}}{\left(\frac{{c}_{\mathrm{r}}}{{c}_{\mathrm{ref}}}\right)}^{\gamma }\mathrm{exp}\left[-\frac{{E}_{\mathrm{act}}}{{R}_{\mathrm{u}}T}\left(1-\frac{T}{{T}_{\mathrm{ref}}}\right)\right]$$

where *j*_0_^ref^ is the reference exchange current density at the given temperature and pressure per unit catalyst surface area in [A cm_Pt_^−2^]; *r*_f_ is the electrode roughness factor; *c*_r_ and *c*_ref_ are the actual and reference reactant concentrations in [kmol m^−3^], respectively; *γ* is the pressure dependency coefficient, or reaction order (ranging from 0.5 for HOR to 1.0 for ORR); and *E*_act_ is the activation energy in [kJ mol^−1^] (*E*_act,a_ = 12 kJ mol^−1^ for HOR and *E*_act,c_ = 66 kJ mol^−1^ for ORR [205]). It should be noted that in some studies [231], the concentration ratio is substituted with a pressure ratio.

#### Experimental Methods for Exchange Current Density

The availability of experimental data on the exchange current density for PEM fuel cells is limited. In certain circumstances (large activation over-potentials), the Butler–Volmer equation can be simplified in a Tafel relation.

65 $$j={j}_{0}\mathrm{exp}\left(\frac{\alpha nF{\eta }_{\text{act}}}{{R}_{\mathrm{u}}T}\right)$$

The Tafel equation is first observed from experimental data for the relation between the voltage drop and current density, and can be written as follows.

66 $${\eta }_{\text{act}}=\frac{{R}_{\mathrm{u}}T}{\alpha nF}\mathrm{ln}\left(\frac{j}{{j}_{0}}\right)$$

**Fig. 22:**
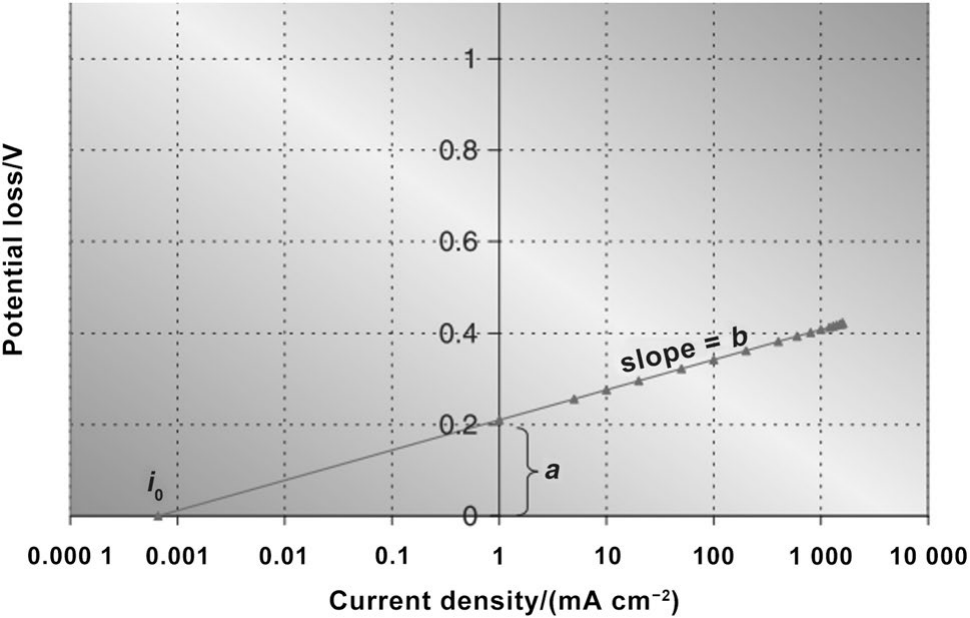
Tafel plot of cell potential loss against current density. Reprinted with permission from Ref. [231]. Copyright © 2005, Elsevier

This expression can be re-written as follows:

67 $${\eta }_{\text{act}}=a+b\mathrm{log }j$$

where the coefficients *a* and *b* can be determined through experimental data by curve fitting as shown in Fig. 22. *a* can be read from the intercept of the Tafel plot, and *b* can be determined by the slope of the Tafel plot. The coefficients *a* and *b* can be expressed as follows.

68 $$a=-\mathrm{ln}(10)\frac{{R}_{\mathrm{u}}T}{\alpha nF}\mathrm{log }{j}_{0}$$

69 $$b=\mathrm{ln}(10)\frac{{R}_{\mathrm{u}}T}{\alpha nF}$$

Therefore, the exchange current density *j*_0_ and the charge transfer coefficient α can be found from Tafel plots.

Li and Pickup [236] experimentally determined the exchange current by a Tafel analysis of the overall cell polarization curve using the following equation:

70 $$E={E}_{0}-b\mathrm{log }j-Rj$$

where *R* is an approximation of the ohmic resistance dominating the linear voltage drop region, and the constant *E*_0_ is expressed as

71 $${E}_{0}={E}_{\mathrm{eq}}+b\mathrm{log}{ j}_{0}$$

where *E*_eq_ is the theoretical equilibrium potential [1.2 V in respect to the reference hydrogen electrode (RHE)]. The exchange current density of the cathode CLs under the different Nafion loadings of 0.3–1.5 mg cm^−2^ (Pt loadings of 0.4 mg cm^−2^) is 0.74×10^−3^–2.7×10^−3^ A m^−2^ (see Table 12 for more details). Haghayegh et al. [237] determined the exchange current density of two different MEAs by fitting a 3D model to published experimental data. However, the details about the procedure to determine the exchange current density are not presented, making it difficult to justify its accuracy and suitability.

**Table 12:** Exchange current density of the catalyst layers in PEM fuel cells

Author	Reference exchange current density/(A m^−2^)	CL thickness/μm	Composition and condition	Experimental or for modeling	Ref.
Li and Pickup	Cathode: 0.74 × 10^−3^	–	20% Pt/C, 0.4 mg_Pt_ cm^−2^, 0.3 mg cm^−2^ Nafion	Experimental	[236]
Li and Pickup	Cathode: 1.9 × 10^−3^	–	20% Pt/C, 0.4 mg_Pt_ cm^−2^, 0.6 mg cm^−2^ Nafion	Experimental	[236]
Li and Pickup	Cathode: 1.5 × 10^−3^	–	20% Pt/C, 0.4 mg_Pt_ cm^−2^, 0.9 mg cm^−2^ Nafion	Experimental	[236]
Li and Pickup	Cathode: 1.1 × 10^−3^	–	20% Pt/C, 0.4 mg_Pt_ cm^−2^, 1.2 mg cm^−2^ Nafion	Experimental	[236]
Li and Pickup	Cathode: 2.7 × 10^−3^	–	20% Pt/C, 0.4 mg_Pt_ cm^−2^, 1.5 mg cm^−2^ Nafion	Experimental	[236]
Haghayegh et al	Anode: 9.2 × 10^2^ Cathode: 9.2 × 10^−8^	50	17.23% Pt/multiwalled carbon nanotube, 0.4 mg_Pt_ cm^−2^, PTFE, ionomer, *T*_ref_ = 333.15 K	Curve fitting from experimental data	[237]
Haghayegh et al	Anode: 1.5 × 10^3^ Cathode: 1.5 × 10^−7^	50	17.23% Pt/multiwalled carbon nanotube, 0.4 mg_Pt_ cm^−2^, PTFE, ionomer, *T*_ref_ = 353.15 K	Curve fitting from experimental data	[237]
Haghayegh et al	Anode: 6 × 10^2^ Cathode: 5 × 10^−8^	50	8.27% Pt/multiwalled carbon nanotube, 0.4 mg_Pt_ cm^−2^, PTFE, ionomer	For modeling	[237]
Haghayegh et al	Anode: 1.0 × 10^3^ Cathode: 1.0 × 10^−7^	50	17.23% Pt/multiwalled carbon nanotube, 0.4 mg_Pt_ cm^−2^, PTFE, ionomer	For modeling	[237]
Rowe and Li	Anode: 4.0 × 10^4^ Cathode: 1.3 × 10^−2^	10	–	For modeling	[226]^a^
Ye and Nguyen	Anode: 3 × 10^3^ Cathode: 1.5 × 10^−1^	15	*T*_ref_ = 343 K	For modeling	[205]^a^
Goshtasbi	Anode: 3.0 × 10^3^ Cathode: 3.0 × 10^−2^	8	*T*_ref_ = 303 K	For modeling	[238]
Jiang et al	Anode: 3.0 × 10^3^ Cathode: 1.2 × 10^−2^	10	–	For modeling	[239]
Li et al	Anode: 1.0 × 10^4^ Cathode: 1.0 × 10^1^	15	–	For modeling	[195]

^a^Denotes data corrected from [A m^−3^] to [A m^−2^] based on the thickness of catalyst layers

As only limited experimental data are available in the literature, the reference exchange current density used in various modeling studies is summarized in Table 12. As can be seen that the cathodic and anodic exchange current density used for modeling varies significantly from 10^2^ to 10^4^ A m^−2^ for HOR and 10^−8^ to 10^1^ A m^−2^ for ORR in various studies. The considerable discrepancy of the exchange current density in various studies necessitates further experimental measurements.

### Charge Transfer Coefficient

#### Definition

The charge transfer coefficient is an important parameter related to the kinetics of the electrochemical reactions, which is used in Butler–Volmer and other related equations [234, 235, 240]. The definition of charge transfer coefficients according to IUPAC in 1981 [240] is shown below.

For cathode:

72 $$ \frac{{\alpha_{{\text{c}}} }}{\nu } = - \frac{{R_{{\text{u}}} T}}{nF}\left( {\frac{{\partial {\text{ln}}\left| {I_{{{\text{red}}}} } \right|}}{\partial E}} \right)_{{p,T,c_{i}^{{{\text{interface}}}} }} $$

For anode:

73 $$ \frac{{\alpha_{{\text{a}}} }}{\nu } = \frac{{R_{{\text{u}}} T}}{nF}\left( {\frac{{\partial {\text{ln}}\left| {I_{{{\text{ox}}}} } \right|}}{\partial E}} \right)_{{p,T,c_{i}^{{{\text{interface}}}} }} $$

where *ν* is the stoichiometric number. $\frac{{\alpha }_{\mathrm{c}}}{\nu }$ and $\frac{{\alpha }_{\mathrm{a}}}{\nu }$ can be considered as the observable transfer coefficients for cathodic and anodic reactions, respectively.

A more recent recommendation from IUPAC in 2014 [233] modified the expression of the transfer coefficients as follows.

For cathode:

74 $${\mathrm{\alpha }}_{\mathrm{c}}=-\frac{{R}_{\mathrm{u}}T}{F}\frac{\mathrm{d}\left(\mathrm{ln }{j}_{\mathrm{a}}\right)}{\mathrm{d}E}$$

For anode:

75 $${\alpha }_{\mathrm{a}}=\frac{{R}_{\mathrm{u}}T}{F}\frac{\mathrm{d}\left(\mathrm{ln }{j}_{\mathrm{c}}\right)}{\mathrm{d}E}$$

However, various forms and values of transfer coefficients are reported in different fuel cell studies, and these values should be carefully investigated when different types of models are employed.

#### Experimental Methods for Charge Transfer Coefficient

Similar to the reference exchange current density, only limited experimental methods are reported for the measurement of the transfer coefficient. Generally, the transfer coefficient can be measured by fitting the Tafel equation to the voltage–current relation [231], and the value of the transfer coefficient can be calculated by Eq. (69). However, experimental measurements of the transfer coefficient for anodic and cathodic reactions in PEM fuel cells are very rare. It will be interesting to accurately measure these electrochemical coefficients to further improve the development of the PEM fuel cell model and theory. Table 13 summarizes the charge transfer coefficients used for CL modeling, and the reported values are divergent. The effect of charge transfer coefficient on the modeling performance remains unclear.

**Table 13:** Charge transfer coefficient used for modeling catalyst layers

Author	*α*_a_	*α*_c_	*α*_a_*n*_a_	*α*_c_*n*_c_	Ref.
Springer et al.	–	–	–	0.5	[234]
Kulikovsky et al.	–	–	–	2	[241]
Rowe and Li	–	–	1	1	[226]
Ye and Nguyen	–	–	1	1	[205]
Le and Zhou	–	–	0.5	0.5	[242]
Ismail et al.	–	–	0.5	0.512	[187]
Haghayegh et al.	0.5	0.5	–	–	[237]
Goshtasbi et al.	–	–	2.0	0.5	[238]
Jiang et al.	0.5	0.5	–	–	[239]
Li et al.	–	–	1.0	1.0	[195]

Assuming *n*_a_ = 2 for anodic hydrogen oxidation reaction and *n*_c_ = 4 for cathodic oxygen reduction reaction for a H_2_/O_2_ PEM fuel cell

### Electrochemical Surface Area and Electrode Roughness Factor

#### Definition

ECSA is a critical parameter that determines the performance of CLs in PEM fuel cells [243]. The values of ECSA are theoretically determined by the nanostructure and size of the catalysts, microstructure of the catalyst–ionomer mixture, the pore structure, and the amount of the liquid water in CLs. For Pt catalysts, ECSA is defined as the active surface area accessible to reactants during the cell operation, which is often on a per unit mass basis [92]:

76 $$a=\frac{{A}_{{\mathrm{P}}_{\mathrm{t}}}}{{m}_{{\mathrm{P}}_{\mathrm{t}}}}$$

where *a* is the ECSA in [cm^2^ mg_Pt_^−1^], *A*_Pt_ is the active surface area of the Pt catalyst in PEM fuel cells in [cm^2^], and *m*_Pt_ is the mass of the Pt catalyst in [mg].

The electrode roughness factor is defined as the active catalyst surface area per electrode geometric area [231, 244]. According to the definition, the electrode roughness factor, *r*_f_ in [m_Pt_^2^ m_geo_^−2^], can be calculated as follows,

77 $${r}_{\mathrm{f}}=\frac{{A}_{{\mathrm{P}}_{\mathrm{t}}}}{{A}_{\mathrm{geo}}}=a\frac{{m}_{{\mathrm{P}}_{\mathrm{t}}}}{{A}_{\mathrm{geo}}}=a{L}_{\mathrm{Pt}}$$

where *r*_f_ is the dimensionless electrode roughness factor, *A*_geo_ is the geometric surface area which depends on the shape and geometrical dimension of the overall electrode in [cm^2^], and *L*_Pt_ is the Pt loading in [mg_Pt_ cm^−2^]. Therefore, the ECSA and electrode roughness factor theoretically share the same experimental methods according to the definitions, and for brevity, the major experimental methods only for ECSA are reviewed in this section.

The electrode roughness factors can also be affected by liquid water coverage on the electrode surface, and a correction factor is often applied to take the liquid water saturation in CLs into account [231]:

78 $${r}_{\text{f}}={\left(1-{{\varPhi} }_{\text{l}}\right)}^{m}a{L}_{\mathrm{Pt}}$$

where ${{\varPhi} }_{\text{l}}$ is the volumetric ratio of liquid water in the CL pores to the pore volume, and *m* is the correction factor taking the liquid-occupied surface into account (${{\varPhi} }_{\text{l}}$ = 0 when no liquid water exists in CLs).

#### Experimental Methods for Electrochemical Surface Area

Many experimental methods have been employed to measure the ECSA of the catalysts in PEM fuel cells, especially for Pt catalysts. These methods include cyclic voltammetry (CV), CO stripping voltammetry, linear sweep voltammetry (LSV), and electrochemical impendence.

**Fig. 23:**
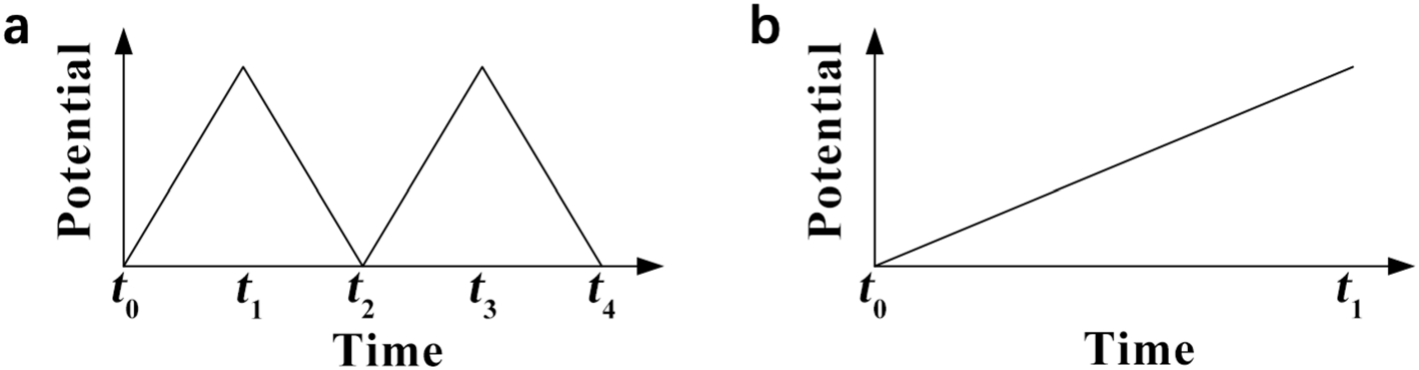
Different types of voltammetry: **a** cyclic voltammetry and **b** linear sweep voltammetry

Voltammetry is a common electrochemical technique to evaluate the catalyst performance in PEM fuel cells, which measures the resulted electric current when the electrode is subject to a sweeping voltage [86, 245, 246]. According to the shape of the sweeping voltage, the voltammetry can be classified into many categories, including CV, LSV, square-wave voltammetry, staircase voltammetry, and other voltammetry techniques. The CV and LSV are the most commonly employed methods in fuel cell studies. In CV, the applied potential ramps linearly with time between upper and lower voltage limits (see Fig. 23a), while in LSV, the voltage is linearly swept with time (see Fig. 23b).

**Fig. 24:**
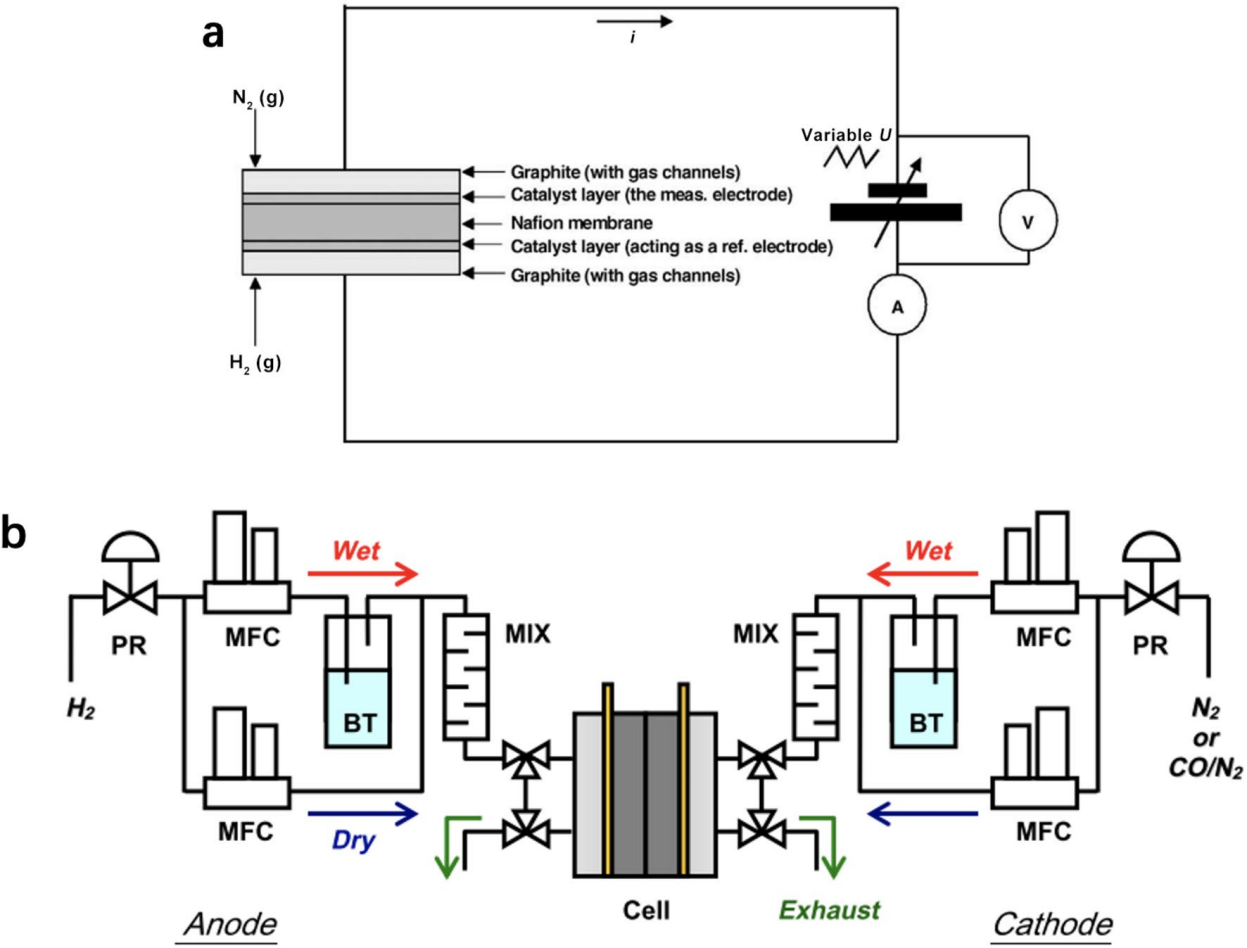
Schematic of **a** cyclic voltammetry (CV) test apparatus (reprinted with permission from Ref. [250], copyright © 2003, Elsevier) and **b** CO stripping test apparatus (reprinted with permission from Ref. [251], copyright © 2013, Elsevier)

By analyzing the CV curve (voltage vs. current) obtained from an typical CV apparatus as shown in Fig. 24a, the oxidation and reduction potentials, diffusion coefficients of species, and ECSA can be quantitatively estimated under proper assumptions [41, 86, 247]. The ECSA can be calculated from the charge required for adsorbing/desorbing a monolayer of hydrogen in the hydrogen adsorption/desorption region of a CV or for oxidizing a monolayer of CO in a stripping curve [248]. For hydrogen-based CV commonly used for fuel cell studies, the ECSA (*a*) in [cm^2^_Pt_ g_Pt_^−1^] can be calculated using the following equation [245]:

79 $$a=\frac{{Q}_{\mathrm{H}}}{{C}_{\mathrm{H}}{L}_{\mathrm{Pt}}}$$

where *Q*_H_ is the charge associated with a monolayer of hydrogen adsorption/desorption on a Pt surface in [μC cm^−2^], *C*_H_ is the charge required for the adsorption/desorption of a monolayer of hydrogen on a Pt surface, which is often assumed to be 210 μC cm_Pt_^−2^, and *L*_Pt_ is the Pt loading in [g_Pt_ cm^−2^]. *Q*_H_ can be calculated by integrating the CV for hydrogen adsorption/desorption process after a double-layer correction at low potentials (in the underpotential deposition region), which gives the number of hydrogen atoms adsorbed [248],

80 $$ Q_{{\text{H}}} = \int_{{t_{1} }}^{{t_{2} }} {I{\text{d}}t} = \frac{1}{v}\int_{{E_{1} }}^{{E_{2} }} {I{\text{d}}E} $$

where *E* is the potential in [V], *I* is the current in [A], and *v* is the sweep rate in [V s^−1^].

Park et al. [249] investigated the Pt/C catalyst degradation as a result of start-up/shutdown cycles. Their results suggested that the frequent cyclic voltammetry performed [H_2_/N_2_ gas pair at 80 °C at 100% of relative humidity (RH) conditions] during start-up/shutdown cycling has a significant impact on the catalyst degradation, and the measured ECSA reduced from the initial 61.3 to 28.8 m^2^ g_Pt_^−1^, demonstrating a 53% reduction in ECSA. Koponen et al. [250] utilized the in situ voltammograms apparatus in Fig. 24a by flushing H_2_ to the reference electrode and N_2_ to the measuring electrode by sweeping the potential between 0.05 and 0.6 V. The ECSA was measure to be 34 m^2^ g_Pt_^−1^, meaning about 30% of the Pt in the CLs were electroactive.

When a monolayer of CO already adsorbed on the catalyst surface is electrochemically oxidized and removed from the surface (see apparatus in Fig. 24b), the CO stripping curve can be obtained, and the ECSA can be calculated using the following equation [68].

81 $$ a = \frac{{Q_{{{\text{CO}}}} }}{{C_{{{\text{CO}}}} L_{{{\text{Pt}}}} }} $$

where *a* is the electrochemical surface area in [cm_Pt_^2^ g_Pt_^−1^], *Q*_CO_ is the charge of a monolayer of CO on the catalyst surface in [μC cm^−2^], and *C*_CO_ is the specific charge required to oxidize a monolayer of CO on the catalyst surface, which is often assumed to be 420 μC cm_Pt_^−2^.

Saha et al. [68] performed CO stripping measurements on several commercially available gas diffusion electrodes by purging CO and maintaining the potential at 0.05 V versus RHE for 1 h at 25 °C. The measured ECSA varies from 22.3 to 39.7 m^2^ g_Pt_^−1^, which is consistent with the hydrogen CV results (18.0–36.3 m^2^ g_Pt_^−1^). Iden and Ohma [251] studied the dependence of RH on the ECSA of a graphitized-Ketjen-black-based CL by CO stripping, and found that the ECSA ranged from 33 to 40 m^2^ g_Pt_^−1^. Their results indicated that ECSA measured by CO stripping may be overestimated due to the complex microstructure of the samples and the RH conditions.

Reid et al. [252] derived an expression for ECSA estimation by correlating Faradaic pseudo-capacitance determined by the method of electrochemical impendence spectroscopy (EIS) with ECSA determined by CV. It is found that the decay profile for both ECSA and the Faradaic pseudo-capacitance is almost identical, which allows the derivation of an empirical correlation between the EIS and CV methods. Therefore, the ECSA can be estimated directly from the EIS results without performing a CV test. However, strict validation and careful calibration of EIS data on the ECSA values are required.

### Effective Electronic Conductivity

#### Ohm’s Law

The transport of electrons is governed by Ohm’s law in electron-conductive components in the PEM fuel cell, such as bipolar plates, GDLs, MPLs, and Pt/C network in CLs [160, 182, 253]. The Ohm’s law suggests that the current through a conductor between two points is proportional to the voltage difference between these two points:

82 $$I=\frac{U}{R}=\frac{{k}_{\mathrm{ele}}AU}{l}$$

where *I* is current in [A], *U* is the voltage in [V], *R* is resistance in [Ω], *k*_ele_ is the electronic conductivity in [S m^−1^], *A* is the cross-sectional area [m^2^], and *l* is the distance between the two points in [m].

For the porous CLs, the Ohm’s law is modified as follows,

83 $$I=\frac{{k}_{\mathrm{ele}}^{\text{eff}}AU}{l}$$

where *k*_ele_^eff^ is the effective electronic conductivity of the porous media in [S m^−1^], which is affected by the CL composition and its microstructure. The values of effective electronic conductivity are often estimated by experimental approaches, and many correlation models have been developed based on experimental data.

#### Experimental Methods for Effective Electronic Conductivity

**Fig. 25:**
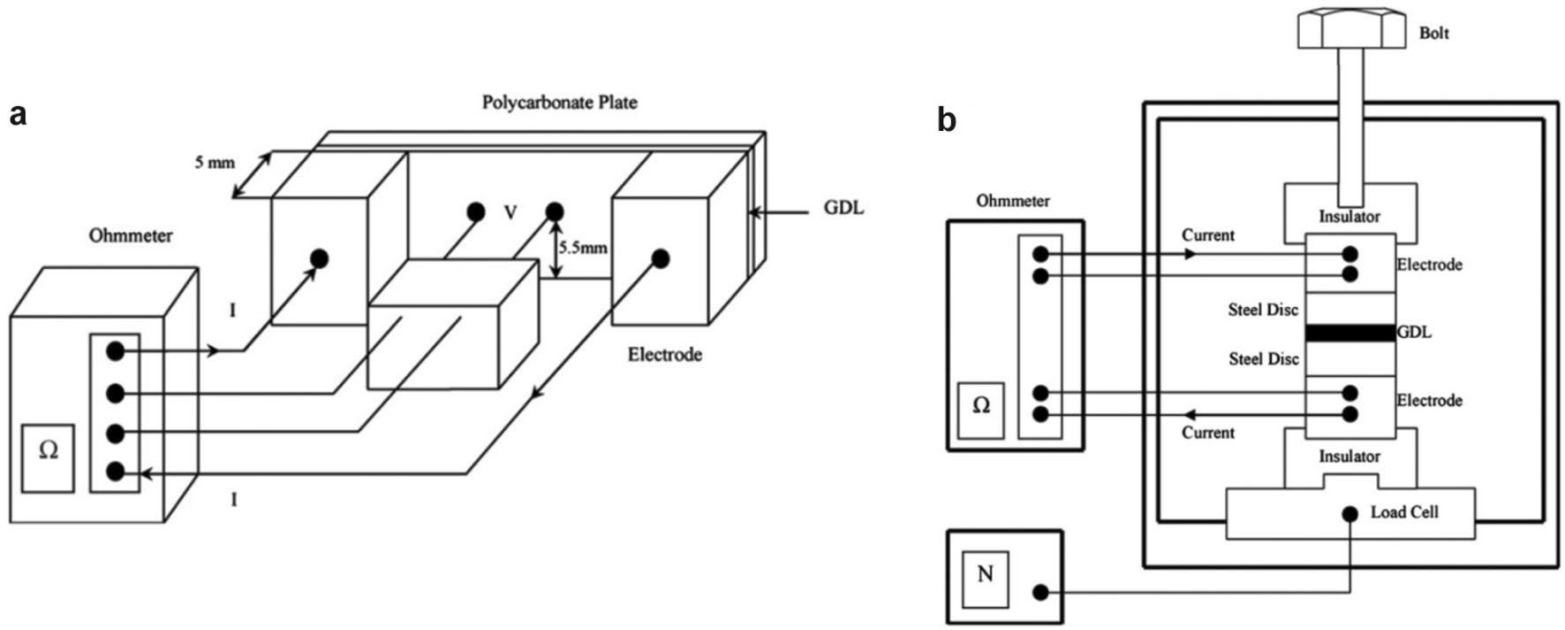
Schematic of the four-probe technique to measure the effective electrical conductivity for **a** in-plane and **b** through-plane directions. Reprinted with permission from Ref. [254]. Copyright © 2010, Elsevier

Many experimental methods have been developed for the measurement of effective electronic conductivity of porous media in PEM fuel cells. Ismail et al. [254] developed two experimental apparatuses to measure the in-plane and through-plane effective electronic conductivities of porous media in PEM fuel cells using the four-probe methods. For the in-plane effective electronic conductivity measurement, the test sample is prepared in squared shape and placed on an insulating polycarbonate plate, as shown in Fig. 25a. Current is provided by two copper electrodes passing through the test specimen, and the voltage between two selected points located in the middle of the two copper electrodes is measured by two gold-plated probes. By Ohm’s law, the effective electronic conductivity between the two points can be determined as follows,

84 $${k}_{\mathrm{ele}}^{\text{eff}}=\frac{1}{C\delta R}$$

where *C* is the geometric correction factor, which is determined by the specimen dimension and the space between the probes, and should be carefully taken into account for uncertainty analysis; *δ* is the thickness of the test specimen in [m], and *R* is the electronic resistance in [Ω]. For the through-plane conductivity measurement, the sample is prepared in a circular shape and placed between two stainless steel disks, which are compacted by two copper electrodes. The total resistance of the assembly is measured at different compaction levels, and based on the resistance network theory, the effective electronic conductivity of the specimen can be obtained with contact resistance included. Only GDLs are tested by using these instruments, and it can be further modified to test the CLs with improved measurement uncertainty.

Tranter et al. [255] measured the in-plane effective electronic conductivity of different CLs prepared with an alternative four-point probe technique (a.k.a. van der Pauw technique [256]), where the four probes are placed at the periphery of the test specimen. The CLs under investigation is prepared using ionomer and Pt/C with different composition, thickness, and milling time and temperature. The resulted effective electronic conductivity is found to be between 122 and 605 S m^−1^. They pointed out that the four-point probe method is not suitable for through-plane effective electronic conductivity measurement of the CL as it is too thin for the placement of the probes.

Sadeghifar [257] measured the in-plane conductivity of the CCM using a two-thickness method by testing two different samples with different lengths. The experimental results indicate that the in-plane effective electronic conductivity of wet CCM is about 70 S m^−1^, which is about three times lower than that of the dry CCM. More details of the experimental data on effective electronic conductivity of CLs can be found in Table 14.

**Table 14:** Effective electronic conductivity of catalyst layers from experimental results

Catalyst layer composition	Direction	Thickness/µm	Effective electronic conductivity/(S m^−1^)	Ref.
Ionomer-carbon ratio = 1.1, 50% Pt/C, coated on PTFE, dry-milling time = 0 h @ 20 °C	In-plane	7.77 ± 0.43	605 ± 23	[255]
Ionomer-carbon ratio = 1.1, 50% Pt/C, coated on PTFE, dry-milling time = 48 h @ 20 °C	In-plane	4.41 ± 0.93	122 ± 23	[255]
Wet CL coated on membrane, 50% porosity	In-plane	15	70	[257]
Dry CL coated on membrane, 50% porosity	In-plane	15	210	[257]
Ionomer, Pt/C, CCM, hot pressing, volume fraction of Pt/C = 0.6	In-plane	~10	30/110	[258]
Ionomer, Pt/C, CCM, hot pressing, volume fraction of Pt/C = 0.665	In-plane	~10	10/105	[258]
Ionomer, Pt/C, CCM, hot pressing, volume fraction of Pt/C = 0.75	In-plane	~10	12/13	[258]
Ionomer, Pt/C, CCM, hot pressing, volume fraction of Pt/C = 0.8	In-plane	~10	12.1	[258]

#### Empirical Models for Effective Electronic Conductivity

The effective electronic conductivity of the CL is primarily determined by its composition and the corresponding microstructure. Many correlation models are established based on the porosity and the volumetric ratio of electronic conductive components.

Das et al. [180] derived a correlation model for the effective electronic conductivity of the CLs as follows:

85 $$ k_{{{\text{ele}}}}^{{{\text{eff}}}} = k_{{\text{s}}} \left\{ {1 - x\left[ {\frac{{3\left( {1 - {{\varPhi}}_{{\text{s}}} } \right)}}{{3 - {\varPhi}_{{\text{s}}} }}} \right]} \right\} $$

where *k*_s_ is the bulk electronic conductivity of the solid phase in CLs, *x* is the solid phase geometry factor, and *Φ*_*s*_ is the volume fraction of the solid phase in CLs.

Zhao and Li [4] applied a Bruggeman correction to the CLs taking the porosity and ionomer volume into account

86 $${k}_{\text{ele}}^{\text{eff}}={k}_{\text{s}}{(1-\varepsilon -{{\varPhi} }_\text{m})}^{1.5}$$

where *ε* is porosity, and *Φ*_m_ is the volume fraction of the ionomer in CLs.

Although these correlations have been broadly used in fuel cell modeling, their accuracy and suitability are worth further investigation based on a sufficiently large experimental dataset.

### Effective Protonic Conductivity

#### Ohm’s Law

The transport of protons is governed by Ohm’s law in proton-conductive components, such as membrane and ionomers in CLs [160, 182]. In the membrane, the protonic conductivity is determined by the materials, temperature, and water content. In CLs, the effective protonic conductivity is often used to evaluate the capability of the CLs to transport protons through the ionomer networks, which can be affected by the volumetric fraction of ionomer, the amount of liquid water, porosity, and other structural parameters.

#### Experimental Methods for Effective Protonic Conductivity

Many methods have been developed to investigate the protonic conductivity of membrane, including direct current (DC) scanning or alternating current (AC) impedance [259–261], while the direct measurement of the effective protonic conductivity of the CLs is very scarce.

**Fig. 26:**
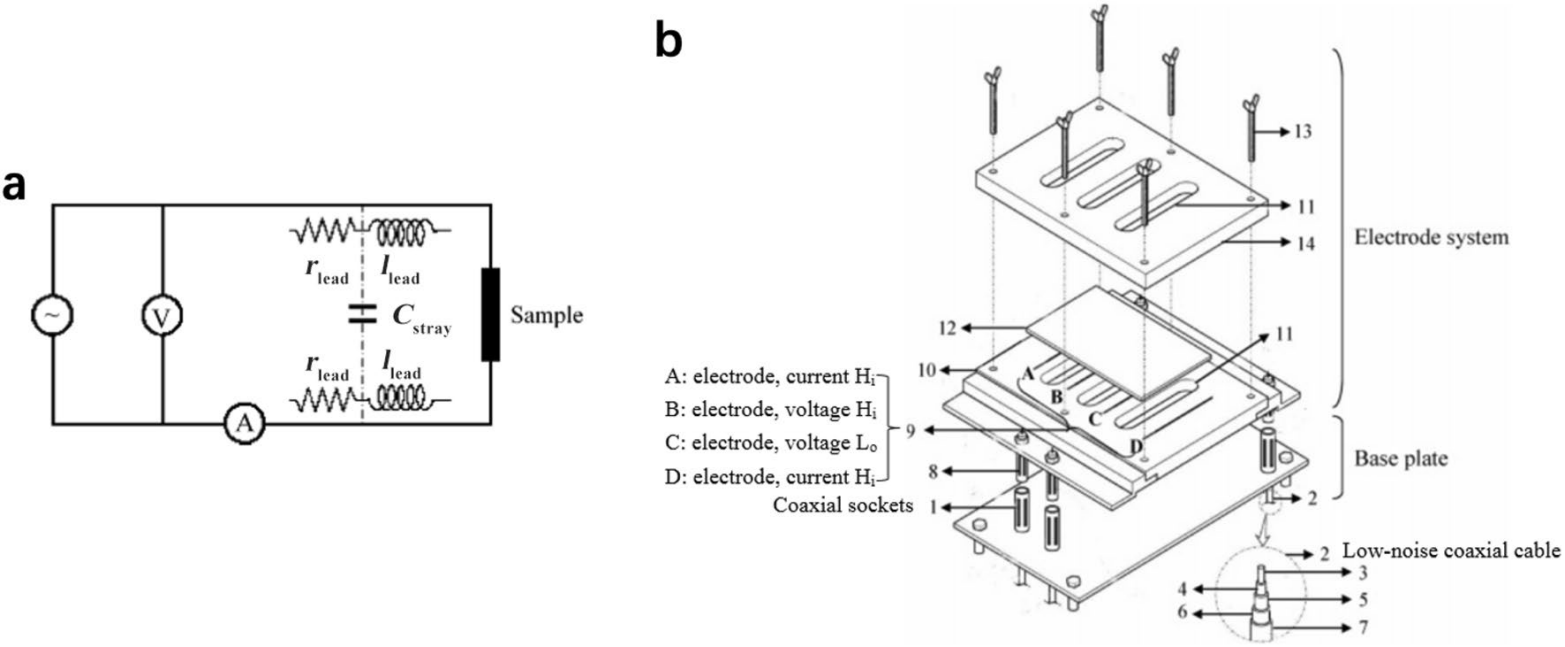
Schematic of impedance measurement systems: **a** two-probe method and **b** four-probe method. Adapted with permission from Ref. [262]. Copyright © 2005, American Chemical Society

Lee et al. [262] measured the protonic conductivity of the membrane using an impedance measurement system based on two- or four-probe methods in water vapor or liquid water, as shown in Fig. 26. The impedance of the Nafion membranes is measured by applying an AC amplitude of 1 mA over the AC frequency from 0.1 Hz to 100 kHz at controllable humidity and temperature levels. From the Nyquist plot, the real (*Z*′) and imaginary components (*Z*′′) of impedance for the membrane are recorded, and the intercept of the *Z*′-axis is approximately the ohmic resistance of the membrane. From the Bode plot, the change of impedance over a broad range of frequency yields the ohmic resistance of the test specimen. Finally, the protonic conductivity of membrane, *k*_ion_, can be derived from the following equation

87 $$ k_{{{\text{ion}}}} = \frac{l}{AR} $$

where *l* is the distance between the reference electrodes in [m], *A* is the cross-sectional area of the membrane in [m^2^], and *R* is the bulk resistance of the membrane obtained from the impedance analyzer in [Ω]. The experimental results suggest that the protonic conductivity of the membrane measured by the four-probe method is always 2–5 times higher than those by two-probe methods at ambient conditions.

Li and Pickup [236] investigated the effective protonic conductivity of the CLs using EIS by feeding dry nitrogen to cathode immediately after the hydrogen–oxygen polarization measurement. The dynamic impedance responses indicate that the effective protonic conductivity of the cathode rises with ionomer content. The ionic conductivity of the CLs with various Nafion contents ranges from 0.17 to 1.1 S m^−1^. As many factors can affect the accuracy of the experiment, the measurement uncertainty should be assessed. It should be mentioned that the EIS has been a primary technique used for MEA structure optimization, ionic conductivity measurement, and fuel cell diagnostics. It can also help determine other contributors to the impedance of the whole fuel cell stack, including interfacial charge transfer resistance, protonic resistance, contact resistance, mass transport resistance, and double-layer capacitance. However, the accuracy of EIS measurement depends on many factors, including the accuracy and precision of the instruments, operating procedures, and EIS data interpretation [263, 264]. In this review, emphasis has been placed on the individual CL component, and more details of the EIS methods can be found elsewhere [263–265].

Boyer et al. [266] experimentally investigated the effective protonic resistance of the ionomer networks by introducing an inert layer of ionomer and carbon particles in the MEA with varied carbon loadings. The ohmic resistance is estimated by fitting Eq. (70) to the low current density regions of the polarization curve (5–800 mA cm^−2^). It should be noted that the resistance estimated from the polarization curve includes the resistance resulting from the membrane, electrode, electron-conductive components, contact interface, mass transfer, and most importantly the inactive layer. As the cell components and test apparatus are identical except for the inactive layer, it is assumed that the difference in ohmic resistance with varied thickness of inactive layers is solely due to the inactive layer. The resistance variation resulting from the inactive layers with various carbon loadings can be thus used to determine the effective protonic conductivity of the ionomer networks in the inactive layers. The experimental results demonstrate a protonic conductivity of 0.013 S cm^−1^ for the mixture of Nafion ionomer (33 wt%) and carbon black with a thickness of 20–25 µm and 0.018 S cm^−1^ with 60 wt% ionomer mixed with carbon black. However, it should be noted that the measurement uncertainty caused by the electronic resistance of the carbon networks in the active layers is not evaluated in the studies.

#### Empirical Models for Effective Protonic Conductivity

The effective protonic conductivity of CLs is determined by its ionomer and water content and microstructure—the ionomer and water content determines the intrinsic protonic conductivity of ionomer, while the microstructure affects the overall effective conductivity. Many correlation models have been developed to estimate the intrinsic protonic conductivity of ionomers and effective protonic conductivity of CLs, as summarized in Table 15. Springer et al. [234] established an empirical correlation of protonic conductivity based on Nafion 117 membranes as a function of water content and temperature, which is widely used for fuel cell modeling even though different types of membranes are employed. Sone et al. [267] proposed another polynomial empirical equation about the protonic conductivity of Nafion 117 based on experimental studies at a different temperature between 293 and 343 K with relative humidity levels between 40% and 100%. Boyer et al. [266] built an empirical correlation based on experimental data obtained from polarization curves and found that the ohmic resistance of CLs shows a linear relation with the volumetric fraction of ionomers in the CLs. Boyer et al. [266] also suggested that the effective protonic conductivity of the CLs and the bulk protonic conductivity of the membranes are in exponential relation to the power of 1.2–4.5. Das et al. [180] derived a model to calculate the effective protonic conductivity of the CLs based on the volumetric ratio of ionomer and void space based on the assumption that the CL is composed of multiple spherical catalyst particles covered by thin uniform ionomer layers. In this study, the derived model was compared with Bruggeman correlation (also proposed by Das et al. [180]) under different membrane-catalyst ratios, and the comparison demonstrated good agreements. It should be noted that limited experimental data on effective protonic conductivity of CLs are available in literature due to the difficulty in experimental measurements. Therefore, the validity of various empirical and analytical models should be further explored.

**Table 15:** Correlations for the protonic conductivity of the catalyst layers and membranes in PEM fuel cells

Model	Formula	Remark	Component	Eq.	Ref.
Springer et al.	$k_{{\text{m}}} = \left( {0.005\,139\lambda - 0.003\,26} \right){\text{exp}}\left[ {1\,268\left( {\frac{1}{303} - \frac{1}{T}} \right)} \right]$	*λ* > 1	Membrane	(88)	[234]
Sone et al.	$$\begin{aligned} k_{{\text{m}}} = & - 19.8 \times 10^{ - 3} + 16.6 \times 10^{ - 4} x \\ & - 34.5 \times 10^{ - 6} x^{2} + 28.4 \times 10^{ - 8} x^{3} \\ \end{aligned}$$	293 K	Membrane	(89)	[267]
Sone et al.	$$\begin{aligned} k_{{\text{m}}} = & - 8.01 \times 10^{ - 3} + 6.72 \times 10^{ - 4} x \\ & - 11.6 \times 10^{ - 6} x^{2} + 11.8 \times 10^{ - 8} x^{3} \\ \end{aligned}$$	303 K	Membrane	(90)	[267]
Sone et al.	$$\begin{aligned} k_{{\text{m}}} = & - 1.75 \times 10^{ - 3} + 1.45 \times 10^{ - 4} x \\ & + 0.016\,1 \times 10^{ - 6} x^{2} + 3.45 \times 10^{ - 8} x^{3} \\ \end{aligned}$$	318 K	Membrane	(91)	[267]
Sone et al.	$$\begin{aligned} k_{{\text{m}}} = & - 3.41 \times 10^{ - 3} + 2.73 \times 10^{ - 4} x \\ & - 2.67 \times 10^{ - 6} x^{2} + 5.72 \times 10^{ - 8} x^{3} \\ \end{aligned}$$	333 K	Membrane	(92)	[267]
Sone et al.	$$\begin{aligned} k_{{\text{m}}} = & - 1.56 \times 10^{ - 3} + 1.21 \times 10^{ - 4} x \\ & + 1.01 \times 10^{ - 6} x^{2} + 3.95 \times 10^{ - 8} x^{3} \\ \end{aligned}$$	343 K	Membrane	(93)	[267]
Boyer et al.	${k}_{\text{m}}^{\text{eff}}=0.078{{\varPhi} }_{\text{m}}+0.004$	Experimental correlation	Catalyst layer	(94)	[266]
Boyer et al.	$\frac{{k}_{\text{m}}^{\text{eff}}}{{k}_{\text{m}}}={{{\varPhi} }_{\text{m}}}^{n}$	*n* = 1.2–4.5	Catalyst layer	(95)	[266]
Das et al.	$$\begin{aligned} \frac{{k_{{\text{m}}}^{{{\text{eff}}}} }}{{k_{{\text{m}}} }} = & 1 \\ \, & - \beta \left\{ {\frac{{3\left( {1 - {\varPhi}_{{\text{m}}} } \right)}}{{3 - {\varPhi}_{{\text{m}}} }} + \frac{{3\varepsilon \left[ {1 - \frac{{3\left( {1 - {\varPhi}_{{\text{m}}} } \right)}}{{3 - {\varPhi}_{{\text{m}}} }}} \right]}}{2 + \varepsilon }} \right\} \\ \end{aligned}$$	Spherical catalyst particles covered by ionomers	Catalyst layer	(96)	[180]
Das et al.	$\frac{{k}_{\text{m}}^{\text{eff}}}{{k}_{\text{m}}}={\left[{{\varPhi} }_{m}\left(1-\varepsilon \right)\right]}^{1.5}$	Bruggeman correlation	Catalyst layer	(97)	[180]

${\varPhi }_{\text{m}}$ is the volume fraction of the Nafion in CLs; ${\lambda }_{\text{m}}$ is correction factor; *k*_m_ is bulk membrane protonic conductivity in [S cm^−1^]; *k*_m_^eff^ is effective protonic conductivity of catalyst layers in [S cm^−1^]; *λ* is water content; *β* is a correction factor for the geometrical structure of membrane phase in CLs; and *x*% is the relative humidity which ranges from 40% to 100%

### Summary of Structure-Dependent Electrochemical Properties

Many experimental methods and empirical models have been employed to investigate the electrochemical properties of CLs, including exchange current density, charge transfer coefficient, electrochemical surface area, electrode roughness factor, effective electronic conductivity, and effective protonic conductivity. These electrochemical properties are vital to understanding the electrode kinetics, ohmic loss, transport phenomena, and cell performance. However, in comparison with physicochemical properties, experimental methods and apparatus for the measurement of electrochemical properties are usually complicated, and some particular properties have to be indirectly measured. Therefore, the accuracy and uncertainty analysis of the experimental methods should be carefully performed. Due to the lack of experimental data on these properties, the analytical or empirical models are very scarce for some of these parameters, including the exchange current density, charge transfer coefficient, and electrochemical surface area (or electrode roughness factor). Some correlation models are available for the effective electronic conductivity and protonic conductivity of CLs; however, the validity of most of these models should be further explored when more experimental data are available.

## Performance of Catalyst Layers

**Fig. 27:**
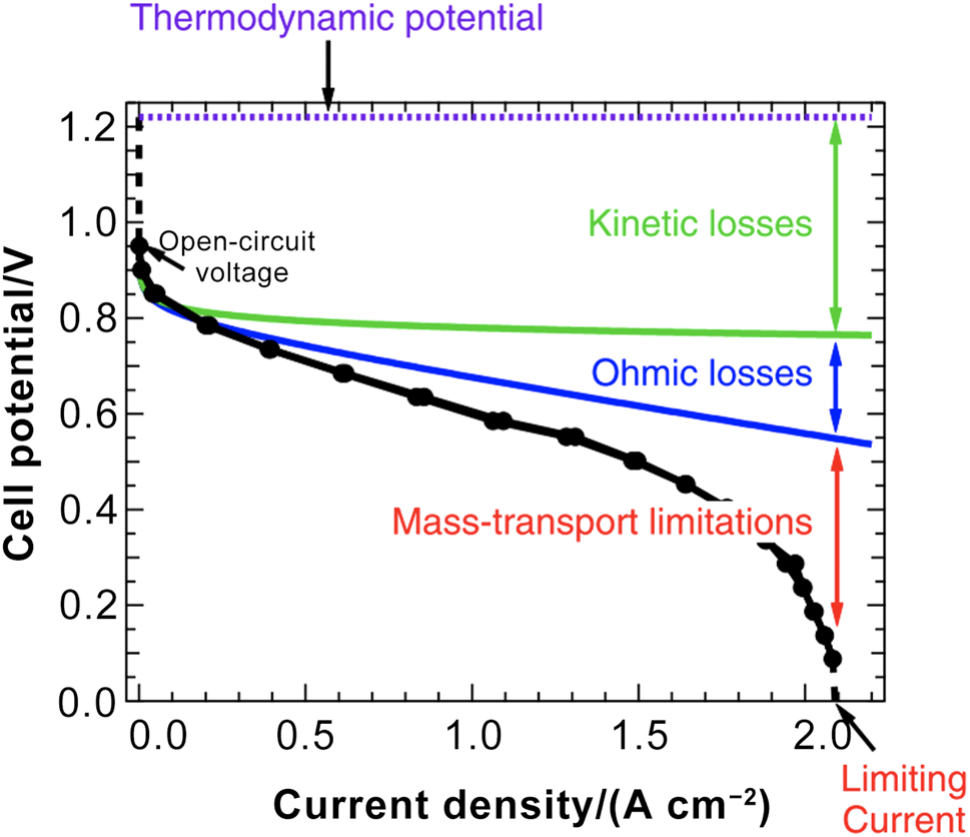
A typical polarization curve of a single PEMFC. Reprinted with permission from Ref. [268]. Copyright © 2014, the Electrochemical Society

The overall performance of the PEM fuel cells is determined by all components including membrane, CLs, GDLs, and distribution plates, among which the CLs play a dominant role. The performance is often characterized by a polarization curve for PEM fuel cells, i.e., the voltage–current relation, as shown in Fig. 27. There exists a maximum voltage if all energy stored in hydrogen and oxygen can be converted to electric energy without any losses, and this maximum voltage is called thermo-neutral voltage. However, the thermo-neutral voltage is unachievable as heat generation is always accompanied by electricity production during cell operation. Theoretically, the maximum achievable voltage under the thermodynamically reversible condition is always lower than the thermo-neural voltage and is called reversible voltage. In a practical PEM fuel cell, the output voltage will be reduced with an increase in the current density owing to four categories of irreversible polarization or energy losses: (1) reactant (fuel or oxidant) crossover and internal current, (2) activation loss, (3) ohmic loss, and (4) concentration loss, among which the latter three are closely related to the microstructure of the CLs.

### Fuel Crossover and Internal Current

Fuel crossover is a waste of hydrogen molecules by penetrating the electrolyte membrane without effective electrochemical reaction, and internal current is caused by unused electrons which are transported from anodic to cathodic electrodes directly through the membrane [5, 81]. Ideally, only protons and water can pass through the polymer electrolyte membrane, while hydrogen fuel and electrons are rejected. However, a small quantity of fuels and electrons are always possible to diffuse into the membrane from the anode, and the diffusion has a considerable influence on the open-circuit voltage (OCV), which is always smaller than reversible voltage. Each hydrogen molecule that directly crosses the membrane and reacts with oxygen at the cathode will result in two fewer electrons passing through the external circuit. In practical fuel cell operation, this type of energy loss is insignificant as the rate of fuel crossover and electron penetration is a few orders of magnitude lower than that of hydrogen consumption and electrical current in external circuits [231]. However, if the current density is very small, the voltage drop resulted from fuel crossover and internal current may not be negligible. Fuel crossover and internal current can be significantly affected by the nature of membrane material, the thickness of the membrane, and the sealing of the fuel cell stack [269].

### Activation Polarization

Activation polarization is caused by the sluggish kinetics of the electrochemical reactions in CLs, and a certain amount of energy has to be consumed to overcome the activation energy of the electrochemical reactions. The activation loss causes a sharp voltage drop when the operating current density is small, as shown in Fig. 27. The nature of catalyst materials and the nano- and microstructure of CLs determine the activation polarization. However, how the multi-scale structure of the CLs affects the activation loss has not been fully understood but a higher ECSA may help lower the activation over-potential. It should be noted that the activation over-potential is important when the operating current density is small.

### Ohmic Polarization

Ohmic polarization is caused by the electrical resistance of the fuel cells, including the proton transport resistance in the CLs and membrane, the electron transport resistance in the CLs, GDLs, and distribution plates, as well as the interfacial contact resistance between the adjacent cell components. The ohmic over-potential is reflected by the linear drop in voltage at a moderate current density region as shown in Fig. 27. The proton transport resistance in CLs caused by the transport of protons in the ionomer network can be decreased by optimizing the selection, amount, and dispersion of ionomer, as well as its corresponding solid and porous structure.

### Concentration Polarization

Concentration polarization is also known as mass transport polarization, which is caused by the lower reactant transport rate in comparison with the electrode reaction rate, leading to a low reactant concentration in the vicinity of the electrode surface. Therefore, the low reactant concentration on the electrode surface will limit the cell performance, and the output voltage drops sharply as the current density increases. The reactant transport resistance is mainly from the limited pore space in GDLs and CLs or the over-accumulated liquid water in the pores or on the electrode surface. Therefore, the microstructure of the CL and its effective properties should be comprehensively optimized.

### Summary of Catalyst Layer Performance

The PEM fuel cell performance is governed by all components including membrane, CLs, GDLs, and distribution plates, among which the CL is one of the most significant components. In a practical PEM fuel cell, the output voltage will be decreased as current density increases due to four categories of irreversible polarization or energy losses, including (1) fuel crossover and internal current, (2) activation loss, (3) ohmic loss, and (4) concentration loss. Then, the latter three are controlled by the microstructure of the CLs, which necessitates the comprehensive understanding of CL structures.

**Fig. 28:**
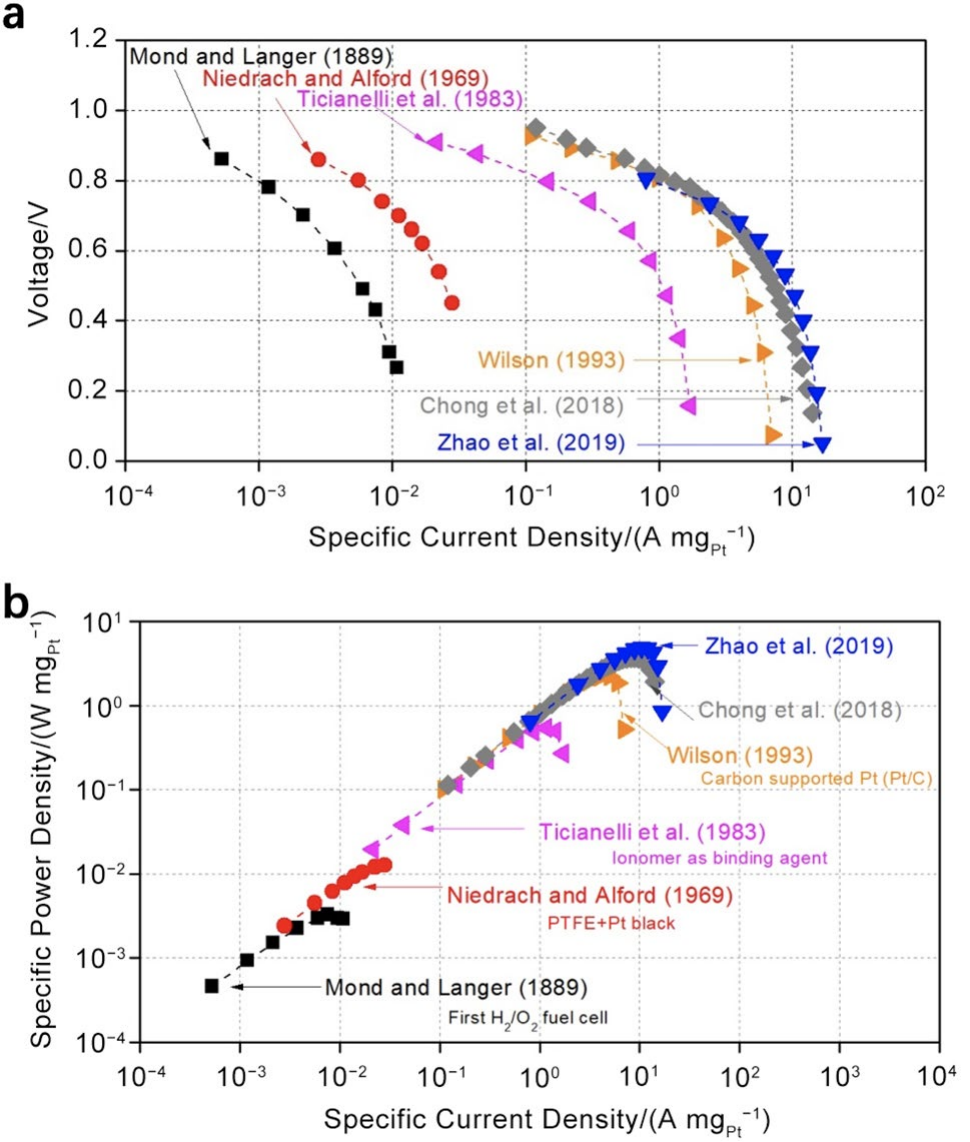
Evolution of Pt-based catalyst layer performance in PEM fuel cells: **a** voltage vs. specific power density and **b** specific power density vs. specific current density per milligram of Pt

The performance of the CLs for PEM fuel cells has been steadily improved since it was invented. As can be seen in Fig. 28, the first practical PEM fuel cell using hydrogen and oxygen as reactants invented by Mond and Langer [270] in 1889 demonstrates a low operating current of 3.8 × 10^−3^ A mg_Pt_^−1^ at 0.6 V with a maximum power density of 3.3 × 10^−3^ W mg_Pt_^−1^. For the PEM fuel cell designed by Niedrach and Alford [29] in 1969, the performance has been improved by an order of magnitude with the current density of 1.8 × 10^−3^ A mg_Pt_^−1^ at 0.6 V and a maximum power density of 1.3 × 10^−2^ W mg_Pt_^−1^. When the Pt/C is introduced to replace Pt black by Ticianelli et al. [32] in 1983, the current density has been significantly enhanced to be 0.77 A mg_Pt_^−1^ at 0.6 V with a peak power density of 0.54 W mg_Pt_^−1^. When PTFE is replaced by ionomer as the binding materials by Wilson [39] in 1993, the specific current density becomes 3.4 A mg_Pt_^−1^ at 0.6 V, while the peak power density is 2.2 W mg_Pt_^−1^. Recently, Chong et al. [271] and Zhao et al. [42] reported the high performance of PEM fuel cells with current densities of 5.2–6.7 A mg_Pt_^−1^ at 0.6 V and peak power densities of 3.7–4.9 W mg_Pt_^−1^.

Therefore, the power density of the fuel cells per milligram of Pt has been significantly improved, from around 0.003 3 to 4.9 W mg_Pt_^−1^. In other words, the amount of required noble catalysts has been considerably reduced by three orders of magnitude without sacrificing the performance, which means the cost has been dramatically decreased in comparison with that several decades ago. It should be pointed out that Chong et al.’s studies [271] suggest that the CL performance can be far beyond the 10 W mg_Pt_^−1^ based on the analysis of cathode Pt loading. The anode Pt loading employed in their studies is 0.35 mg_Pt_ cm^−2^ with commercial catalysts, while the cathode Pt loading is as low as 0.035 mg_Pt_ cm^−2^ with customized catalysts. For PEM fuel cell, the cathodic reaction is generally more sluggish, which conventionally requires more catalysts. This suggests a promising potential to reduce anode Pt loading without sacrificing too much performance, where the microstructure of the CLs will play a significant role.

## Durability of Catalyst Layers

For the long-term operation of PEM fuel cells, the performance will be deteriorated irreversibly due to gradual component degradation [272]. As the CLs determine the electrochemical reaction rates, electrical resistance, and mass transport limitation, the degradation of CLs is of great significance for the long-term performance and durability of the whole PEM fuel cell. A good CL structure should provide sufficient reaction sites, channels for reactant and water transport, pathways for electron and proton conduction, and mediums for heat transfer. Therefore, the most commonly available CLs are composed of carbon-supported catalysts, ionomer, and void space. In this section, the degradation of catalyst, carbon support, ionomer, and the CL structure, which are all vital to the fuel cell durability, is comprehensively reviewed.

### Degradation of Catalyst

**Fig. 29:**
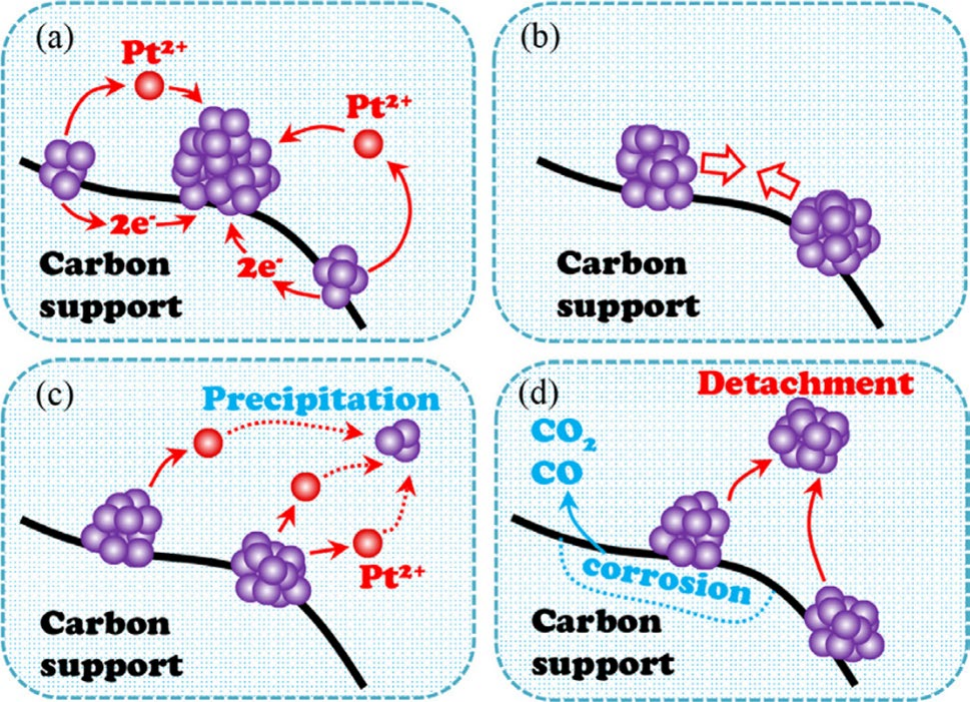
Degradation mechanisms of Pt catalyst: **a** electrochemical Ostwald ripening, **b** particle migration and coalescence, **c** Pt dissolution, and **d** Pt detachment. Reprinted with permission from Ref. [278]. Copyright © 2020, Elsevier

The most common catalyst degradation modes in PEM fuel cells include sintering [273, 274], dissolution [275, 276], and detachment [144] of catalyst nanoparticles. The sintering (coarsening or agglomeration) of catalyst nanoparticles can lead to the growth of catalyst nanoparticles and thus dramatically reduce ECSA, which is an important degradation mechanism of the long-term performance [277]. Ostwald ripening (OR) and particle migration and coalescence (PMC) are the two primary pathways of the catalyst sintering [273], as shown in Fig. 29a, b. For the OR mechanism, the small catalyst nanoparticles are broken into atoms or dissolved as charged species, and the atoms or charged species will subsequently redeposit onto the surface of the large catalyst nanoparticles [278]. For the PMC mechanism, due to the weak interaction between the catalyst nanoparticles and carbon support, catalyst nanoparticles move in a Brownian-like pattern on the support surface and consequently coalesce with each other, leading to particle growth. The microstructure change of catalyst nanoparticles on carbon supports has a strong impact on the catalyst sintering and hence the long-term performance [211, 279]. The catalyst dissolution results from the oxidation of Pt, subsequent formation of Pt ions, and final dissolution in water. This can lead to the loss of catalytic sites, reduction in electrode surface, and hence deterioration in cell performance [275], as shown in Fig. 29c. The dissolved Pt ions may be drained out of PEM fuel cell with liquid water or migrate into membranes, where the ions will be reduced by hydrogen crossover to form a band-like deposition of Pt in membranes. Macauley et al. [280] reported both positive and negative impacts of Pt dissolution and redeposition in membrane on the durability and stability [280]. A Pt band deposited in membranes can considerably increase the proton conduction resistance of the membrane, leading to a long-term degradation [40]. The detachment of catalyst nanoparticles from carbon support is another physical degradation of the catalysts, probably resulting in a permanent catalyst loss or the particle growth [144]. This can lower the active reaction sites and the catalytic performance (see Fig. 29d). As can be seen, the catalyst degradation can significantly affect the nano- and microstructure of the CLs and hence influence its short- and long-term performance.

### Degradation of Carbon Supports

**Fig. 30:**
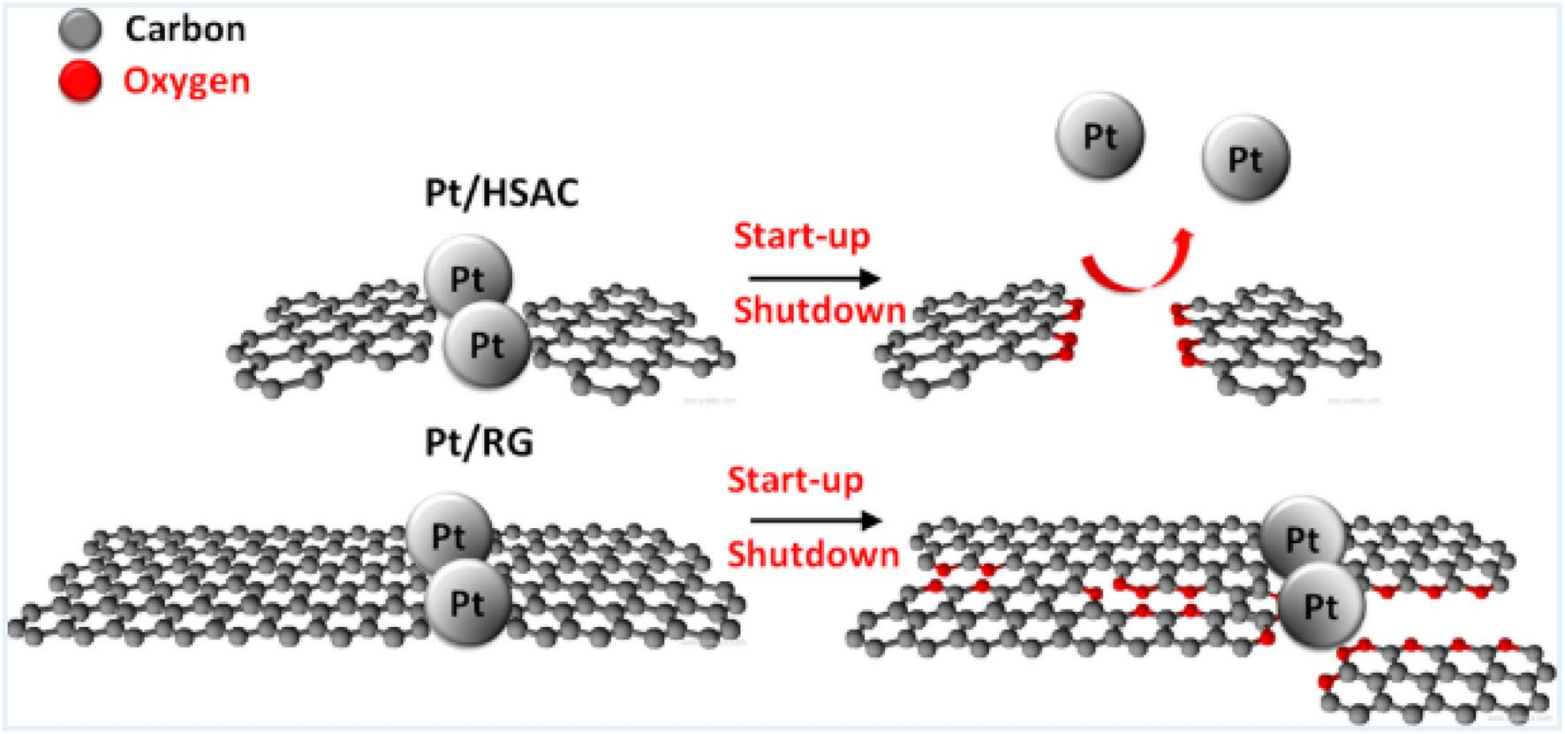
Schematic of the degradation of carbon-supported Pt based on high surface area carbon (HSAC) and reinforced-graphite (RG). Reprinted with permission from Ref. [282]. Copyright © 2015, American Chemical Society

In the actually operating environment of PEM fuel cells, carbon supports are thermodynamically unstable and subject to corrosion through the following chemical reaction at the cathodic electrodes [281], which can lead to the deterioration of the connectivity between carbon particles and the detachment of catalyst nanoparticles [3, 276], as shown in Fig. 30.

$$ {\text{C }} + {\text{ 2H}}_{{2}} {\text{O }} = {\text{ CO}}_{{2}} + {\text{ 4H}}^{ + } + {\text{ 4e}}^{ - } $$

The rate of carbon corrosion is insignificant under normal operating conditions due to the slow kinetics; however, when the fuel cells are operated with frequent start-up/shutdown and load changes, the carbon corrosion rate can be significantly accelerated due to the high cell potential (1.2–1.5 V) in local CL regions where fuel rich or fuel starvation is possible [3]. The loss of carbon supports caused by corrosion can reduce the ECSA of the CLs, leading to gradual and unavoidable performance loss as catalyst nanoparticles without carbon supports can be either washed/blown away or merged to other catalyst particles to form large particles. Besides, the corrosion of carbon supports can worsen the connectivity of the electron-conducting network, increase the electron transport resistance of CLs, and even disconnect the catalyst sites from the electric network, which will inactivate and waste the catalyst [3].

### Degradation of Ionomer

Ionomers in CLs can be decomposed due to either radical attack [269, 278, 283, 284] or thermal degradation [2]. On the electrode surface, radical species, including peroxy and hydroperoxy, can be generated as the Pt is oxidized by oxygen and water. The generated radical species will attack ionomer molecules and destroy the ionomer network. The ionomer can also degrade under high local temperatures, losing the connectivity of proton-conducting pathways. This will irreversibly increase the proton transport resistance in CLs. Ionomers also act as binding materials in CLs to stabilize Pt/C particles, meaning that without ionomers, the catalyst particles can easily move around and collide with each other to form larger agglomerates, reducing ECSA. Due to the wet and dry cycling arising from the on- and off-operation of the fuel cell [285], ionomer film can expand and shrink accordingly with the change in its hydration levels, and this may lead to the delamination at the three-phase boundary, deactivating the active sites. For a well-designed CL, the ionomer degradation is insignificant as the number of free radicals is small and can be removed by the catalyst at the TPBs.

### Degradation of Catalyst Layer Structure

The CL structure is important to ensure the high performance of PEM fuel cells by providing sufficient reaction sites, passages for reactant and product transport, media for electron and proton conduction, and pathways for heat transfer. However, the CL structure can be deteriorated with a long operation of fuel cells as a result of material degradation and interior stress cycling.

**Fig. 31:**
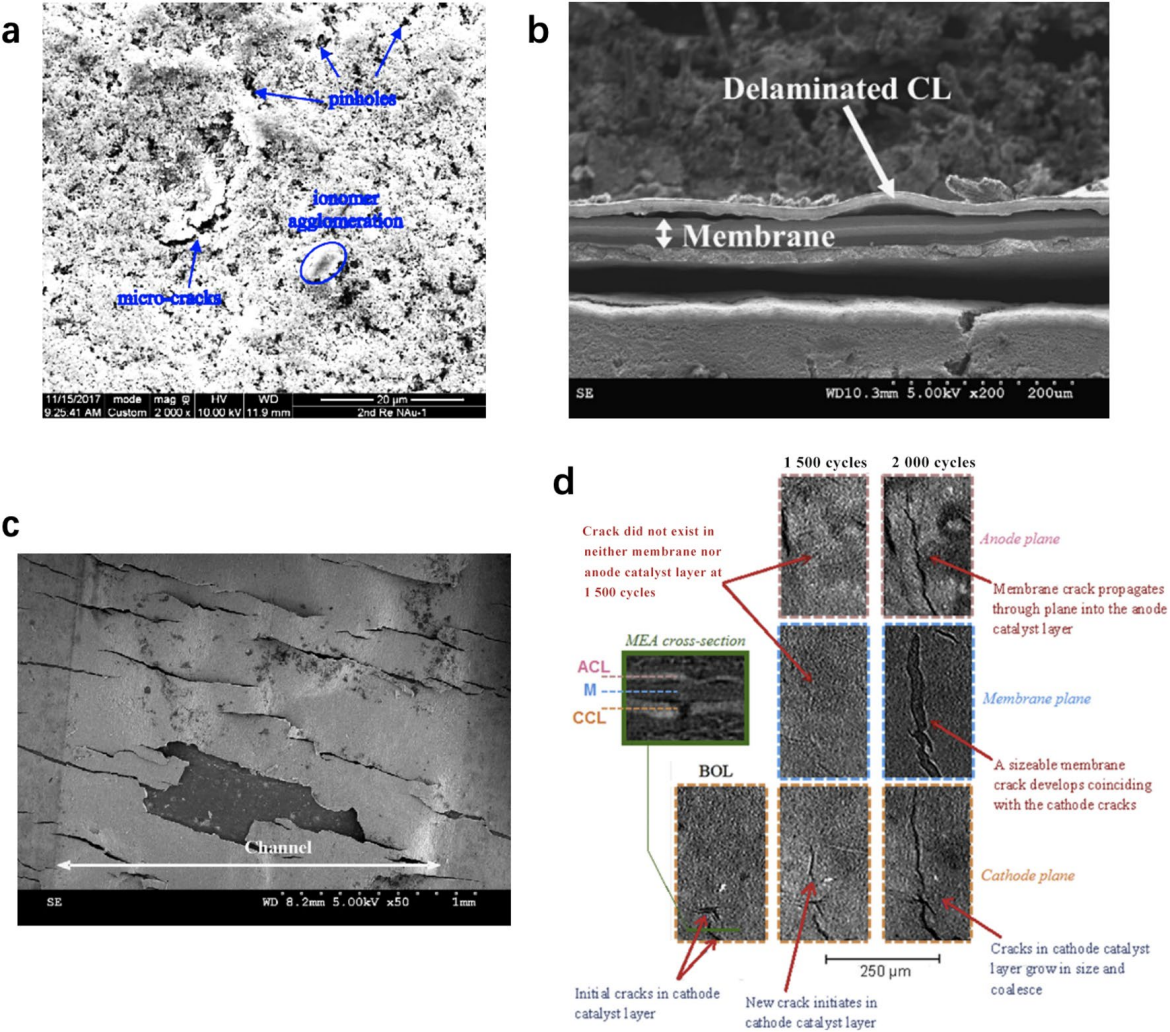
Typical structure degradation modes of catalyst layers in various studies. **a** Pinhole, crack, and agglomeration by Zhao et al. Reprinted with permission from Ref. [86]. Copyright © 2018, the Electrochemical Society. **b** Delamination due to freeze–thaw cycling by Kim et al. Reprinted with permission from Ref. [21]. Copyright © 2008, Elsevier. **c** Cracks of CLs resulted from freeze–thaw cycling by Kim and Mench. Reprinted with permission from Ref. [286]. Copyright © 2007, Elsevier. **d** Cracks resulted from relative humidity cycling by Singh et al. Reprinted with permission from Ref. [105]. Copyright © 2019, Elsevier

Typical CL structure degradation includes pinholes, cracks, agglomeration growth, and delamination. Zhao et al. [86] experimentally investigated the effect of water flooding or partial flooding on the microstructure changes of CLs. A water intrusion-evaporation cycling test is applied to a CL supported on GDLs, and after 15 cycles (equivalent to 30 h) of water treatment, Pt/C and ionomer agglomeration, pinholes, and cracks are found on the CL surface through SEM imaging (see Fig. 31a). In contrast, the water flow-through-dehydration cycling suggested that flowing water has minimal effect on particle growth as water pass through large pores more easily with the lowest transport resistance. Kim et al. [21] and Kim and Mench [286] investigated the effect of freeze–thaw cycling on the structure change of MEAs. The SEM images demonstrate delamination between CLs and membrane (see Fig. 31b) and cracks on CL surfaces (see Fig. 31c). Singh et al. [105] performed an accelerated stress test on MEA with cyclic relative humidity for inlet reactants by holding 150% relative humidity (dew-point temperature of 90 °C and operational temperature of 80 °C) for 2 min and then 0% for another 2 min. The images obtained from X-ray CT identified a large crack in the membrane, and the cracked membrane leads to corresponding cracks in adjacent CLs on both sides after 2 000 cycles (see Fig. 31d). Pinholes and cracks on the CL surfaces may cause isolation of catalyst particles, losing the connection to the electric networks, while their impact on the reactant and water transport is still under debate. Agglomeration of the catalyst particles can lead to a drop in ECSA due to catalyst sintering, and the CL-membrane interfacial delamination can significantly increase the proton transport resistance through the CL-membrane interface, which is detrimental to the overall cell performance. It should be noted that even though limited data are available in literature, the long-term changes in PSD and wettability of the CL surface are also expected as the materials degrade and the pore structure is changed during the stress cycling.

### Summary of Catalyst Layer Degradation Modes

The long-term performance of the PEM fuel cell is gradually deteriorated under different operating conditions. For instance, under steady-state operation, the performance deterioration rate can be as low as 1–2 μV h^−1^, while under accelerated stress testing conditions, the performance drop rate can be as high as 100 μV h^−1^ due to complicated cycling of voltage, current, temperature, humidity, hydration-dehydration, freeze–thaw, stress, and vibration conditions [284].

**Fig. 32:**
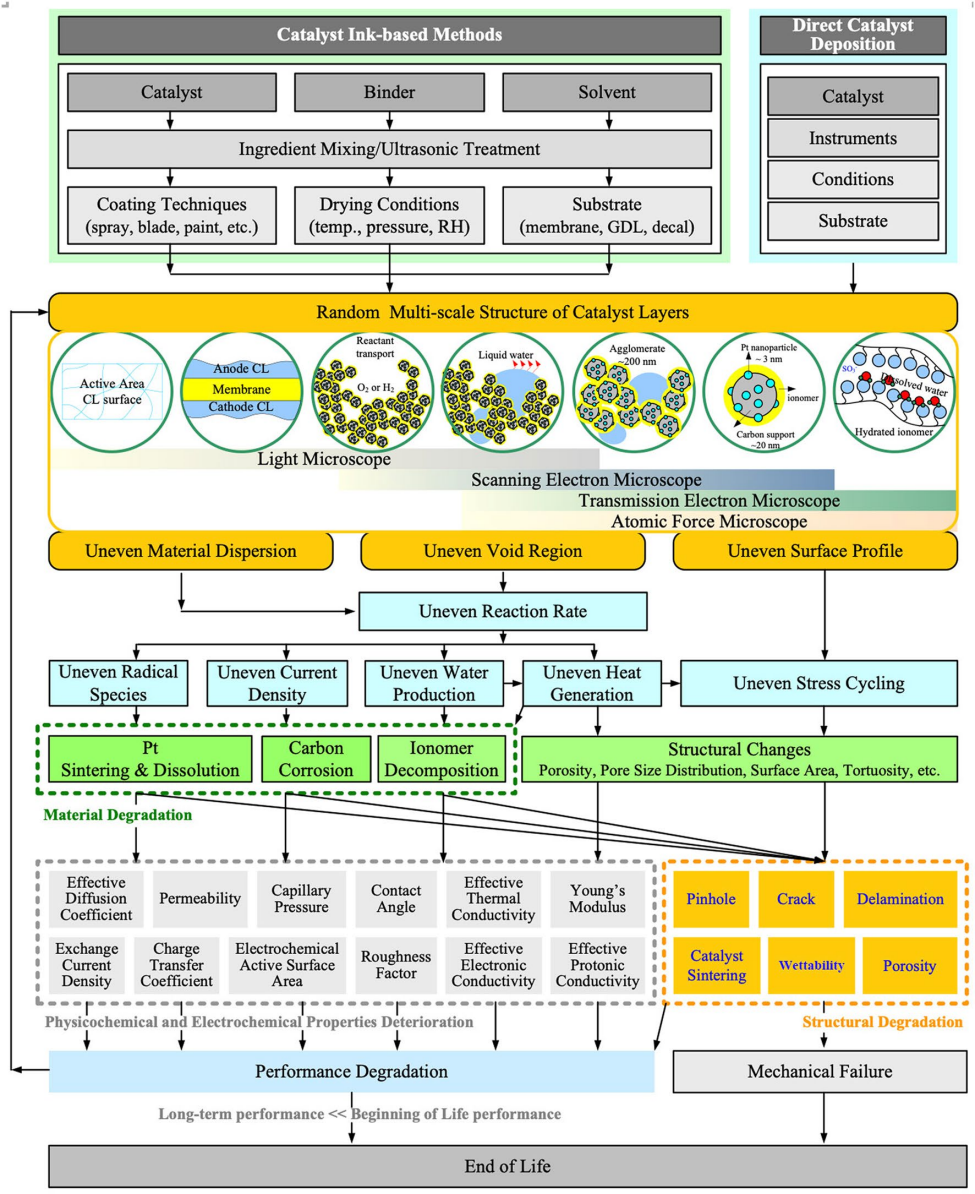
Relation between degradation modes and microstructure of catalyst layers

Many factors, including pore structure, ionomer dispersion, and particle size distribution, determine the effective physicochemical and electrochemical properties of the CLs [40, 279]. As shown in Fig. 32, the initially heterogeneous microstructure of the CLs, formed during the CL fabrication process with different techniques, consists of the essentially non-uniform solid and pore structure, including uneven material dispersion, void regions, and surface profile [49, 287, 288]. Uneven material dispersion and pore distribution can cause an inhomogeneous distribution of reactive surface, leading to significant variation in local current density, water, heat, and radical species. Excessive water may occupy reactive surface, reduce effective porosity, inhibit the transport of reactants, and even cause long-term structural changes [81, 289]. However, if too much water is exhausted owing to the local overheat or extensive reactant flow, the reactions cannot effectively proceed [5]. If the generated heat is not promptly removed, “hot spots” will be generated, which may lead to pinholes, micro-cracks, and interfacial delamination between CLs and membrane, increasing the proton transport resistance [290, 291]. Uneven distribution of current density and high-concentration radical species can worsen the non-uniform temperature and water distribution in CLs and cause carbon corrosion, Pt sintering and dissolution, and ionomer decomposition [148, 273, 279, 285, 292]. Material loss can lead to pinholes and micro-cracks, which can further propagate and cause the delamination between the membrane and CLs as a result of the mechanical, thermal, and swelling stress cycling. It should be pointed out that the material degradation and structural changes can deteriorate the physicochemical and electrochemical properties in a long-term manner, causing irreversible performance drop. However, with a deteriorated structure of CLs, the practical operating performance, material and structure, and effective properties will continue to be degraded. When the long-term performance is much smaller than the original performance or a mechanical failure occurs, the life of CLs and PEM fuel cells is ended.

## Challenges and Future Prospects

### Existing Challenges

The major challenges of the studies on CL structure in PEM fuel cells are categorized into four aspects. The first is to precisely control the CL structure formation at multi-scale levels. When the CL structure is formed, advanced experimental techniques to accurately measure the effective physicochemical and electrochemical properties require further exploration with strict uncertainty evaluation. The accurately determined structure-dependent properties should be incorporated into fuel cell modeling to understand the transport, electrochemical, and degradation phenomena in CLs. Moreover, the CL structure changes should be further explored to understand fuel cell degradation mechanisms.

1. *Controllable multi-scale structure formation*
  The structure of CL involves Pt nanoparticles (e.g., 2–5 nm), carbon supports (e.g., ~20 nm), agglomerates (e.g., 0.5–10 μm), and pores (e.g., 0.3 nm–10 μm). These elements with different length scales are mixed and non-uniformly distributed in the CLs. Therefore, it is challenging to precisely control the CL structure at multi-scale levels. Based on this review, the structure of CLs can be affected by many fabrication factors, including materials, composition, apparatus, methods, procedures, and conditions. As the fuel cell performance is sensitive to the CL structure, how to precisely control the structure formation will be very important.
2. *Experimental development and uncertainty analysis*
  The effective physicochemical and electrochemical properties of CLs are vital to understanding the electrode kinetics, transport and electrochemical phenomena, and mechanical and degradation behaviors. These properties are highly dependent on the experimental studies; however, the experimental data on these properties are not sufficiently reported in the literature, and many experimental techniques are generally designed for GDLs, which is usually several orders of magnitude thicker and stronger than CLs. The CLs, typically with a thickness of 1–30 μm for ionomer-bounded CLs, are often of non-uniform thickness. For the effective properties that require accurate average thickness, such as the effective diffusion coefficient, permeability, as well as effective thermal, electronic, and protonic conductivities, how the non-uniform thickness affects the experimental results remains unclear. Moreover, the CL is mechanically weak and has to be supported by a mechanically strong substrate before it can be ex situ measured. The substrate can be porous or non-porous, which should be carefully selected based on the specific problems to minimize measurement errors.
3. *Empirical coefficients for model accuracy*
  The structure-sensitive physicochemical and electrochemical properties of CLs are important to theoretically and numerically understand the fundamental phenomena that are difficult to be experimentally observed in PEM fuel cells. However, due to limited experimental data available for these properties, the further development of fuel cell models is challenging. For example, the exchange current density and charge transfer coefficient employed in fuel cell modeling vary significantly from case to case. Unfortunately, how these values affect the modeling results is still unclear. The modeling accuracy is also dependent on many other properties summarized in this review; however, it is challenging to have all these parameters measured at once and used in the same model and to validate the modeling results against the in situ experimental results.
4. *Multi-scale structure changes*
  The CL structure will be steadily changed as the PEM fuel cell operates, resulting in irreversible long-term performance degradation. It is challenging to quantify the effect of structural changes on the effective physicochemical and electrochemical properties of CLs and hence the performance and durability. Difficulties remain in the measurement of interior structure changes in a real-time manner and also the corresponding effective properties.

### Future Prospects

1. *Fundamentals of multi-scale structure formation*
  To precisely control the formation of the CL structure, it is essential to understand how the CL structure is formed during the fabrication process. This involves a multi-objective optimization of CL fabrication processes. Critical factors, including material, composition, fabrication techniques, procedures, and conditions, should be comprehensively studied to quantify their effects on the resultant CL structure, which should be both qualitatively and quantitatively characterized by advanced experimental techniques.
2. *Experimental studies*
  To accurately measure the structure-dependent physicochemical and electrochemical properties, experimental techniques should be specifically designed for thin, delicate, and mechanically weak CLs with various materials, composition, and structure. Optimization is needed for existing techniques to minimize experimental uncertainties.
3. *Multi-scale model development*
  Multi-scale modeling requires a comprehensive understanding of the CL structure at different scale levels. For large-scale modeling, the modeling development will be beneficial from the accurately measured effective physicochemical and electrochemical properties. For microscopic modeling, the actual structure of CLs at pore scales will bring unique insights into the theoretical and fundamental development.
4. *Observation of real-time multi-scale structure changes*
  Based on the reviews of existing studies and analysis of the technical challenges, advanced 4D microscopy technologies can be further employed to investigate the interior structure changes of CLs at multi-scale levels in a real-time manner. Benefits will be achieved for a fundamental understanding of various degradation modes resulted from CL, including catalyst sintering and detachment, material decomposition, changes in porosity and pore size, changes in wettability of the CL surface, formation of pinholes and cracks, and interfacial delamination.

### Some Fundamental Challenges

1. *“Best” or “Optimal” CL structure*
  The CL structure consists of supported catalyst, ionomer film and pore for the combined effect of reactant transport to the reaction sites, reaction product removal from the reaction sites, electron and proton transport, and the catalyzed electrochemical surface reaction for electricity generation. The transport phenomena to and from the three-phase boundary, and the electrochemical reaction at the three-phase boundary need to be balanced for optimal performance. Hence, both transport phenomena and kinetics occurring in the CL are determined by the CL structure; and it is essential to develop the “best” or “optimal” CL structure for which the “best” or “optimal” performance could be achieved for a CL made of a known set of materials, and against which a particular CL structure could be compared to determine its level or degree of the “optimalness”.
2. *Effective description of CL structure*
  The practical CL structure spans over many orders of magnitude in terms of the length scales, with various sizes of the agglomerates, ionomer films, and pores of different sizes and shapes. It is essential to find a simple and effective description for the CL structure that can determine the CL structure uniquely, and that can be used for the structural modeling and description of the CL.

## Summary and Concluding Remarks

PEM fuel cell is a promising alternative power source for vehicular, portable, and stationary applications owing to its clean and efficient energy conversion. However, its performance, durability, and cost are determined by the core component—the CL. The CL provides electrochemical reaction sites, pathways for reactant and water transport, channels for electron and proton conduction, and media for heat transfer. Therefore, the structure of the CLs plays a significant role, and a thorough understanding of the CL structure is needed.

The CL structure is formed during the fabrication process, which is governed by the material, composition, fabrication methods, procedures, and conditions. The PTFE-bonded CLs are durable as a high loading of Pt black is employed, thus leading to a very high fabrication cost. The ultra-thin CLs fabricated by the plasma sputtering, ion-beam-assisted deposition, or atomic layer deposition can minimize the use of noble catalysts; however, technical challenges such as complicated fabrication instruments and unverified durability should be further explored for mass production. The ionomer-bounded method (a.k.a. thin-film method) exhibits balanced performance, durability, and cost, which can be further optimized through improving the CL structure. The structure of CLs can be visualized by different microscopy techniques, including 2D techniques for surface structure (e.g., optical microscopy, SEM, TEM, and AFM), 3D techniques for interior CL structure (e.g., FIB/SEM and 3DX-ray CT), and 4D techniques for additional information such as chemical composition, temperature, and time in addition to 3D spatial structure. For the pore structure, the MSP, MMP, BET, and Archimedes principle have been widely used for quantitative characterization, while for the solid structure, various techniques, including XRD, ED, Raman spectroscopy, TGA, XPS, and EDS, have been broadly used for the elemental, chemical, morphology, and nanostructure analysis.

The CL structure significantly affects the physicochemical properties, which determines the transport and mechanical behaviors of the CLs. Many advanced experimental methods have been developed to investigating the physicochemical properties of CLs, including the effective diffusion coefficient, permeability, capillary pressure, contact angle, effective thermal conductivity, and Young’s modulus. The relation between the effective physicochemical properties and structural parameters has also been reviewed in this study. Many structure-based models have been established to predict these properties based on the porosity, PSD, surface area, or other structural parameters, which is important for the fundamental analysis of PEM fuel cells.

The CL structure also determines the electrochemical properties, such as the exchange current density, charge transfer coefficient, electrochemical surface area, electrode roughness factor, effective electronic conductivity, and effective protonic conductivity. The electrochemical properties are significant for electrode kinetics, ohmic loss, transport limitation, and overall performance. The experimental methods of electrochemical properties are usually indirectly measured due to the complex experimental apparatus. The uncertainty analysis for the experiments should be carefully explored to avoid misleading results. As no many organized experimental data are available, prediction models for some of these parameters, such as the exchange current density and charge transfer coefficient, and electrochemical surface area (or electrode roughness factor) are very rare. Some structure-related models are available for the effective electronic and protonic conductivity of CLs; however, these models should be further validated against a large experimental dataset.

The CL structure determines the performance and durability of PEM fuel cells. The performance of the CLs is governed by fuel crossover and internal current, activation loss, ohmic loss, and concentration loss. The fuel crossover and internal current are determined by membrane material, thickness, and sealing performance, while the activation, ohmic, and concentration losses are directly related to the CL material, composition, and multi-scale structure. The durability of PEM fuel cells can be affected by complicated operating conditions, including the cycling of voltage, current, temperature, humidity, hydration-dehydration, freeze–thaw, stress, and vibration conditions. Typical CL degradation modes include the degradation of catalyst, carbon support, ionomer, and the CL structure, which are all vital to the fuel cell durability.

Therefore, it is vital to comprehensively understand the microstructure of CLs. To accomplish this goal, the following challenges should be addressed: (1) understanding the effect of the fabrication process on the CL microstructure formation, (2) understanding the impacts of the CL microstructure on the effective physicochemical and electrochemical properties, and (3) understanding the influence of CL effective properties on the overall performance and degradation modes.

## List of symbols

*a*: Electrochemical surface area [cm^2^ mg_Pt_^−1^]
*A*: Area [m^2^]
*c*: Concentration [kg m^−3^]
*d*: Diameter [m]
*D*: Diffusion coefficient [m^2^ s^−1^]
*E*: Young’s modulus [MPa]
*f*: Stress [MPa]
*F*: Faraday’s constant [C kmol^−1^]
*g*: Gravity acceleration [m s^−2^]
*I*: Current [A]
*j*: Current density [A cm^−2^]
*j*_0_: Reference exchange current density [A cm^−2^]
*J*_m_: Mass flux [kg m^−2^ s^−1^]
*J*_n_: Molar flux [kmol m^−2^ s^−1^]
*k*_B_: Boltzmann constant [J K^−1^]
*k*_ele_: Electronic conductivity [S m^−1^]
*k*_ion_: Ionic conductivity [S m^−1^]
*k*_th_: Thermal conductivity [W m^−1^ K^−1^]
*K*: Permeability [m^2^]
*K*_0_: Intrinsic permeability [m^2^]
*K*_c_: Kozeny constant
*K*_r_: Relative permeability
*l*: Length [m]
*m*: Mass [kg]
*M*: Molecular weight [kg kmol^−1^]
*p*: Pressure [Pa]
*p*_c_: Capillary pressure [Pa]
*q*: Heat flux [W m^−2^]
*Q*: Volumetric flow rate [m^3^ s^−1^]
*r*: Radius [m]
*R*_u_: Universal gas constant [8 314 J kmol^−1^ K^−1^]
*T*: Temperature [K]
*u*: Superficial velocity [m s^−1^]
*U*: Electrical potential [V]
*V*: Volume [m^3^]

## Greek letters

*α*: Transfer coefficient
*β*: Non-Darcy coefficient [m^−1^]
*δ*: Thickness [m]
*ε*: Porosity
*η*: Over potential [V]
*θ*: Contact angle [rad]
*λ*: Mean free path [m]
*μ*: Dynamic viscosity [Pa s]
*v*: Poisson’s ratio
*ρ*: Density [kg m^−3^]
*σ*: Surface tension [N m^−1^]
*τ*: Tortuosity
*Φ*: Volume fraction
*ω*: Volume fraction of ionomer in catalyst layer

## Subscripts

act: Activation
an: Anode
b: Bulk properties
c: Capillary
ca: Cathode
eff: Effective
ele: Electronic
eq: Equivalent
g: Gas phase
geo: Geometric
Kn: Knudsen
in: Inlet
ion: Ionic
l: Liquid water
m: Membrane
mw: Membrane water
out: Outlet
p: Pore
ref: Reference state
s: Solid phase
sat: Saturation
st: Standard specimen
sub: Substrate
t: Test specimen
w: Water
